# A hypoxia-activated and microenvironment-remodeling nanoplatform for multifunctional imaging and potentiated immunotherapy of cancer

**DOI:** 10.1038/s41467-024-53906-x

**Published:** 2024-11-29

**Authors:** Jianwen Song, He Wang, Xue Meng, Wen Li, Ji Qi

**Affiliations:** 1 State Key Laboratory of Medicinal Chemical Biology, Key Laboratory of Bioactive Materials, Ministry of Education, and College of Life Sciences, Nankai University, Tianjin, China; 2 Department of Urology, The First Affiliated Hospital of Soochow University, Suzhou, China; 3 Tianjin Key Laboratory of Biomedical Materials and Key Laboratory of Biomaterials and Nanotechnology for Cancer Immunotherapy, Institute of Biomedical Engineering, Chinese Academy of Medical Sciences and Peking Union Medical College, Tianjin, China

**Keywords:** Nanomedicine, Drug delivery, Cancer therapy, Nanoparticles, Fluorescence imaging

## Abstract

Activatable theranostic systems combining precise diagnosis and robust immune activation have significant potential in cancer treatment. Herein, we develop a versatile nanoplatform integrating hypoxia-activatable molecular imaging with effective photoimmunotherapy for cancer treatment. Our molecular probe features turn-on near-infrared-II (NIR-II) fluorescence and photoacoustic signals in hypoxic tumor environments. It also induces hypoxia-triggered photodynamic and photothermal effects, promoting immunogenic cell death and activating the STING pathway, engaging both innate and adaptive immunity. The molecular probe is formulated with a vascular disrupting agent to amplify the hypoxia-responsive phototheranostic properties, on which M1-like macrophage membrane is camouflaged to shield against premature release while conferring cancer-targeting affinity. The activatable NIR-II fluorescence and photoacoustic imaging enable precise tumor delineation, while the enhanced phototherapy activates tumor-specific cytotoxic T cells, impeding both primary and distant tumor progression and providing protective immunity against rechallenge in 4T1 tumor-bearing female mice. This work advances activatable theranostic protocols for image-guided immunotherapy.

## Abstract (web summary)

Here the authors report the development and characterization of a nanoplatform that integrates hypoxia-activatable molecular imaging and photo-immunotherapy for cancer diagnosis and immunotherapy.

## Introduction

The realm of precision cancer management necessitates the advancement of strategies that facilitate both comprehensive diagnostics and efficient therapeutic interventions^1–3^. However, most currently available imaging techniques possess intrinsic advantages and drawbacks. For instance, fluorescence (FL) imaging, a promising noninvasive and non-radiative technique, demonstrates superior performance in terms of high sensitivity and cost-effectiveness, but it faces limitations in penetration depth and spatial resolution^4,5^. The recently emerging second near-infrared (NIR-II, 1000–1700 nm) window, extending beyond conventional visible and NIR (400–900 nm) region, offers improved penetration and resolution due to the reduced tissue scattering and autofluorescence^6–8^. Despite its potential, obtaining deep tissue features with microscopic spatial resolution remains a challenge for NIR-II FL imaging. On the other hand, photoacoustic (PA) imaging, a hybrid method combining optical and ultrasound imaging, offers high spatial resolution and penetration ability but suffers from low sensitivity^9–11^. Recognizing the strengths of each imaging modality, the integration of NIR-II FL and PA imaging emerges as a promising strategy, which would capture comprehensive information about the disease site. However, attaining optimal brightness concurrently in NIR-II FL and PA signals, which are respectively linked to the radiative and nonradiative channels of a chromophore, proves to be a complex task^12,13^. Additionally, the constant NIR-II FL/PA imaging with “always-on” signals, devoid of disease-specific activation, hinders their utility in selectively distinguishing between normal and pathological tissues^14,15^. Thus, there is a pressing need to develop activatable probes capable of eliciting turn-on NIR-II FL/PA dual-mode signals in response to specific pathological features for advancing sensitive and precise diagnosis.

In recent years, the landscape of cancer treatment has been evolving with the advent of immunotherapy, which leverages the patient’s own immune system to combat tumors^16–18^. However, the prevalence of immunologically “cold” tumors, characterized by low immunogenicity and inadequate immune cell infiltration, contributes to a diminished response rate to immunotherapy in clinical settings^19,20^. Moreover, immunotherapeutics typically not only stimulate antitumor immunity but also carry the risk of exacerbating T-cell reactivity against healthy organs, resulting in adverse reactions akin to autoimmune syndromes^21,22^. Consequently, more effective and selective immunotherapy strategies that can benefit larger numbers of patients safely are desperately needed.

Recently, phototherapy strategies like photothermal therapy (PTT) and photodynamic therapy (PDT) have garnered significant attention for cancer treatment^23–25^. Besides their direct cytotoxic effects on tumor cells, phototherapy has demonstrated remarkable efficacy in advancing tumor immunotherapy by inducing immunogenic cell death (ICD) of tumor cells^26,27^. ICD is a form of regulated cell death that can instigate antigen-specific adaptive immune response by firing a set of tumor-associated antigens (TAAs) or damage-associated molecular patterns (DAMPs)^28,29^. Nevertheless, there is a growing recognition that both the innate and adaptive immune systems need to be engaged to foster boosted antitumoral immunity. The activation of the cyclic GMP-AMP synthase-stimulator of interferon genes (cGAS-STING) pathway represents an emerging immunotherapy approach that targets regulators of the innate immune system^30,31^. STING activation triggers the production of type-I interferons and other immune mediators, bolstering innate immune responses and anti-tumor immunity cycle^32,33^. Intriguingly, recent studies have revealed that PDT and PTT can potentially cause mitochondrial and nuclear DNA damage, and even their subsequent leakage into the cytoplasm to activate the cGAS-STING signaling pathway^34,35^. While PDT and PTT hold promise in activating both ICD and cGAS-STING pathways, certain challenges still exist. First, the potent PDT/PTT effect is a prerequisite for effectively triggering the ICD and cGAS-STING pathways simultaneously. Among various photosensitizers, organic molecules offer the advantages of defined structure, favorable biocompatibility, and adjustable functionality. Nevertheless, insufficient PDT/PTT potency and severe photodegradation faced by most organic photosensitizers hamper their suitability for strong immunoactivation^36^. Secondly, the majority of reported photosensitizers are in “always-on” mode, and their inevitable accumulation in normal cells or skin tissue may lead to serious side effect^37^. These challenges inspire the development of intelligent organic photosensitizers with high efficacy and on-demand activated photoimmunotherapy properties.

**Fig. 1:**
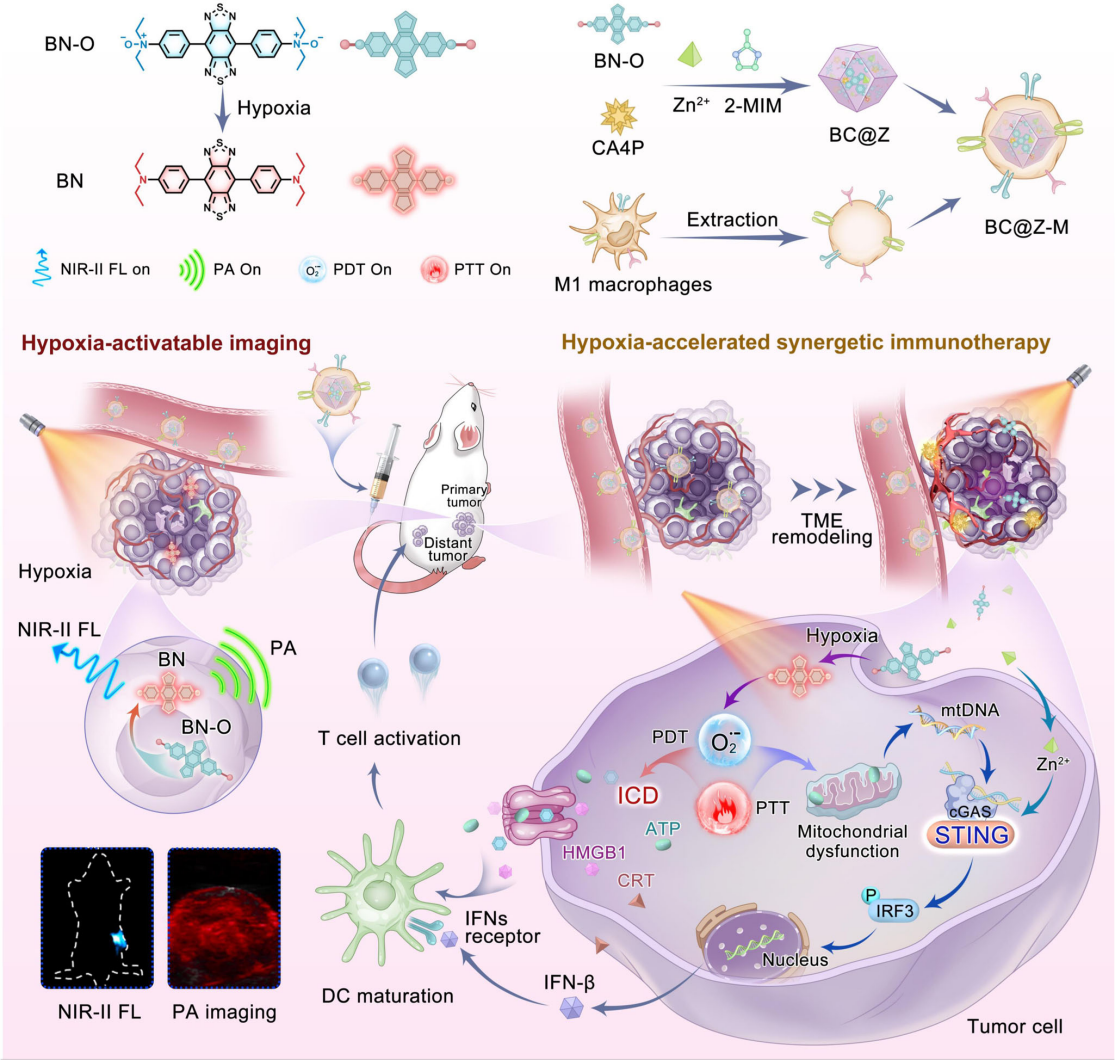
Schematic illustration of the hypoxia-activatable nanoplatform for turn-on NIR-II fluorescence and PA imaging and potentiated immunotherapy of cancer. Here a molecular probe was developed, which selectively responded to hypoxic TME and activated NIR-II FL and PA signals as well as PTT and PDT effects. By encapsulating the molecular probe and a vascular disrupting agent within an acid-degradable MOF carrier, a cancer-targeting theranostic nanoagent was formulated. The agent unleashed pronounced NIR-II FL and PA signals in hypoxic tumor to facilitate noninvasive and precise tumor imaging. Moreover, the hypoxia-triggered and TME remodeling-boosted PTT/PDT attributes synergistically activated the ICD and cGAS-STING pathways, eliciting potent immune responses and excellent antitumor outcomes.

In this work, we develop a versatile nanoplatform that integrates hypoxia-activatable multimode molecular imaging and potent photoimmunotherapy for sensitive diagnosis and efficient treatment of cancer (Fig. 1). Hypoxia is an inherent characteristic of many malignant solid tumors, stemming from the imbalance between inadequate oxygen supply due to abnormalities in tumor vasculature and the rapid consumption of oxygen by proliferating cancer cells^38,39^. Our design involves the synthesis of a molecular probe, named BN-O, based on *N*-oxide structure, which could change from an “A-A” to a “D-A” structure, enabling turn-on NIR response with robust NIR-II FL/PA signals. Beyond the switch-on imaging signal, BN-O also exhibits hypoxia-triggered type-I PDT and PTT effects. The hydrophilic molecular probe along with a vascular disrupting agent, is enclosed within an acid-degraded metal-organic framework (MOF) nanocarrier, and further camouflaged with M1-like macrophage membrane to construct a targeted theranostic nanoplatform. Specifically, the vascular disrupting agent could remodel tumor microenvironment (TME), which not only cuts off the tumor’s nutrient and oxygen supply but also exacerbates the hypoxic TME to amplify the hypoxia-activated phototherapy. In breast cancer-bearing mice, the nanoprobe successfully lights up hypoxic tumor in situ by emitting bright turn-on NIR-II FL and PA signal, facilitating sensitive and comprehensive delineation of tumor and offering crucial guidance for subsequent photoimmunotherapy. In the hypoxic milieu, the PTT/PDT attributes of the nanoprobe are also remarkably boosted, inducing strong tumor-specific T-cell immune responses and mitigating immunological resistance through the synergistic activation of both ICD and cGAS-STING pathways. Benefiting from the cascading amplification effect between the TME remodeling regime and multifaceted immunostimulation, the nanoagent not only impedes the growth of primary tumor but also hinders the progression of distant tumor, while also eliciting protective immune memory against tumor rechallenge and pulmonary metastasis. This work offers a perspective on the advancement of high-performing and activatable theranostic protocols tailored for precise image-guided enhanced tumor immunotherapy.

## Results

### Design and characterization of hypoxia-responsive molecular probe

**Fig. 2:**
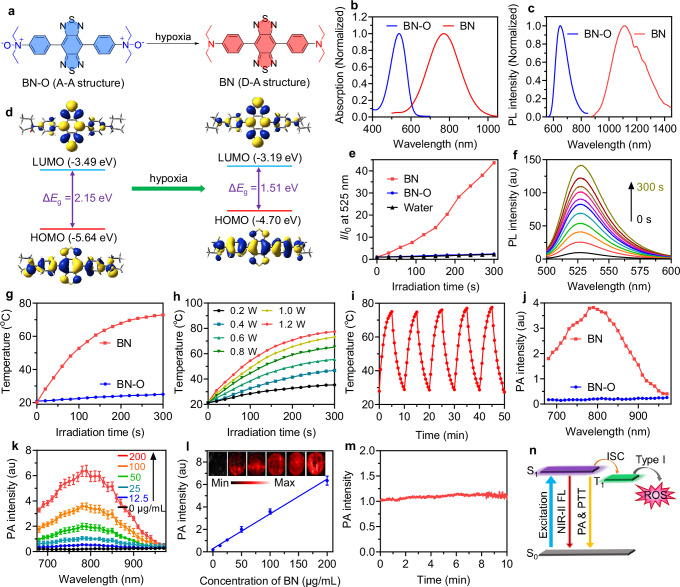
Structures and properties of BN-O and BN. **a** Chemical structures of BN-O and BN. **b** Absorption and (**c**) photoluminescence (PL) spectra of BN-O and BN in DMSO. **d** HOMO and LUMO electron cloud distribution and energy levels of BN-O and BN. **e** PL intensity of DCF at 525 nm in the presence of BN-O or BN under the irradiation of 730 nm NIR light (1.0 W cm^−2^) for different times. *I*_0_ and *I* stand for the PL intensity of DCF at 525 nm before and after 730 nm NIR light irradiation. **f** PL spectra of DHR 123 (10 µM) in the presence of BN under 730 light (1.0 W cm^−2^) irradiation for different times. **g** Temperature changes of BN-O and BN upon 730 nm light (1.0 W cm^−2^) irradiation for different times. **h** Temperature changes of BN under the irradiation of 730 nm light with different power intensities. **i** Temperature changes of BN with five circles of 730 nm laser on-off processes. **j** PA intensity of BN-O and BN at different wavelengths. **k** PA profiles and (**l**) corresponding PA intensity of various concentrations of BN at 780 nm. Data are presented as mean ± SD (*n* = 3 independent samples). **m** PA intensity of BN under PA excitation for different durations. **n** Schematic illustration of the photophysical energy transformation processes and photophysical properties of BN. For (**b**‒**l**), experiment was repeated three times independently with similar results. Source data are provided as a Source Data file.

In this work, we developed a hypoxia-responsive molecular probe based on *N*-oxide structure, which could be transformed into the corresponding amine compound under hypoxic stimuli. As presented in Fig. 2a, two *N*,*N*-diethylaniline oxides were conjugated with benzobisthiadiazole (BBTD) to yield the molecular probe (BN-O). BBTD is a highly conjugated structure with strong electron-withdrawing property, and *N*,*N*-diethylaniline oxide is also an electron-deficient unit. In hypoxic condition, *N*,*N*-diethylaniline oxide would transform into *N*,*N*-diethylaniline, forming a formidable electron-donating unit for the lone-pair electron in nitrogen atom^40,41^. As a result, BN-O molecule transformed from an “A-A” structure to a strong “D-A” structure, and the response spectral region is also expected to change during this transformation process. The synthetic pathway for BN-O is indicated in Supplementary Fig. 1. Key synthesis steps included Stille cross‒coupling reaction between *N*,*N*-diethyl-4-(tributylstannyl)aniline and 4,7-dibromobenzo[1,2-*c*:4,5-*c*’]bis([1,2,5]thiadiazole) to form BN. This was succeeded by the oxidation of aniline with meta-chloroperoxybenzoic acid (*m*-CPBA), resulting in the generation of *N*-oxide molecule, BN-O (Supplementary Fig. 2). The molecules were meticulously characterized through nuclear magnetic resonance (NMR) and high-resolution mass spectra (HRMS), as depicted in Supplementary Figs. 3–8.

We subsequently delved into the photophysical properties of these two compounds, BN and BN-O. As depicted in Fig. 2b, c, the maximal absorption and photoluminescence (PL) wavelengths of BN-O were determined to be 538 nm and 650 nm, respectively. However, BN displayed maximal absorption and emission at 770 nm and 1113 nm. The notable spectral bathochromic shift from BN-O to BN could be attributed to the formation of electron-donating aniline motif in BN. Density function theory (DFT) calculations were further conducted to interpret the electronic property. As illustrated in Fig. 2d, the electronic bandgaps of BN-O and BN were determined to be 2.15 and 1.51 eV, respectively. This result aligned well with the observed difference in response spectra, which indicated that the strong D-A interaction in BN facilitated efficient intramolecular charge transfer (ICT), thus reducing the bandgap and enabling long-wavelength absorption and emission.

The pronounced alteration in the response region of BN-O and BN motivated us to explore their NIR photodynamic and photothermal effect, two common attributes associated with phototherapeutics. As depicted in Fig. 2e and Supplementary Fig. 9, when subjected to 730 nm NIR light (1.0 W cm^−2^), BN-O solution exhibited negligible reactive oxygen species (ROS) generation, as evidenced by using dichlorodihydrofluorescein diacetate (DCFH-DA) as the ROS indicator. Conversely, in the presence of BN, the fluorescence intensity at 525 nm exhibited a rapid increase, indicative of efficient photo-triggered ROS generation by BN. Type-I and type-II PDT represent two prevailing mechanisms through which photosensitizers work. The type-II pathway is the primary mechanism in most PDT processes, wherein energized photosensitizers interact with molecular oxygen to yield highly cytotoxic singlet oxygen (^1^O_2_). In contrast, type-I PDT operates in a less oxygen-dependent manner, rendering it particularly advantageous over type-II PDT for the treatment of hypoxic solid tumors^42^. Employing 9,10-anthracenediyl-bis(methylene)dimalonic acid (ABDA) and dihydrorhodamine 123 (DHR 123) as the indicators of ^1^O_2_ and superoxide anion radical (O_2_^•−^), we found that BN exhibited robust capability to generate O_2_^•−^ while showing limited production of ^1^O_2_ under NIR light irradiation (Fig. 2f, Supplementary Fig. 10). These findings indicated that BN primarily functioned as a type-I photosensitizer, positioning it as a promising candidate for hypoxic PDT. Next, we investigated the PTT performance of BN-O and BN. Under 730 nm laser (1.0 W cm^−2^) irradiation, BN demonstrated a swift temperature increase, reaching a plateau of ~75 °C within 5 min. Conversely, the temperature of BN-O exhibited minimal change under the same NIR light irradiation (Fig. 2g). The magnitude of photo-induced temperature elevation in BN solution increased with both the laser power intensity (Fig. 2h) and molecular concentration (Supplementary Fig. 11). The photothermal effect almost didn’t change over five circles of 730 nm laser on-off processes (Fig. 2i), suggesting good photothermal stability. PA signal is closely linked to the absorption and photothermal conversion properties, thus we further investigated their PA property. Indeed, the PA spectra recorded for both BN-O and BN closely correlated with their absorption profiles, revealing a maximal PA signal at about 790 nm for BN, while negligible PA signals were detected from BN-O in the range of 680–970 nm (Fig. 2j). As depicted in Fig. 2k, l, the PA intensity showed a good linear relationship with the concentration of BN, suggesting great potential for quantitative analysis applications. Furthermore, we assessed the stability of PA signal under 10 min of laser excitation and the PA amplitude turned out to be nearly constant (Fig. 2m). Finally, the absorption and emission spectra of BN-O and BN in buffers spanning a wide pH range from 4.0 to 8.5 exhibited negligible pH-dependent effect (Supplementary Fig. 12). These properties affirmed the potential of BN as a reliable phototheranostic probe. Collectively, these findings indicated a noteworthy alteration in both electron energy levels and photophysical properties from BN-O to BN, with BN exhibiting new long-wavelength response and favorable photophysical energy transformation process conducive to activatable phototheranostic functions (Fig. 2n).

### Hypoxia-triggered molecular transformation and photophysical property change

**Fig. 3:**
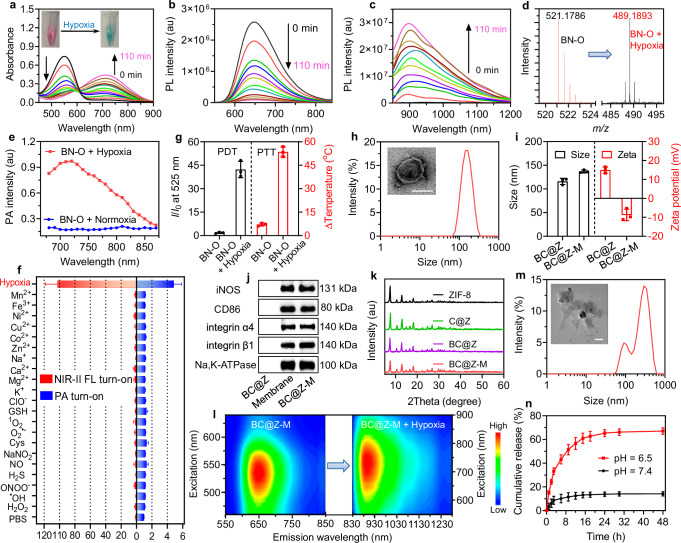
Hypoxia responsiveness of molecules and nanoparticles. **a** Absorption and (**b**, **c**) PL spectra of BN-O after hypoxic stimuli for varying time. **d** HRMS results of BN-O before and after hypoxia treatment, indicating the successful transformation into BN. **e** PA profiles of BN-O under normoxic and hypoxic conditions. **f** Relative turn-on NIR-II PL (at 950 nm) and PA intensity (at 720 nm) of BN-O (2 μM) after 2 h of incubation with various metal ions (2 mM for Na^+^, Ca^2+^, Mg^2+^, and K^+^; 50 μM for other metal ions) or reactive oxygen, nitrogen, and sulfur species (100 μM), indicating its selective response to hypoxia (*n* = 3 independent samples). **g** PDT and PTT properties of BN-O under 730 nm light (1.0 W cm^−2^) irradiation before and after hypoxia treatment (*n* = 3 independent samples). **h** Representative TEM image and DLS result of BC@Z-M. Scale bar: 100 nm. **i** The average sizes and Zeta potentials of BC@Z and BC@Z-M (*n* = 3 independent samples). **j** Western blots of BC@Z, M1-like macrophage membrane, and BC@Z-M. **k** XRD patterns of various nanoformulations. **l** Excitation-emission mappings of BC@Z-M before and after hypoxia treatment. **m** Representative TEM image and DLS result of BC@Z-M in acidic environment (pH = 6.5). Scale bars: 200 nm. **n** The release profiles of CA4P from BC@Z-M at different pH conditions (*n* = 3 independent samples). All data are presented as mean ± SD. For (**a**‒**e**, **h**, **j**‒**m**), experiment was repeated three times independently with similar results. Source data are provided as a Source Data file.

Next, the responsiveness of BN-O to hypoxia was evaluated. For this, BN-O was incubated with CYP450-rich rat liver microsomes and NADPH under oxygen-deprived conditions (nitrogen gas bubbling). The rat liver microsomes-based reductase system has been widely utilized to simulate hypoxic conditions^43^. As shown in Fig. 3a–c, under hypoxia stimuli, we observed a time-dependent enhancement in the absorption and fluorescence intensity at about 710 nm and 900 nm, respectively, accompanied by a concomitant weakening of the absorption/emission at ~550/650 nm. Remarkably, subsequent to the hypoxic reaction, the NIR-II fluorescence intensity was elevated by 112 folds. Meanwhile, the color of BN-O also visibly transformed from red to green during this process (inset of Fig. 3a), which was exactly opposite to the color change observed during the oxidation of BN to BN-O (Supplementary Fig. 2). The new absorption and fluorescence profiles aligned well with that of BN (Supplementary Fig. 13), indicating the conversion of BN-O to BN. This molecular transformation was further validated through HRMS analysis, where a new M + 1 peak of 489.1893 corresponding to BN was observed after hypoxia treatment of BN-O (Fig. 3d). The PA response of BN-O under hypoxic conditions was also assessed. In contrast to the negligible NIR PA signal observed under normoxic conditions, BN-O demonstrated a strong PA output when exposed to hypoxic stimuli, indicating its hypoxia-activated turn-on NIR PA signature (Fig. 3e). The robust turn-on NIR-II FL and PA properties position BN-O probe as a promising tool for the precise imaging of hypoxia-associated diseases. Additionally, due to the spectrally separated absorption and emission profiles of BN-O and its hypoxia-responsive product, ratiometric FL and PA imaging could be achieved by comparing their signal intensity at two distinct wavelengths. Ratiometric imaging provides inherent self-calibration, correcting for various analyte-independent factors, thereby enhancing the reliability of imaging results. We also studied the selectivity of the hypoxia-responsive probe. The BN-O solution was exposed to various interferents commonly encountered in cellular or in vivo environments, including hydrogen peroxide (H_2_O_2_), hydroxyl radical (^•^OH), peroxynitrite (ONOO^−^), hydrogen sulfide (H_2_S), nitric oxide (NO), sodium nitrite (NaNO_2_), cysteine (Cys), O_2_^•−^, ^1^O_2_, glutathione (GSH), hypochlorite (ClO^−^), and various metal ions. Notably, the treatment of BN-O with these interferents failed to produce observable switch-on signals, and only hypoxia stimuli resulted in a pronounced enhancement in the NIR-II FL and PA signals (Fig. 3f). These results validated the hypoxia-specific responsiveness of BN-O. In addition to the hypoxia-activated NIR-II FL and PA properties, BN-O also demonstrated hypoxia-boosted PDT and PTT effects (Fig. 3g). The PDT and PTT effects under 730 nm light irradiation increased by 23 and 7.5 folds after hypoxic stimuli.

### Preparation and characterization of phototheranostic nanoagent

The hypoxia-activated phototherapeutic characteristics make BN-O a promising candidate for on-demand tumor eradication. However, given the intratumoral heterogeneity, tumor cells often experience differing degrees of hypoxia, potentially influencing their responses to hypoxia-activated anticancer therapies. Integrating hypoxia-activated phototherapy with TME remodeling modality holds great promise for enhancing the precision and efficiency of tumor treatment. Recently, vascular disrupting agents, such as combretastatin-A4 phosphate (CA4P), have garnered significant interest in tumor treatment by dismantling blood vessels at the tumor site^44^. This process not only cuts off the tumor blood supply to cause nutrient deprivation in tumor cells but also intensifies the hypoxic TME, thereby accelerating the activation of hypoxia-responsive phototherapy. Consequently, the combination of BN-O with TME remodeling treatment would lead to a cascaded amplification effect in tumor killing. However, both BN-O and CA4P are small molecules, which usually exhibit poor blood circulation and inferior tumor accumulation ability after systemic administration. For instance, clinical trials involving vascular disrupting agents have failed to meet expectations, with off-target effects significantly increasing toxic side effects in vivo^45,46^.

In this study, to enhance the accumulation of BN-O and CA4P at tumor site, a tumor-targeting nanocarrier was employed. Zinc 2-methylimidazole (ZIF-8) possesses good stability under neutral physiological conditions but undergoes degradation in acidic environments, making it suitable for on-demand drug delivery in the acidic TME^47,48^. BN-O and CA4P were co-entrapped within ZIF-8-based nanocarrier via a one-pot method to create BC@Z. As demonstrated by dynamic light scattering (DLS) and transmission electron microscopy (TEM) analyses (Supplementary Fig. 14), nanosized BC@Z particles with an average hydrodynamic diameter of 110 nm were obtained. Subsequently, BC@Z underwent further coating with M1-like macrophage membrane to protect the drugs from undesirable release and confer tumor-homing capability. Studies have indicated that the α4β1 integrin on macrophage cell surface could actively bind to vascular cell adhesion molecule-1 (VCAM-1) on cancer cells^49^. Thus, the coating with α4β1 integrin-harbored macrophage membrane was expected to bestow the nanoagent with tumor-tropic properties. To achieve this, RAW264.7 cells were stimulated with lipopolysaccharide (LPS) and type II interferon (IFN-γ) for 24 h to induce M1-like macrophage polarization. Flow cytometry analysis revealed that the RAW264.7 cells stimulated by LPS and IFN-γ displayed a notable upregulation of CD86 (80.7%), a specific surface marker of M1-like macrophages (Supplementary Fig. 15), confirming their successful differentiation into M1-like macrophages. The membrane-decorated NPs (BC@Z-M) were then fabricated through repeated extrusion of BC@Z with the freshly extracted M1-like macrophage membrane. The hydrodynamic diameter of BC@Z-M experienced a slight increase to 134 nm after the membrane coating. TEM analysis revealed that BC@Z-M displayed a core-shell structure with a thin shell layer of approximately 10 nm on the surface (Fig. 3h). Simultaneously, the zeta potential of BC@Z-M markedly decreased to −8.7 mV (Fig. 3i), which was attributed to the introduction of negatively charged cell membranes. Additionally, the protein profiles of BC@Z-M were scrutinized through sodium dodecyl sulfate-polyacrylamide gel electrophoresis (SDS-PAGE), demonstrating a close match with those of M1-like macrophage membrane (Supplementary Fig. 16). Western blotting assay further confirmed the presence of typical M1-like macrophage markers, including CD86 and iNOS, along with tumor-targeting associated proteins such as α4β1 integrins, on the surface of BC@Z-M (Fig. 3j). These findings collectively indicated the successful fabrication of BC@Z-M. X-ray diffraction (XRD) measurements revealed that BC@Z and BC@Z-M exhibited similar patterns as that of ZIF-8 (Fig. 3k), indicating that the drug loading and M1-like macrophage membrane camouflage had minimal impact on the structure and crystallinity of ZIF-8. BC@Z-M demonstrated excellent colloidal stability, with negligible changes in the average diameter after storage in PBS for seven days (Supplementary Fig. 17). Moreover, BC@Z-M maintained the hypoxia-responsive molecular structure transformation (Supplementary Fig. 18) and turn-on NIR-II FL and PA properties (Fig. 3l and Supplementary Fig. 19). They also exhibited significant turn-on PDT and PTT effects after hypoxia treatment (Supplementary Figs. 20, 21), which was consistent with the properties of molecular probe. The photothermal conversion efficiency of BC@Z-M following hypoxia treatment was measured as 46.2% (Supplementary Fig. 22). We further investigated whether BC@Z-M could undergo degradation and controllable release of the encapsulated drug in acidic conditions. BC@Z-M remained stable in PBS at pH 7.4 for 48 h. In contrast, following incubation in PBS at pH 6.5 for 12 h, the NPs underwent a notable morphological transformation accompanied by changes in particle sizes (Fig. 3m). This observation indicated a pronounced collapse of ZIF-8 under acidic conditions. Additionally, only a small amount of CA4P and BN-O was released from BC@Z-M after incubation at pH 7.4 for 48 h, whereas a more accelerated drug release was observed upon exposure to weak acid environment (pH 6.5) (Fig. 3n and Supplementary Fig. 23). Moreover, the release of Zn^2+^ from BC@Z-M under different pH conditions was assessed by inductively coupled plasma mass spectrometry (ICP-MS) analysis, which suggested a substantial release of Zn^2+^ in mildly acidic environment (Supplementary Fig. 24).

### In vitro cellular investigation

**Fig. 4:**
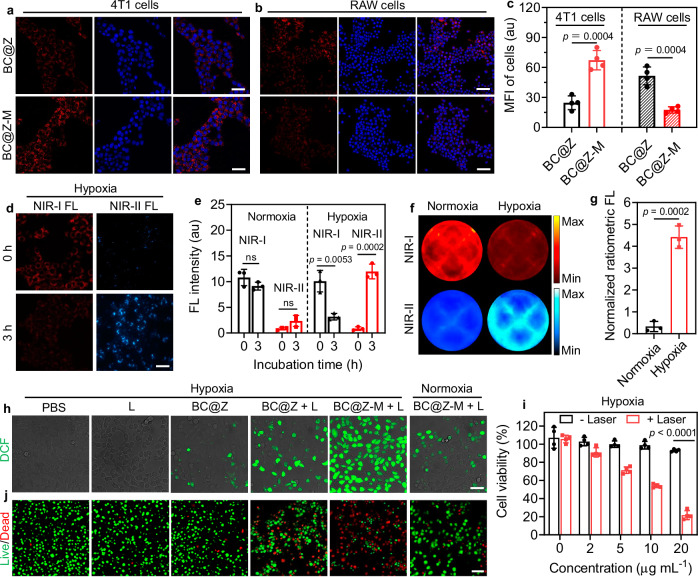
In vitro cellular investigation. Representative CLSM images of (**a**) 4T1 and (**b**) RAW cells upon incubation with BC@Z or BC@Z-M for 4 h. The cell nuclei were stained with 4’,6-diamidino-2-phenylindole (DAPI; blue fluorescence). Scale bars: 50 μm. **c** Quantitative data showing the mean fluorescence intensity (MFI) based on (**a**, **b**) (*n* = 4 independent samples). **d** NIR-I and NIR-II microscopic fluorescence images of 4T1 cells after incubation with BC@Z-M for 0 or 3 h under hypoxic condition. Scale bars: 50 μm. **e** MFI of 4T1 cells treated with BC@Z-M under normoxic or hypoxic conditions for 0 or 3 h (*n* = 3 independent samples). **f** NIR-I and NIR-II IVIS images of 4T1 cells cultured with BC@Z-M in 12-well plates under normoxic or hypoxic conditions for 3 h based on (**d**). **g** Normalized ratiometric FL of 4T1 cells treated with BC@Z-M under hypoxic or normoxic conditions based on (**f**) (*n* = 3 independent samples). **h** Representative CLSM images showing the ROS generation in 4T1 cancer cells after different treatments as indicated. Scale bar: 50 µm. **i** Cell viability of 4T1 tumor cells was assessed using MTT assay following treatment with varying concentrations of BC@Z-M under hypoxic conditions with or without 730 nm light irradiation (*n* = 4 independent samples). **j** Representative FL microscopic images showing 4T1 tumor cells co-stained with calcein-AM and propidium iodide (PI) following different indicated treatments. Scale bar: 100 μm. All data are presented as mean ± SD. For (**a**, **b**, **d**, **h**, **j**), experiment was repeated three times independently with similar results. For (**c**, **e**, **g**, **i**), statistical significance was determined using two-tailed Student’s *t*-test. Source data are provided as a Source Data file.

First, the cytocompatibility of BC@Z-M was evaluated using NIH 3T3 (mouse embryonic fibroblast) and HUVECs (human umbilical vein endothelial) cell lines. Following the treatment with varying concentrations of BC@Z-M, the viability of both cell types remained over 90%, indicating the low cytotoxicity of BC@Z-M (Supplementary Fig. 25). We proceeded to assess the hypoxia imaging and anti-tumor performance of BC@Z-M in living cells. The cellular uptake behavior of BC@Z-M in 4T1 mouse breast cancer cells and RAW264.7 cells was first investigated, with BC@Z lacking cell membrane coating used as a control. As depicted in the confocal laser scanning microscope (CLSM) images (Fig. 4a), the uptake of BC@Z-M in tumor cells was significantly higher than BC@Z without M1-like macrophage membrane modification. This outcome suggested that M1-like macrophage membrane camouflage could confer NPs with enhanced tumor cell-targeting ability. However, when the two NPs were incubated with RAW264.7 macrophage cells, weaker NP fluorescence signals were detected in the macrophage cells treated with BC@Z-M compared to those treated with BC@Z (Fig. 4b, c). Consistently, previous research has indicated that inherited proteins from macrophage membranes, such as α4β1 integrins, can actively bind to VCAM-1 on cancer cells to promote tumor-targeting, while certain surface proteins, such as CD47, can prevent undesirable macrophage-mediated phagocytosis by binding to SIRPα expressed on macrophages. The presence of these proteins, including CD47 and α4β1 integrins, on both M1-like macrophage membrane and the coated NPs was confirmed by western blotting analysis (Fig. 3j and Supplementary Fig. 26). Thus, the macrophage membrane camouflage is expected to afford both decreased clearance from the reticuloendothelial system and augmented targeting capabilities toward the tumor site.

After cellular uptake, we further investigated whether the nanoprobe could effectively detect hypoxia in living cells. 4T1 cells were incubated with BC@Z-M and cultured at 37 °C, either in a standard atmospheric environment containing 20% of oxygen (normoxic condition) or in an airtight chamber with <1% oxygen (hypoxic condition). The intracellular fluorescence signals from the nanoprobe were observed by CLSM. Under hypoxic condition, the NIR-I fluorescence intensity (excitation at 561 nm and emission at 650 nm) from the nanoprobe decreased, while the fluorescence signal in the NIR-II region (excitation at 730 nm and emission at 1000 nm) intensified with the extension of incubation time, indicating the hypoxia-induced signal transformation (Fig. 4d). In contrast, under normoxic condition, the NP fluorescence signal in NIR-I region did not diminish, and no NIR-II fluorescence signal emerged (Supplementary Fig. 27). The cells incubated under hypoxic conditions exhibited a 14.8-fold higher ratiometric fluorescence response than the normoxic control at the 3 h time point (Fig. 4e). In addition to CLSM imaging, ratiometric fluorescence imaging was also conducted on 4T1 cells cultured in 12-well plates using an in vivo imaging system (IVIS). 4T1 cells were co-incubated with 10 µM of BC@Z-M in 12-well plate under either normoxic or hypoxic conditions for 3 h, followed by imaging using IVIS. As depicted in Fig. 4f, g, the ratiometric FL response of the cells subjected to hypoxic stimulation was found to be 13.4-fold higher compared to those under normoxic conditions.

Subsequently, we assessed the anti-tumor efficacy of BC@Z-M under various conditions. The intracellular ROS generation capability of NPs was first evaluated using DCFH-DA as the ROS probe. In normoxic conditions, a subtle ROS fluorescence signal was discerned in the 4T1 cells incubated with BC@Z-M under 730 nm light (1.0 W cm^−2^) irradiation, indicative of weak PDT effect. Conversely, when the cells were subjected to BC@Z-M incubation in a hypoxic chamber, the NIR light irradiation induced greatly intensified green fluorescence (Fig. 4h, Supplementary Fig. 28). This augmentation was ascribed to the hypoxia-induced transformation of BN-O into BN, resulting in a significantly amplified PDT effect. In addition, the cells co-cultured with BC@Z-M exhibited an elevated intracellular level of ROS compared to those treated with BC@Z, which could be attributed to the enhanced NP uptake by tumor cells facilitated by the macrophage membrane coating. We further evaluated the generation of type-I ROS in 4T1 cells following “BC@Z-M + L” treatment under normoxic and hypoxic conditions. Using HPF (HyPerfluor) as the probe for hydroxyl radicals (^•^OH), we found that “BC@Z-M + L” induced obviously elevated type-I ROS levels in hypoxic conditions (Supplementary Fig. 29), consistent with the observation that BN predominantly functioned as a type-I photosensitizer in solution. These results underscored the augmented PDT effects of BC@Z-M under hypoxic stimuli, offering the potential for selective and effective treatment of hypoxic tumors.

Next, 3-(4,5-dimethylthiazol-2-yl)-2,5-diphenyl tetrazolium bromide (MTT) assays were conducted to assess the cytotoxicity of various formulations on 4T1 cells. The cells were co-cultured with BC@Z-M under either normoxic or hypoxic environment for 24 h, followed by exposure to light or not. As depicted in Fig. 4i and Supplementary Fig. 30, BC@Z-M exhibited minimal cell-killing efficacy without light irradiation, regardless of oxygen availability in the environment, indicating low dark cytotoxicity. Conversely, in the presence of 730 nm NIR light irradiation, BC@Z-M demonstrated a concentration-dependent cytotoxic effect. Notably, BC@Z-M exhibited significantly heightened light toxicity in hypoxic conditions compared to normoxic conditions. The viability of 4T1 cells subjected to BC@Z-M plus light irradiation under hypoxic conditions (21%) was three-fold lower than those administered the same dosage (20 µg mL^−1^) of BC@Z-M + light under normoxic conditions (60%). This heightened cytotoxicity could be attributed to the hypoxia-induced activation of both PDT and PTT effects of BC@Z-M. The cell viability was further assessed using a living-dead co-staining assay. As depicted in Fig. 4j, the population of propidium iodide (PI)-stained red cells significantly increased with the treatment of “BC@Z-M + L” under hypoxic conditions, indicating a substantial level of cell death. Additionally, we further evaluated cell apoptosis and death using the annexin-V and PI co-staining methods. Annexin-V staining is commonly used for detecting phosphatidylserine exposure on apoptotic cells. As demonstrated in Supplementary Figs. 31, 32, the proportion of Annexin V^+^ and PI^+^ cells significantly increased after treatment with BC@Z-M under light irradiation, indicating that “BC@Z-M + L” treatment induced a high level of cell apoptosis and death. These results, consistent with the MTT assay, underscored the potent hypoxia-activated tumor cell-killing potential of BC@Z-M under light irradiation.

### In vitro cellular immune response

**Fig. 5:**
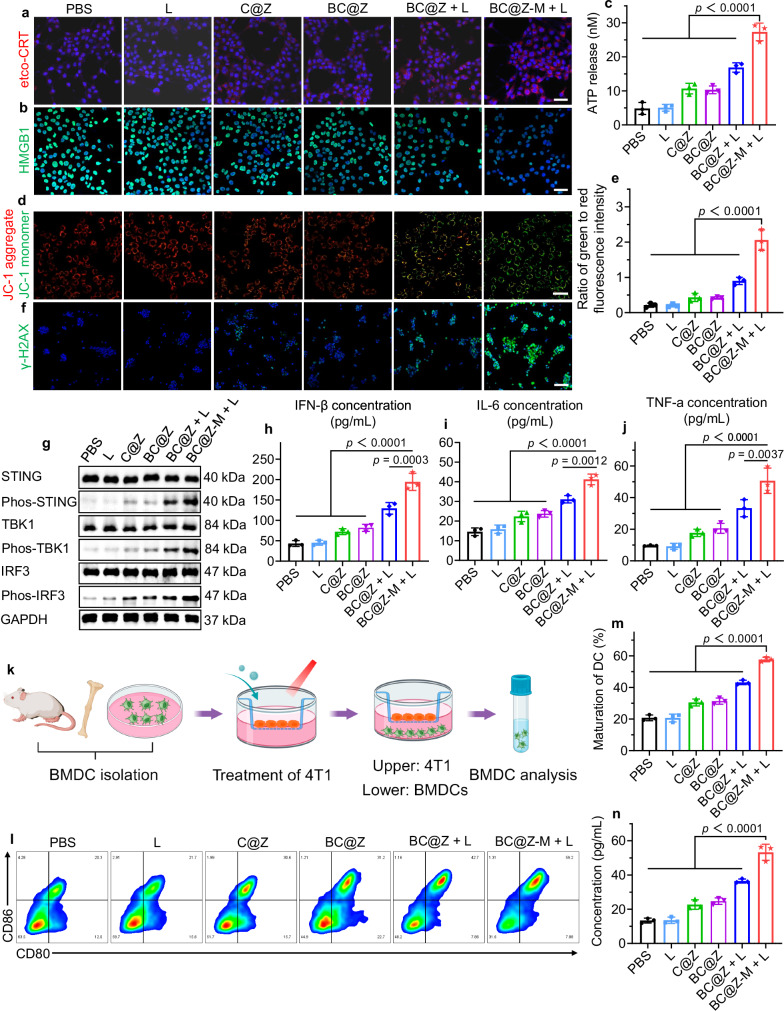
In vitro cellular immune response. Representative CLSM images showing the expression of (**a**) ecto-CRT (red fluorescence) and (**b**) HMGB1 (green fluorescence) in 4T1 tumor cells after different treatments. The cell nuclei were stained with DAPI (blue fluorescence). Scale bars: 50 μm. **c** ATP concentrations released from 4T1 tumor cells after various treatments (*n* = 3 independent samples). **d** The detection of mitochondrial potential changes in 4T1 cells after different treatments using JC-1 staining. The red fluorescence indicates JC-1 aggregate, while the green fluorescence indicates JC-1 monomer. Scale bar: 50 μm. **e** Quantitative data representing the ratio of JC-1 monomer to aggregate fluorescence for 4T1 cells treated with various formulations (*n* = 3 independent samples). **f** γ-H2AX staining of 4T1 tumor cells after different treatments. Scale bar: 100 μm. **g** Western blotting showing the expression level of cGAS-STING pathway-associated proteins in 4T1 tumor cells after different treatments as indicated. The expression levels of (**h**) IFN-β, (**i**) IL-6, and (**j**) TNF-α in 4T1 tumor cells after different treatments (*n* = 3 independent samples). **k** Schematic illustration of in vitro assessment of BMDCs maturation. Created in BioRender. Li, W. (2024) BioRender.com/d56k120. **l** Representative flow cytometry results and (**m**) quantitative analysis of mature DCs (CD 80^+^/86^+^) after different treatments (*n* = 3 independent samples). **n** The expression levels of IL-6 from BMDCs after different treatments (*n* = 3 independent samples). All data are presented as mean ± SD. For (**a**‒**j,** **l**‒**n**), experiment was repeated three times independently with similar results. Statistical significance was determined using one-way ANOVA. Source data are provided as a Source Data file.

In addition to the direct tumor cell killing, we next explored the potential of BC@Z-M to enhance anti-tumor immune responses by activating the ICD and STING pathways. Typically, during the process of ICD, dying cells release DAMPs, including surface-exposed calreticulin (ecto-CRT), high mobility group protein B1 (HMGB1), and adenosine triphosphate (ATP), which facilitate the phagocytosis of tumor cells by antigen-presenting cells (APCs) to promote tumor-specific immune responses. Here, 4T1 cells were co-cultured with various formulations under hypoxic conditions for 24 h, with or without 730 nm NIR light irradiation. Notably, immunofluorescence staining revealed a significant elevation in ecto-CRT expression on cancer cells subsequent to the treatment with BC@Z-M plus light irradiation (Fig. 5a, Supplementary Fig. 33). Specifically, for the 4T1 cancer cells treated with BC@Z-M + light, the ecto-CRT expression was 3.1- to 1.5-fold higher than that of other groups. Furthermore, a pronounced translocation of HMGB1 from the nuclei to the extracellular space was observed in the cells treated with “BC@Z-M + L” (Fig. 5b). HMGB1 release in the cell supernatant was further detected using ELISA assay, revealing a significant release from 4T1 cells after “BC@Z-M + L” treatment (Supplementary Fig. 34), which was consistent with the immunofluorescence staining results. The supernatants of 4T1 cells treated with “BC@Z-M + L” also exhibited the highest concentration of ATP compared to other groups (Fig. 5c). These findings collectively suggested that BC@Z-M robustly induced ICD in tumor cells when exposed to NIR laser under hypoxic conditions.

The activation of STING in cancer cells is conducive to instigating the secretion of interferons and proinflammatory cytokines to promote dendritic cells (DCs) maturation as well as T cells activation, thus reinforcing antitumor immune cycles^50,51^. Given the potent PDT and PTT capabilities exhibited by BC@Z-M under hypoxic stimuli, they show high potential for inducing the mitochondrial and nuclear DNA damage, leading to their leakage into the cytoplasm and subsequent activation of STING pathway^52^. Moreover, compelling evidence has suggested that free Zn^2+^ could augment the STING pathway by promoting cyclic GMP-AMP (cGAMP) production and enhancing the cGAMP-STING binding affinity^53–56^. Consequently, we anticipated that the synergistic combination of BN-O-based phototherapy with the Zn^2+^-releasing ZIF-8 nanocarrier would significantly elevate the activation of cGAS-STING signaling.

We first examined the influence of BC@Z-M + light irradiation on mitochondrial membrane potential using JC-1 staining. As shown in the representative CLSM images (Fig. 5d) and corresponding quantitative analysis (Fig. 5e), compared to other groups, the BC@Z-M + L-treated cells displayed distinctly brighter green JC-1 monomer fluorescence, suggesting significant mitochondrial dysfunction. We then assessed the DNA damage using the γ-H2AX marker (Fig. 5f). In both the light irradiation or NPs alone groups, no discernible fluorescence signal was observed. Nevertheless, the “BC@Z-M + L” group displayed a substantial fluorescent signal, with the intensity of γ-H2AX foci signals being 5.89, 5.51, 4.07, 3.80, and 1.91-fold higher than that in the PBS, L, C@Z, BC@Z, and BC@Z + L groups, respectively (Supplementary Fig. 35). We further investigated the DNA oxidation by staining 8-OHdG, a well-known biomarker of DNA oxidation. The “BC@Z-M + L” treatment induced significant DNA oxidation, whereas the untreated control, light alone, and NPs alone group exhibited only minimal levels of DNA oxidation (Supplementary Fig. 36). Oxidized DNA usually shows enhanced stability in the presence of DNase^57^, which would facilitate its transport and activate the cGAS-STING signaling pathway. Subsequently, the expression of STING pathway-associated proteins was analyzed via western blot. The results revealed that the “BC@Z-M + L” treatment markedly promoted the up-regulation of phosphorylated STING (P-STING), phosphorylated TBK1 (P-TBK1), and phosphorylated IRF3 (P-IRF3) in 4T1 cells (Fig. 5g). These outcomes signified the effective activation of STING pathway by BC@Z-M under light exposure. Following this, the levels of cytokines, including interferon-β (IFN-β), tumor necrosis factor-α (TNF-α), and interleukin-6 (IL-6) in the cell supernatant were evaluated using ELISA kit. Consistently, the cells treated with “BC@Z-M + L” exhibited the highest secretion levels of these proinflammatory cytokines (Fig. 5h–j). Notably, knocking down STING in 4T1 tumor cells led to a 2.28-fold reduction in IFN-β secretion in “BC@Z-M + L” group, while STING overexpression further amplified IFN-β secretion (Supplementary Fig. 37). These findings indicated that the STING pathway played a critical role in mediating IFN-β secretion. CXCL10 is a crucial cytokine produced downstream of IFN following STING activation, which facilitates immune cell recruitment^58^. We measured the CXCL10 levels using ELISA following various treatments. The results indicated a significant increase in CXCL10 release from cells after “BC@Z-M + L” treatment (Supplementary Fig. 38).

Following the confirmation of ICD induction and STING pathway activation, their consequential biological impact on DC maturation, a pivotal step in the initiation of antitumor immune responses, was investigated. As illustrated in Fig. 5k, bone marrow-derived dendritic cells (BMDCs) were isolated from the tibia and femur of BALB/c mice. Then 4T1 cancer cells were seeded onto the upper chamber of transwell, subjected to various treatments. Afterward, BMDCs were co-cultivated in the lower transwell compartment. After 24 h incubation, they were collected for the evaluation of maturation markers using flow cytometry. Impressively, the co-incubation of BMDCs with 4T1 cancer cells pretreated with “BC@Z-M + L” markedly increased the expression of maturation markers CD80 and CD86, indicating strong DCs maturation (Fig. 5l and Supplementary Fig. 39). The population of CD80^+^CD86^+^ BMDCs in the “BC@Z-M + L” group surpassed that of the PBS, L, BC@Z, and BC@Z + L groups by 2.78, 2.79, 1.84, and 1.34 times, respectively (Fig. 5m). Furthermore, the supernatant of BMDCs was collected, and the levels of TNF-α, IL-6, and IFN-β were evaluated using ELISA kits. The results revealed significantly elevated concentrations of these proinflammatory cytokines in the “BC@Z-M + L” group compared to other groups (Fig. 5n and Supplementary Fig. 40). STING knockdown in tumor cells significantly compromised the ability of “BC@Z-M + L”-pretreated tumor cells to induce BMDC maturation, while STING overexpression boosted their capacity to activate BMDCs following “BC@Z-M + L” treatment (Supplementary Fig. 41). To further determine whether other DNA sensors (such as AIM2) or RNA sensors (RIG-I, MDA-5) contribute to the activation of type-I interferon pathways and BMDC response, we conducted knockdown experiments targeting these sensors. The results revealed that silencing AIM2, RIG-I, or MDA-5 did not significantly reduce IFN-β secretion from the “BC@Z-M + L”-treated 4T1 tumor cells as well as their capacity to trigger BMDC maturation (Supplementary Fig. 42). These findings suggested that the STING pathway played an important role in the immune response, while AIM2, RIG-I, and MDA-5 might have weak involvement. In conclusion, BC@Z-M plus light irradiation could effectively activate the ICD and STING pathways in tumor cells, fostering the release of DAMPs and proinflammatory cytokines, which, in turn, initiated DCs maturation−a crucial step for the therapeutically relevant immune response.

In addition to 4T1 breast carcinoma cell line, we further validated our findings in other tumor models, including the B16-F10 melanoma and MC38 colon carcinoma models. As shown in Supplementary Fig. 43, co-incubation of BMDCs with B16-F10 or MC38 cancer cells pretreated with “BC@Z-M + L” markedly increased the expression of maturation markers. Additionally, the level of IFN-β in the supernatant of BMDCs was significantly elevated in “BC@Z-M + L” group compared to other groups. We also employed B16-OVA tumor cells, which express the model antigen ovalbumin, to investigate the antigen-specific immune response (Supplementary Fig. 46). Flow cytometry analysis indicated that B16-OVA cells pretreated with “BC@Z-M + L” induced the strongest antigen presentation (DC displaying H-2Kb-SIINFEKL) (Supplementary Figs. 44 and 46b, c) and BMDC maturation (CD80^+^CD86^+^ DC) (Supplementary Fig. 46e, f) among all groups. Additionally, the IFN-β expression was significantly elevated in “BC@Z-M + L” group (Supplementary Fig. 46d). Considering that MHC/antigen cross-presentation and costimulatory signals are crucial for inducing tumor-specific cytotoxic T lymphocyte (CTL) responses, we further assessed CTL activation. BMDCs pretreated with B16-OVA cells under various treatments were co-cultured with CD8^+^ T cells isolated from OT-1 mice, which have T-cell receptors specific for the ovalbumin-derived peptide SIINFEKL. A CFSE staining assay was used to evaluate CD8^+^ T cell proliferation, revealing significant proliferation of antigen-specific CD8^+^ T cells in BC@Z-M + L group (Supplementary Figs. 45 and 46g, h). Additionally, the percentage of IFN-γ^+^ CD8^+^ T cells was markedly increased after co-culture with BMDCs pretreated with “BC@Z-M + L”-killed B16-OVA cells (Supplementary Fig. 46i, j), indicating robust CD8^+^ T cell activation. We then assessed the ability of these activated CD8^+^ T cells to kill B16-OVA tumor cells using LDH cytotoxicity assay. The results suggested that the activated CD8^+^ T cells exhibited effective target cell killing activity, with the highest tumor cell lysis and LDH release observed in “BC@Z-M + L” group (Supplementary Fig. 46k). These findings confirmed the robustness of our treatment in eliciting strong immune response in different tumor models.

### In vivo activatable multimodal imaging of hypoxic tumors

**Fig. 6:**
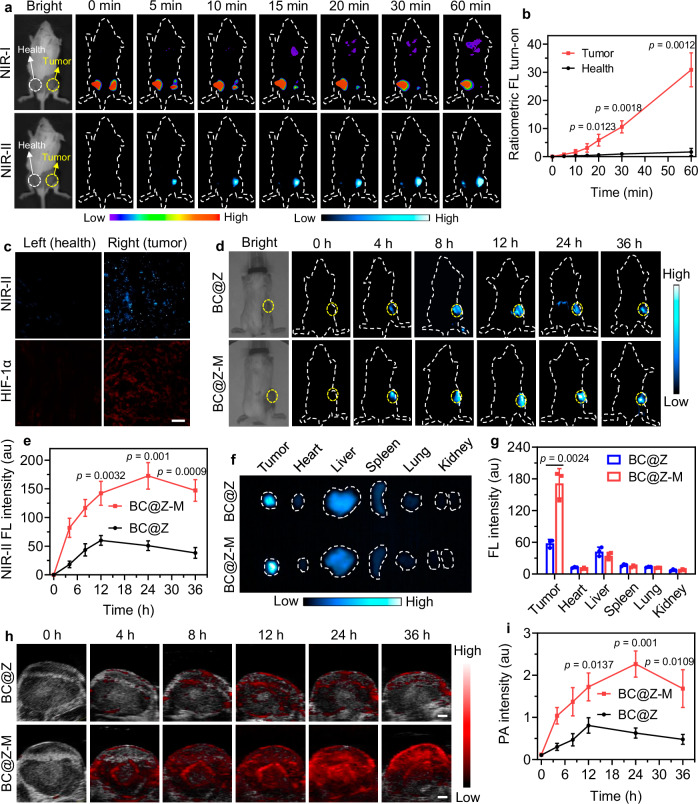
In vivo NIR-II FL and PA imaging of hypoxic tumor. **a** Representative in vivo NIR-I FL (excitation: 530 nm, emission: 600 nm) and NIR-II FL (excitation: 730 nm, emission: 1000 nm) images obtained from 4T1 tumor-bearing mice at various time points following intratumoral injection of BC@Z-M. As control, BC@Z-M was also administered via subcutaneous injection into the left flanks of mice (without tumor inoculation). Yellow arrows denote the tumor, while white arrows indicate the healthy control flank. **b** Ratiometric turn-on FL of tumor and healthy sites of 4T1 tumor-bearing mice after injection of BC@Z-M for different time based on (**a**) (*n* = 3 mice). **c** Representative microscopic images of tissue sections showing the NIR-II FL activation of BC@Z-M and elevated hypoxia, as indicated by HIF-1α staining, in the tumor tissue when compared to healthy tissue. Scale bar: 100 µm. **d** Representative NIR-II FL images and (**e**) corresponding tumor NIR-II FL intensity of 4T1 tumor-bearing mice after i.v. injection of BC@Z or BC@Z-M for different time (*n* = 3 mice). **f** Representative NIR-II FL images and (**g**) corresponding NIR-II FL intensity of major organs and tumors from mice at 24 h post i.v. injection of BC@Z or BC@Z-M (*n* = 3 mice). **h** Representative PA images and (**i**) corresponding PA intensity of tumor site in 4T1 tumor-bearing mice after i.v. injection of BC@Z or BC@Z-M for various time (*n* = 3 mice). Scale bars: 1 mm. All data are presented as mean ± SD. For (**a**, **c**, **d**, **f**, **h**), experiment was repeated three times independently with similar results. Statistical significance was determined using two-tailed Student’s *t*-test. Source data are provided as a Source Data file.

Encouraged by the in vitro hypoxia-activated turn-on NIR-II FL and PA properties of BC@Z-M, we proceeded to explore their potential for multimodal imaging of hypoxic tumors in vivo. Tumor allografts were established by subcutaneously implanting 4T1 tumor cells into the right flanks of BALB/c mice. In an initial investigation aimed at validating the real-time NIR-II fluorescence activation of BC@Z-M in hypoxic tumors, BC@Z-M was administered to tumor-bearing mice through direct intratumoral injection. As a control, an equivalent dose of BC@Z-M was also injected subcutaneously into the left flanks (without tumor inoculation) of the same BALB/c mice. The fluorescence changes at both the tumor site and the contralateral subcutaneous site were monitored using an IVIS and a home-built, in vivo NIR-II fluorescence imaging system. As depicted in Fig. 6a, the tumor site injected with BC@Z-M exhibited a gradual reduction of NIR-I fluorescence signal (under 530 nm excitation), accompanied by the concurrent increase of the NIR-II fluorescence signal collected at 1000 nm (under 730 nm light irradiation). This observation signified the real-time intratumoral activation and conversion of the hypoxia-responsive probe. In contrast, there was no notable decline in NIR-I fluorescence or appearance of NIR-II signal in the BC@Z-M-injected healthy left flanks. As a result, the ratiometric fluorescence intensity at the tumor site was greatly higher than the healthy control site (Fig. 6b). The right tumor and left healthy tissues were further sectioned and subjected to staining with hypoxia-inducible factor 1α (HIF-1α) to detect hypoxia. Indeed, we noted a consistency between the heightened NIR-II fluorescence intensity emitted by BC@Z-M and the elevated hypoxia, as indicated by HIF-1α staining, in the tumor tissue (Fig. 6c). These findings implied that BC@Z-M could function as a turn-on NIR-II fluorescence imaging probe for hypoxic tumor detection.

After confirming the hypoxia-responsive activation of BC@Z-M at the tumor site, we proceeded to assess its capability to illuminate the tumor when administered intravenously. The in vivo pharmacokinetics, biodistribution, and clearance process of the nanoagents were first evaluated. The NPs were labeled with a NIR dye, 1,1-dioctadecyl-3,3,3,3-tetramethylindotricarbocyanine iodide (DiR). After intravenous injection, blood samples were collected at various time points to measure the fluorescence intensity of the NPs. As depicted in Supplementary Fig. 47, BC@Z-M demonstrated a circulation half-life of about 15 h, with nearly all NPs cleared from the bloodstream by 48 h post-injection. At 24 h after intravenous injection, major organs were collected for ex vivo fluorescence imaging using IVIS (Supplementary Fig. 48). Both BC@Z and BC@Z-M showed obvious accumulation in liver due to the unavoidable NP capture by the reticuloendothelial system. Noteworthy, tumors of the mice injected with BC@Z-M displayed significantly higher signals compared to those injected with BC@Z, demonstrating enhanced tumor-targeting ability mediated by the membrane coating. To evaluate the clearance, feces, and urine of the mice injected with the DiR-loaded BC@Z-M were collected at different time points post NP administration. As shown in Supplementary Fig. 49, strong fluorescence signals were detected in the feces rather than in the urine on days 1 and 3. By day 7, fecal fluorescence markedly decreased, indicating that BC@Z-M was predominantly excreted via the biliary pathway^59,60^.

Next, we assessed the possibility of using hypoxia-activated NIR-II imaging to specifically detect tumor. BC@Z-M or BC@Z were intravenously injected into the tumor-bearing mice via tail vein, followed by NIR-II fluorescence imaging conducted at various time intervals post-injection. As depicted in Fig. 6d, the mice administered with BC@Z-M exhibited robust NIR-II fluorescence signals at the tumor site, reaching a peak at approximately 24 h post-injection. In contrast, the tumor of mice injected with BC@Z lacking membrane coating displayed considerably weaker NIR-II signals, attributed to their inadequate tumor-targeting ability. Quantitative analyses revealed that the maximal NIR-II signal in the tumor for BC@Z-M group was 3.4 times higher than that of BC@Z group (Fig. 6e). The mice were euthanized 24 h post-injection, and major organs, including heart, liver, spleen, lungs, and kidneys, and tumor, were harvested for ex vivo fluorescence imaging and quantitative analysis. Notably, bright NIR-II fluorescence signal was observed in the tumor region, surpassing even that in liver (Fig. 6f, g). It is well-documented that liver usually exhibits the high NP signal after systemic NP administration due to the sequestration of NPs by the reticuloendothelial system. In this study, the heightened signal of BC@Z-M observed in the tumor tissue, in comparison to the liver, could be attributed to both their effective accumulation in the tumor and the hypoxia-responsive NIR-II activation within the tumor site.

Inspired by the robust in vitro PA output of BC@Z-M in response to hypoxia stimuli, our focus shifted towards utilizing BC@Z-M for the detection of hypoxic tumors through PA imaging. The tumor-bearing mice were intravenously administered either BC@Z-M or BC@Z via tail vein injection, and PA imaging was conducted at different time points after injection. In the BC@Z-M-administrated mice, the PA signal in the tumor region exhibited a significant increase over time, peaking at approximately 24 h (Fig. 6h), consistent with the findings from NIR-II fluorescence imaging. At 24 h after injection, high-contrast PA signal with a very high signal-to-noise ratio (19.4) was observed in the tumor tissue. Compared to the BC@Z-M group, the PA signal at the tumor site of mice injected with BC@Z was diminished due to its limited tumor-targeting ability (Fig. 6i). It was noted that the resolution of PA imaging outperformed that of fluorescence imaging, allowing for enhanced visualization of the distribution of PA signals. This superiority stems from the fact that acoustic waves scatter several orders of magnitude lower than light in tissues, endowing PA imaging with deeper penetration depth and superior spatiotemporal resolution compared to fluorescence imaging. These findings demonstrated the great potential of BC@Z-M for both NIR-II FL and PA imaging of hypoxic tumor, allowing for precise and sophisticated delineation of tumor.

### In vivo anticancer study

We subsequently assessed the in vivo antitumor therapeutic efficacy of BC@Z-M in 4T1 tumor-bearing mice. First, the tumor prophylactic vaccination experiment was conducted to test whether the “BC@Z-M + L”-treated tumor cells had real immunogenicity in vivo. As shown in Supplementary Fig. 50, we vaccinated mice with 4T1 cancer cells pre-treated with PBS, BC@Z, BC@Z + L, or BC@Z-M + L, or no vaccine on day 0, and subsequently challenged them with live cancer cells on day 7. Tumor growth at the injection site was monitored every two days. Prophylactic vaccination with 4T1 cells pre-treated by “BC@Z-M + L” effectively protected the mice against subsequent tumor cell challenge, evidenced by the obviously delayed tumor growth compared to other groups. These results indicated the robust immunogenicity and anti-tumor immunity elicited by cancer cell death induced by BC@Z-M + L.

**Fig. 7:**
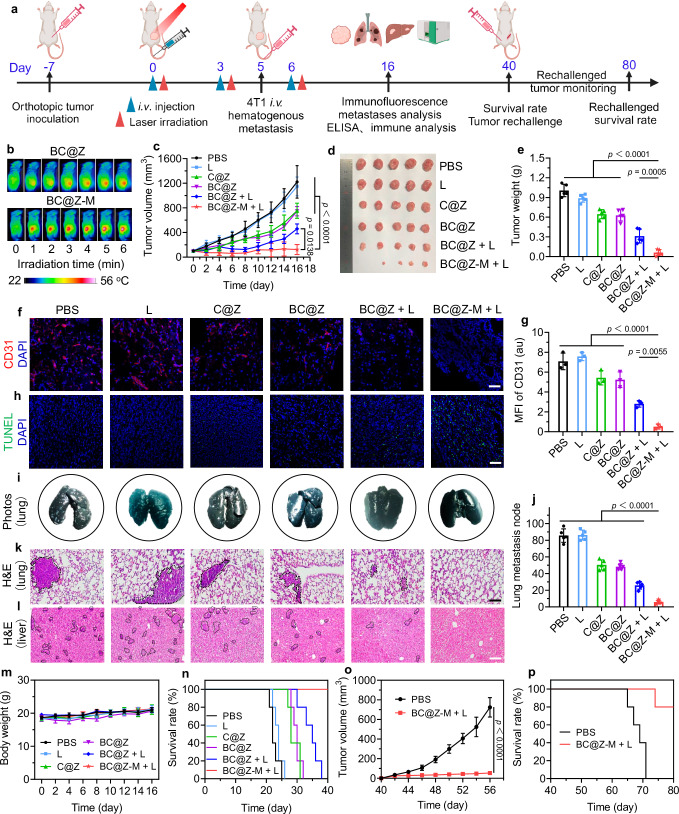
Assessment of the therapeutic efficiency of BC@Z-M in vivo. **a** Schematic illustration showing the treatment procedure and assessment of therapeutic efficacy of BC@Z-M in 4T1 tumor-bearing mice. Created in BioRender. Li, W. (2024) BioRender.com/d03e195. **b** Temperature changes at the tumor site of mice injected with BC@Z-M or BC@Z under 730 nm light (1.0 W cm^−2^) irradiation. **c** Tumor growth curve, (**d**) tumor photographs, and (**e**) tumor weights of different treatment groups (*n* = 5 mice). **f** Representative CLSM images of the tumor sections stained with CD31, and (**g**) corresponding MFI of CD31 in tumor sections from mice receiving various treatments (*n* = 3 mice). Scale bar: 100 μm. **h** TUNEL staining images of tumor slices collected from the mice in different groups. The cell nuclei were stained with DAPI (blue fluorescence). Scale bar: 100 μm. **i** Representative photographs of India ink-stained lungs harvested from the mice in different groups. **j** Quantification of the lung metastasis nodes in different groups (*n* = 5 mice). Representative H&E staining images of (**k**) lung and (**l**) liver sections from mice subjected to various treatments. Dashed outlines indicate lung and liver metastases in the H&E staining images. Scale bars: 100 μm. **m** The changes of body weight and (**n**) survival curves of 4T1 tumor-bearing mice following different treatments (*n* = 5 mice). **o** Rechallenged tumor growth curve and (**p**) survival rate of mice in the “BC@Z-M + L”-treated or control group after 4T1 cancer cells rechallenge (*n* = 5 mice). All data are presented as mean ± SD. For (**f**, **h**, **k**, and **l**), experiment was repeated three times independently with similar results. For (**c**, **e**, **g**, and **j**), statistical significance was determined using one-way ANOVA; for (**o**), statistical significance was determined using two-tailed Student’s *t*-test. Source data are provided as a Source Data file.

We then next evaluated the immunotherapeutic properties of “BC@Z-M + L” in 4T1 tumor-bearing mice. The 4T1 tumor models were established by subcutaneously inoculating 4T1 cells (10^6^ cells) into the right flank of female BALB/c mice on day −7 (Fig. 7a). On day 0, the mice with tumor volume of approximately 100 mm^3^ were randomly divided into six groups (*n* = 5 mice per group) and subjected to various treatments, including PBS, L, C@Z, BC@Z, BC@Z + L, and BC@Z-M + L. Twenty-four hours after intravenous injection of various formulations (200 μL, 1 mg mL^−1^ based on BN-O), the tumor sites in the groups labeled with “L” were irradiated with 730 nm laser (1.0 W cm^−2^) for 6 min. The PTT effect was initially evaluated by real-time monitoring of temperature using an infrared thermal camera. As depicted in Fig. 7b and Supplementary Fig. 51, after 6 min of 730 nm laser irradiation, the tumor temperature in the BC@Z-M-treated mice experienced a rapid increase from 37.3 °C to 55.2 °C. In contrast, the temperature elevations in the PBS and non-targeted BC@Z groups were 3.5 °C and 11.5 °C, respectively. The treatment of the primary tumor was conducted every 3 days for a total of 3 times. Following the tumor treatment, 4T1 cells were intravenously injected on day 5 to simulate malignant tumor invasion. The additional intravenous injection of 4T1 tumor cells into tumor-bearing mice is commonly employed to replicate hematogenous metastasis. These circulating cancer cells can invade various organs, with a particular propensity for the lungs. In comparison with spontaneous lung metastasis, the whole-body metastasis model is more aggressive and challenging, rendering it suitable for specialized anti-metastasis evaluations. The mice’s body weights and tumor volumes were meticulously recorded every two days.

As indicated in Fig. 7c, the tumors in PBS and L groups exhibited rapid growth throughout the treatment period, reaching average volumes of 1222 mm^3^ and 1149 mm^3^ on day 16, respectively. These results indicated that the NIR laser alone had minimal antitumor efficacy. However, the tumor volumes in the C@Z and BC@Z groups demonstrated moderate inhibition, with average tumor volumes of 754 mm^3^ and 746 mm^3^, respectively. This suggested that the vascular disrupting agent alone yielded certain but unsatisfactory tumor inhibition. Excitingly, the BC@Z-M + L treatment significantly curtailed tumor growth (average tumor volume = 123 mm^3^), with one out of five mice exhibiting complete regression of cancer (Fig. 7d). When compared to the BC@Z-M + L group, the “BC@Z + L” treatment had reduced anti-tumor performance due to its ineffective tumor accumulation. The mean tumor weight in the BC@Z-M + L group on day 16 was approximately 16.8-, 15-, 10.8-, 10.5- and 5.2-fold smaller than those observed in the PBS, L, C@Z, BC@Z, and BC@Z + L groups, respectively (Fig. 7e). The vascular disrupting agent could induce intratumoral coagulation and exacerbate tumor hypoxia, which, in turn, promoted the activation of the PDT and PTT effects of BC@Z-M within the tumor. Thus, the combination of hypoxia-activated phototherapy with vascular disrupting-based TME remodeling therapy demonstrated a self-amplified synergistic effect. The tumor blood vessels were stained with CD31 (Fig. 7f, g), revealing a significant reduction in the quantity of blood vessels following CA4P-containing NPs treatment. Hypoxia staining of tumor sections further confirmed increased hypoxia in tumors after CA4P treatment (Supplementary Fig. 52). These results validated the ability of CA4P-NPs to induce tumor coagulation and hypoxia. The ROS levels in tumor tissues were further assessed using DCFH-DA staining. CLSM images revealed intense ROS production in the tumor slides from both “BC@Z + L” and “BC@Z-M + L”-treated mice, with more pronounced ROS generation elicited by the “BC@Z-M + L” intervention (Supplementary Fig. 53). To evaluate the extent of apoptosis and necrosis in tumor tissues following various treatments, terminal deoxynucleotidyl transferase (TdT)-mediated dUTP-biotin nick end labeling (TUNEL) staining was conducted. The results demonstrated the highest green fluorescence with positive TUNEL staining in the “BC@Z-M + L” group, indicating the most significant extent of apoptosis and necrosis of tumor cells compared to other groups (Fig. 7h). These results were consistent with the superior tumor inhibition effect of “BC@Z-M + L” treatment observed earlier.

We next collected the lungs from each group to evaluate the degree of metastasis using india ink staining. As depicted in the photographs of harvested lung tissues and H&E staining (Fig. 7i–k), the PBS and single light irradiation groups exhibited severe lung metastases with a high number of lung nodules. In contrast, the “BC@Z-M + L” treatment showed the fewest appearance of tumor foci in the lungs. Similarly, the livers from “BC@Z-M + L”-treated mice also displayed the least metastatic tumor (Fig. 7l). These results indicated that the “BC@Z-M + L” intervention not only notably delayed the growth of primary tumor but also efficiently suppressed the progression of tumor metastasis. Moreover, there was negligible difference in the body weight of the mice subjected to various treatments, indicating the absence of severe systemic toxicity associated with all treatments (Fig. 7m). Additionally, “BC@Z-M + L” treatment significantly increased mice survival, with all mice still surviving on day 40. In contrast, the mice exposed to PBS or light irradiation alone all perished within 26 days (Fig. 7n). To ascertain whether the “BC@Z-M + L” treatment could provoke a long-term specific antitumor immune response, mice that survived in the “BC@Z-M + L” group were rechallenged with live 4T1 cells again on day 40 via subcutaneous injection into the left flank. The untreated naive mice were set as control and inoculated with the same dose of 4T1 cells. It was found that mice in the control group exhibited rapid tumor growth, and all animals died within 31 days post-tumor rechallenge (Fig. 7o, p). In contrast, the cured mice in the “BC@Z-M + L” group demonstrated effective resistance to tumor re-challenge, and they had a dramatically prolonged lifespan, displaying a high survival rate (80%) over the 40-day observation period. These results confirmed that the “BC@Z-M + L” treatment could elicit sustained anti-tumor immunity to prevent cancer recurrence, ultimately leading to the prolonged survival of tumor-bearing mice.

### Activation of systemic antitumor immunity

**Fig. 8:**
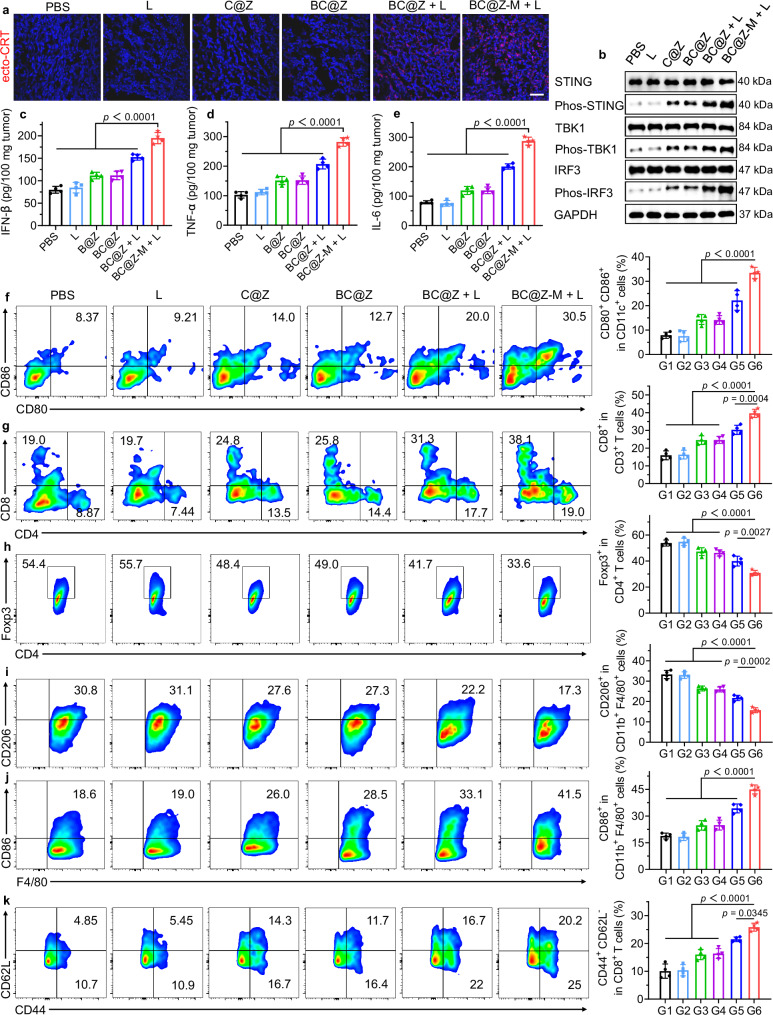
Antitumor immunity analyses after different treatments. **a** Representative CLSM images showing the expression of ecto-CRT (red fluorescence) on tumor sections after different treatments. The cell nuclei were stained with DAPI (blue fluorescence). Scale bar: 100 μm. **b** Western blotting analysis of the expression levels of STING pathway-related proteins in the tumor tissue. The expression levels of (**c**) IFN-β, (**d**) TNF-α, and (**e**) IL-6 in tumor tissue in different groups (*n* = 4 mice). **f** Representative flow cytometric analysis and quantitative data of mature DCs (CD11c^+^CD80^+^CD86^+^) in TDLNs of 4T1 tumor-bearing mice after different treatments (*n* = 4 mice). **g** Representative flow cytometric analysis and quantitative data of tumor-infiltrating CD8^+^ in CD3^+^ T cells in mice after different treatments (*n* = 4 mice). **h** Representative flow cytometric analysis and quantitative data of tumor-infiltrating CD4^+^Foxp3^+^ Tregs in different groups (*n* = 4 mice). Representative flow cytometric analysis and quantitative data of (**i**) tumor-infiltrating M2-like macrophages (CD206^+^CD11b^+^F4/80^+^) and (**j**) M1-like macrophages (CD86^+^CD11b^+^F4/80^+^) in 4T1 tumor-bearing mice after different treatments (*n* = 4 mice). **k** Representative flow cytometric analysis and quantitative data of CD3^+^CD8^+^CD62L^−^CD44^+^ T_EM_ in spleen of 4T1 tumor-bearing mice after different treatments (*n* = 4 mice). G1: PBS, G2: L, G3: C@Z, G4: BC@Z, G5: BC@Z + L, G6: BC@Z-M + L. All data are represented as mean ± SD. For (**a** and **b**), experiment was repeated three times independently with similar results. Statistical significance was determined using one-way ANOVA. Source data are provided as a Source Data file.

We next conducted comprehensive immunological analyses to unravel the underlying mechanisms responsible for the potent tumor inhibition effects of “BC@Z-M + L”. According to the findings from in vitro cell experiments, BC@Z-M plus light treatment could effectively trigger the ICD and STING pathways within tumor cells, promoting the release of DAMPs and proinflammatory cytokines and the maturation of DCs. The ecto-CRT expression on tumor tissues was first evaluated by immunofluorescence staining. As shown in Fig. 8a, tumor tissue in the “BC@Z-M + L” group exhibited the highest level of ecto-CRT expression, indicating efficient induction of ICD in vivo. We further determined the cGAS-STING pathway-related protein expression and cytokine secretion in tumor tissue through western blotting assay and ELISA. Compared to other groups, the expression levels of P-STING, P-TBK1, and P-IRF3 in the tumor tissue of BC@Z-M + L group were obviously increased (Fig. 8b). Meanwhile, the levels of STING-related cytokines, such as IFN-β, along with pro-inflammatory cytokines, including TNF-α and IL-6, were all notably elevated in “BC@Z-M + L” group (Fig. 8c–e). These observations suggested that the hypoxia-driven phototherapy, coupled with the release of Zn^2+^ from the nanocarrier, augmented the activation of STING pathway and effectively converted the “cold” tumor into more immunologically active “hot” tumor. The tumor antigens and proinflammatory cytokines released from tumor cells were expected to contribute to DCs maturation, which, in turn, would facilitate the subsequent activation of antitumor T cells and immune responses. To validate this hypothesis, tumor-draining lymph nodes (TDLNs), spleen, and tumor tissues from each group of mice were harvested for flow cytometry analysis of immune cell populations. As depicted in Fig. 8f and Supplementary Fig. 54, the “BC@Z-M + L” group exhibited a notably elevated proportion of mature DCs (CD11c^+^CD80^+^CD86^+^, 33.5%) in TDLNs, which was 4.24-, 4.44-, 2.33-, 2.37-, and 1.51-fold higher than those in PBS, L, C@Z, BC@Z, and BC@Z + L groups, respectively. This observation substantiated that “BC@Z-M + L” treatment effectively facilitated the maturation of DCs. As a crucial link in immune response, mature DCs can subsequently present tumor antigens to T lymphocytes, promoting their proliferation and activation, ultimately initiating anti-tumor immune-killing effects. Known as cytotoxic T lymphocytes (CTLs), CD8^+^ T cells are the most powerful effectors in the anticancer immune response. Encouragingly, the frequency of tumor-infiltrating CD8^+^ T cells (CD3^+^CD4^−^CD8^+^) in the “BC@Z-M + L” group was 2.48 times higher than that in the PBS group (Fig. 8g and Supplementary Fig. 55). The CD8^+^ T cell proportions in L, C@Z, BC@Z, and BC@Z + L groups were only 1.03- to 1.90-fold higher than that of PBS group. Likewise, the populations of tumor-infiltrating CD4^+^ T cells (CD3^+^CD4^+^CD8^−^), crucial for regulating adaptive immunities, were also significantly elevated in “BC@Z-M + L” group compared to other groups (Supplementary Fig. 56). To further confirm the pivotal roles of T lymphocytes in the observed antitumor effect, we conducted CD4/CD8 T cell depletion experiments by intraperitoneally administering anti-CD8 and anti-CD4 antibodies to mice. The therapeutic efficacy of “BC@Z-M + L” in tumor suppression was significantly impaired following the depletion of these T cell subsets (Supplementary Fig. 57), highlighting the essential roles of CD4/CD8 T cells in mediating the in vivo anti-tumor immune response. We also analyzed the intratumoral infiltration of regulatory T cells (Tregs, CD3^+^CD4^+^ Foxp3^+^), given Tregs’ role in restraining anti-tumor immune responses of CTLs. Evidently, the frequency of Tregs in “BC@Z-M + L” group was considerably lower compared to other groups (Fig. 8h and Supplementary Fig. 58), affirming that “BC@Z-M + L” effectively mitigated tumor-associated immunosuppression.

Tumor-associated macrophages (TAMs) constitute the most abundant population of tumor-infiltrating immune cells in TME, and their presence is strongly associated with unfavorable prognoses and resistance to therapy^61,62^. TAMs are primarily classified into two distinct phenotypes: M1 subtype with anti-tumor properties, and M2 subtype, characterized by pro-tumor characteristics^63^. To elucidate the impact of various treatments on macrophage polarization, we utilized flow cytometry to categorize the macrophages in tumor site. It was observed that the “BC@Z-M + L” treatment significantly reduced the populations of anti-inflammatory and immunosuppressive M2-like TAMs (CD206^+^CD11b^+^ F4/80^+^), while concurrently increasing the proportion of proinflammatory M1-like TAMs (CD86^+^CD11b^+^ F4/80^+^), resulting in the highest M1/M2 ratio among all the groups (Fig. 8i, j and Supplementary Figs. 59, 60). These findings indicated the capability of “BC@Z-M + L” treatment to effectively shift pro-tumoral M2-like macrophages into anti-tumoral M1-like macrophages. Furthermore, we analyzed the levels of CD8^+^ effector memory T cells (T_EM_, CD3^+^CD8^+^CD44^+^CD62L^−^) in the spleens of mice to assess the establishment of immune memory effects. As depicted in Fig. 8k and Supplementary Fig. 61, the percentage of T_EM_ in the “BC@Z-M + L” group was significantly elevated, being 2.57-, 2.49-, 1.61-, 1.58-, and 1.20-fold higher than that in the PBS, L, C@Z, BC@Z, and BC@Z + L groups, respectively. These results affirmed that the combination of vascular disruption with hypoxia-activated photoimmunotherapy efficiently stimulates the body’s anti-tumor immune response, leading to robust suppression of tumor growth.

In addition to flow cytometry analysis, we performed transcriptomic analyses of tumor tissue to assess the types of tumor-infiltrating immune cells before and after treatment. The results revealed substantial alterations in the immune landscape following “BC@Z-M + L” treatment (Supplementary Fig. 62). Specifically, there was an increased infiltration of CD4^+^ and CD8^+^ T cells in tumor tissue, indicating enhanced anti-tumor immune activation. Additionally, we observed elevated abundances of tumor-infiltrating B cells, dendritic cells, macrophages, and natural killer (NK) cells while reduced levels of immunosuppressive Treg cells in tumor deposits receiving “BC@Z-M + L” treatment. These findings were consistent with our previous flow cytometry analysis, confirming the role of our treatment in modulating the tumor immune microenvironment and potentiating the anti-tumor immune response. Furthermore, we observed the presence of tertiary lymphoid structures within the tumor from mice treated with “BC@Z-M + L”, characterized by a distinct CD20^+^ B cell adjacent to a CD4^+^ and CD8^+^ T cell zone (Supplementary Fig. 63). As tertiary lymphoid structures provide a structured microenvironment for the activation, expansion, and maturation of immune cells, their formation at tumor site can potentially revitalize local anti-tumor immunity and enhance the efficacy of immunotherapeutic treatments.

### Tumor-suppressive effect of BC@Z-M in bilateral tumor model

**Fig. 9:**
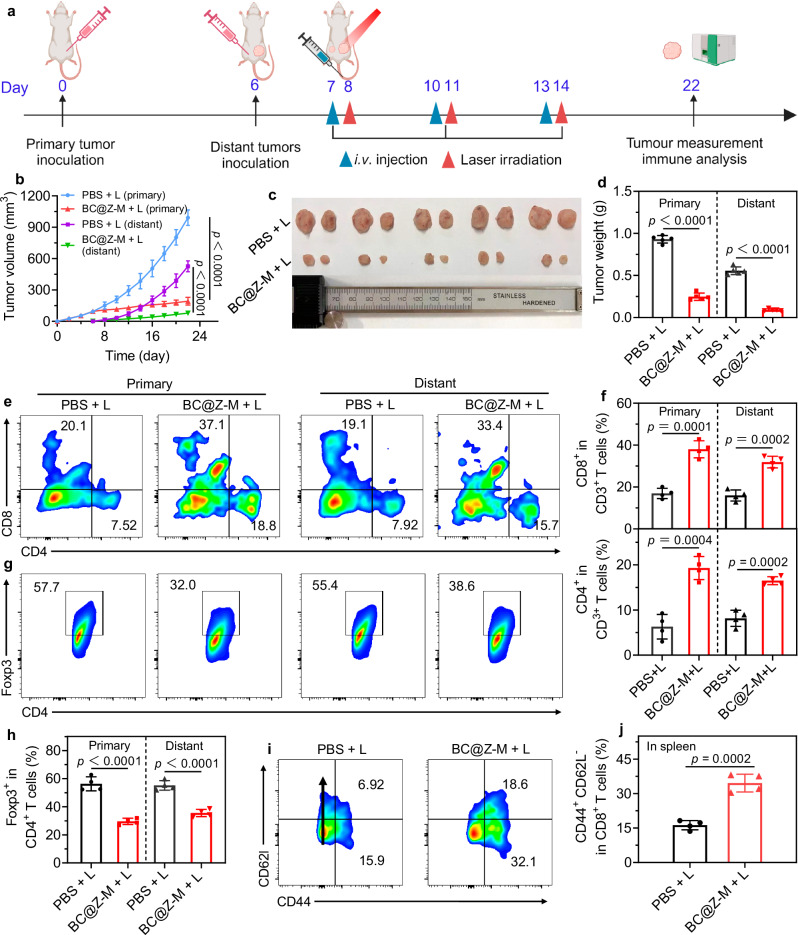
Tumor suppression and immune activation in bilateral tumor model. **a** Schematic illustration of the treatment procedure and assessment of therapeutic efficacy of BC@Z-M in bilateral tumor model. Created in BioRender. Li, W. (2024) BioRender.com/v06y872. **b** Tumor growth curve and (**d**) tumor weights in “PBS + L” or “BC@Z-M + L” group (*n* = 5 mice). **c** Photographs of the tumors resected from bilateral tumor-bearing mice after the treatment of “PBS + L” or “BC@Z-M + L” (*n* = 5 mice). **e** Representative flow cytometric analysis and (**f**) quantitative data of tumor-infiltrating CD8^+^CD3^+^ T and CD4^+^CD3^+^ T cells in primary and distant tumors from bilateral 4T1 tumor-bearing mice after “PBS + L” or “BC@Z-M + L” treatments (*n* = 4 mice). **g** Representative flow cytometric analysis and (**h**) quantitative data of tumor-infiltrating CD4^+^Foxp3^+^ Tregs in primary and distant tumors collected from bilateral 4T1 tumor-bearing mice after “PBS + L” or “BC@Z-M + L” treatments (*n* = 4 mice). **i** Representative flow cytometric analysis and (**j**) quantitative data of CD3^+^CD8^+^CD62L^−^CD44^+^ T_EM_ in spleen collected from bilateral 4T1 tumor-bearing mice in “PBS + L” or “BC@Z-M + L” group (*n* = 4 mice). All data are represented as mean ± SD. Statistical significance was determined using two-tailed Student’s *t*-test. Source data are provided as a Source Data file.

We subsequently established a bilateral tumor model to investigate the abscopal inhibition effect of combination therapy on untreated distant tumors. As depicted in Fig. 9a, the bilateral tumor model was built by injecting 4T1 cells (10^6^ cells) into the right flank of BALB/c mice as primary tumors on day 0. Subsequently, 5 × 10^5^ of 4T1 cells were implanted into the left flanks of the mice on day 6 to simulate distant tumors. On day 7, when the primary tumor reached ~100 mm^3^, the mice were randomly assigned into two groups (*n* = 5 mice per group). For each group, either PBS or BC@Z-M was intravenously injected into mice every 3 days for a total of 3 times. At 24 h post-injection, only the primary tumor was irradiated with 730 nm laser (1.0 W cm^−2^), while the distant tumor was left untreated. The progression of tumors on both sides was monitored every two days to assess the therapeutic effect. Interestingly, when compared to the PBS + L group, the treatment with “BC@Z-M + L” not only notably slowed down the growth of primary tumors with an 81% inhibition rate but also efficiently suppressed distant tumors (85% inhibition rate) that were not exposed to light (Fig. 9b–d).

We subsequently analyzed the immune status of bilateral tumors to unveil the mechanism behind the tumor inhibition in the “BC@Z-M + L” group. As effector CD8^+^ T cells can directly eliminate cancer cells, we first focused on the infiltration of CD8^+^ T cells in both primary and distal tumors. As depicted in Fig. 9e, f, the “BC@Z-M + L” group exhibited increased CD8^+^ T cell infiltration within both primary and distal tumors, with 2.25- and 2.00-times higher than those in the PBS + L group. Likewise, the proportions of CD4^+^ T cells in both primary and distal tumor were markedly elevated in the mice treated with BC@Z-M + L, in contrast to the PBS + L control group. Furthermore, the “BC@Z-M + L” treatment led to a notable decrease in the percentage of regulatory T cells (Tregs) in both primary and distal tumors (Fig. 9g, h). Beyond the immediate anti-tumor immune response, the establishment of immune memory plays a crucial role in preventing tumor recurrence. Therefore, we evaluated T_EM_ in the spleen of mice. The population of T_EM_ in the “BC@Z-M + L” group was 2.13-times higher than in the “PBS + L” group (Fig. 9i, j). These findings suggested that vascular disruptor-mediated tumor remodeling, combined with the amplified hypoxia-boosted PDT/PTT, could effectively enhance the anti-tumor immune response.

The biocompatibility of BC@Z-M was finally assessed by histological analysis of vital organs, blood biochemistry, and routine blood tests. Healthy mice were divided into two groups (*n* = 3 mice per group) and intravenously injected with PBS or BC@Z-M every 3 days for a total of 3 times. First, there were no significant differences in weight trends between the mice injected with PBS and those injected with BC@Z-M (Supplementary Fig. 64). Major organs including the heart, liver, spleen, lung, and kidneys were collected from each group on day 14 for histological analysis. At the same time, blood was also collected for blood biochemistry and routine blood index analyses. As depicted in Supplementary Fig. 65, the major organs from mice treated with BC@Z-M revealed negligible histological abnormalities and tissue damage. Additionally, akin to the PBS group, various blood parameters of the BC@Z-M-injected group all remained within the normal range (Supplementary Fig. 66). And no significant differences were observed in serum biochemistry parameters related to liver and kidney function between the PBS group and NP group (Supplementary Fig. 67), suggesting the absence of acute liver or kidney injury. These results indicated that BC@Z-M exhibited good biosafety in vivo.

## Discussion

In this work, we have developed a versatile nanoplatform that integrated hypoxia-activatable multifunctional molecular imaging and combination immunotherapy for sensitive diagnosis and potent treatment of cancer. While the *N*-oxide motif has been studied for hypoxia response, such as the fluorescence probe for in vitro hypoxic cell imaging and hypoxia-responsive drug delivery^64,65^, our work introduced a molecular probe with distinct properties and functionalities not reported in existing systems. The developed probe demonstrated turn-on NIR-II fluorescence and PA imaging capabilities upon exposure to hypoxic stimuli, coupled with hypoxia-triggered photodynamic and photothermal therapeutic effects. The synergistic activation of both imaging and therapeutic capabilities under hypoxic conditions made BN-O not only promising for hypoxic tumor diagnosis but also proficient in on-demand photoimmunotherapy. NIR-II fluorescence and PA imaging are both advanced optical imaging techniques that offer complementary benefits. By combining them, we leverage the strengths of each modality to achieve a comprehensive tumor assessment. Specifically, NIR-II fluorescence imaging provides precise tumor localization with high signal-to-noise ratio, while PA imaging offers valuable details on tumor size, morphology, and distribution pattern due to its superior penetration depth and spatial resolution. Currently, the NIR-II fluorescence and PA imaging are conducted with separate imaging systems, and the future developments in optical imaging equipment capable of simultaneously capturing both signals will further expand the utility and impact of such advanced imaging probes for comprehensive tumor characterization.

Beyond high-contrast in vivo hypoxic tumor imaging, the hypoxia-triggered PTT/PDT attributes elicited by the probe jointly activated ICD and the cGAS-STING pathway to enhance tumor-specific immune response and mitigate immunological resistance. Although PTT and PDT have been employed for tumor immunotherapy, our platform addresses several critical challenges faced by current systems. Firstly, unlike traditional “always-on” photosensitizers, our molecular probe exhibits hypoxia-triggered PDT and PTT effects, enabling precise, on-demand photoimmunotherapy activation. Moreover, the PDT process activated by our probe primarily operates via a less oxygen-dependent type-I mechanism, making it particularly advantageous for treating hypoxic tumors. We demonstrated that the concurrent activation of robust PDT and PTT effects could boost antitumoral immunity by activating ICD and STING pathways, effectively engaging both innate and adaptive immune responses. Moreover, we further augmented the hypoxia-activated molecular probe with a tumor-targeting and TME-remodeling nanocarrier to amplify the hypoxia-responsive phototheranostics. The co-encapsulated vascular disrupting agent-induced nutrient deprivation in tumor cells and intensified the hypoxic TME, therefore accelerating the hypoxia-triggered phototherapy. In tumor-bearing mice model, the hypoxia-triggered and TME remodeling-boosted photoimmunotherapy not only impeded the growth of primary tumor but also hindered the progression of distant tumor, while also conferring protection against subsequent tumor rechallenge and the onset of pulmonary metastasis. This work provides a perspective for advancing high-performing and activatable theranostic protocols for precise image-guided tumor immunotherapy.

## Methods

### Materials

4-Bromo-*N*,*N*-diethylaniline was obtained from Heowns Co., Ltd (Tianjin, China). 4,7-Dibromobenzo[1,2-*c*:4,5-*c*’]bis([1,2,5]thiadiazole) was purchased from alfachem Co., Ltd (Zhengzhou, China). 3-Chloroperbenzoic acid was obtained from Innochem Co., Ltd (Beijing, China). Tetrakis(triphenylphosphine)palladium was obtained from J&K Scientific Co., Ltd (Shanghai, China). Methylimidazole (2-MI) and zinc nitrate (Zn(NO_3_)_2_•6H_2_O) were purchased from Aladdin Biochemical Technology Co., Ltd (Shanghai, China). Combretastatin-A4 phosphate (CA4P) was purchased from Hangzhou Great Forest Biomedical Ltd., China. All chemicals are of analytical grade standards and do not require further purification.

Lipopolysaccharide (LPS) and interferon-γ (IFN-γ) were acquired from eBioscience. Roswell Park Memorial Institute (RPMI) 1640 culture medium, penicillin-streptomycin, and fetal bovine serum (FBS) were sourced from Gibco-BRL (Grand Island, NY, USA). Cell culture dishes and confocal dishes were procured from Corning Incorporated Co., Ltd (Beijing, China). 3-(4,5-Dimethylthiazol-2-yl)-2,5-diphenyltetrazolium bromide (MTT) was provided by Saiguo Biotech Co., Ltd (BioFROXX, Germany). Reactive oxygen species assay kit (DCFH-DA and HPF), TUNEL apoptosis assay kit, DNA damage detection kits, and mitochondrial membrane potential assay kit (JC-1) were purchased from Beyotime Biotechnology (Shanghai, China). IL-6, TNF-α, and IFN-β ELISA kits were obtained from Dakewe Co., Ltd (Beijing, China). CFSE cell division tracker kit was provided by Dakewe Biotech Co., Ltd (Biolegend, America). Antibodies used in this study include: Western blotting: anti-Na,K-ATPase (Cell Signaling Technology, 3010, 1:1000), anti-CD86 (Cell Signaling Technology, 19589, 1:1000), Recombinant anti-iNOS (Abcam, ab178945, 1:1000), anti-CD47 (ABclonal, A1838, 1:1000), anti-Integrin alpha 4/CD49D (Abcam, ab75760, 1:1000), anti-Integrin beta 1 (Abcam, ab179471, 1:1000), GADPH (Proteintech, 10494-1-AP, 1:10,000), HRP-conjugated anti-rabbit secondary antibody (Proteintech, SA00001-2, 1:5000), anti-STING (Abcam, ab288157, 1:1000), anti-p-STING (Cell Signaling Technology, 72971, 1:1000), anti-TBK1 (Abcam, ab40676, 1:1000), anti-p-TBK1 (Cell Signaling Technology, 5483, 1:1000), anti-IRF3 (Abcam, ab68481, 1:1000), anti-p-IRF3 (Cell Signaling Technology, 29047, 1:1000), anti-MDA-5 (Cell Signaling Technology, 5321, 1:1000), anti-RIG-I (Cell Signaling Technology, 3743, 1:1000), anti-AIM2 (Absin, abs125828, 1:1000), anti-β-actin (Cell Signaling Technology, 4967S, 1:1000). Antibodies for western blot analyses were diluted in Primary Antibody Dilution Buffer. Fluorescent antibodies used in this study include: Immunofluorescence analysis: Recombinant AntiCD31 antibody (Abcam, ab222783, 1:200); Anti-HIF-1α Antibody (Cohesion, CPA3239, 1:200); Recombinant Anti-HMGB1 antibody (Abcam, ab79823, 1:200), Recombinant Anti-Calreticulin antibody (Abcam, ab92516, 1:200); Goat Anti-Rabbit IgG H&L (Alexa Fluor® 488) (Abcam, ab150077, 1:1000); Donkey Anti-Rabbit IgG H&L (Alexa Fluor® 647) (Abcam, ab150075, 1:1000). Flow cytometry: anti-CD11c-FITC (Biolegend, 117306, 1:200); anti-CD80-PE (Biolegend, 104707, 1:200); anti-CD86-APC (Biolegend, 105011, 1:200); anti-CD3-FITC (Biolegend, 100203, 1:200); anti-CD4-BV421 (Biolegend, 100543, 1:200); anti-CD8a-BV421 (Biolegend, 100711, 1:200); anti-Foxp3-PE (Biolegend, 320007, 1:200); anti-F4/80-PE (Biolegend, 123109, 1:200), anti-CD11b-FITC (Biolegend, 101205, 1:200); anti-CD206-BV421 (Biolegend, 141717, 1:200); anti-CD44-PE (Biolegend, 103007, 1:200); anti-CD62L-BV605 (Biolegend, 104437, 1:200); anti-CD3-APC/Cyanine7 (Biolegend, 100221, 1:200); anti-H-2Kb bound to SIINFEKL-PE/Cyanine7 (Biolegend, 141607, 1:200); anti-IFN-γ-PE (Biolegend, 505808, 1:200). Hematoxylin-eosin staining solution (alcohol soluble) was procured from Leagene Biotechnology Co., Ltd (Beijing, China).

### Cell lines and animals

The RAW264.7 murine macrophage cell line and 4T1 breast cancer cell line were purchased from the Chinese Academy of Sciences Cells Bank (Shanghai, China). The 4T1 cell line was maintained in RPMI-1640 medium supplemented with penicillin (100 U mL^−1^), streptomycin (100 μg mL^−1^), and 10% fetal bovine serum (FBS). RAW264.7 cells were cultured in the complete DMEM medium.

BALB/c mice (female, 6–7 weeks old) were purchased from the Laboratory Animal Center of the Academy of Military Medical Sciences (Beijing, China). The animals were housed in a controlled environment with a temperature of ~25 °C and a 12 h light/dark cycle, suitable humidity (typically 50%), ensuring free access to standard food and water. Humane endpoints were established, including tumor burden exceeding 10% of normal body weight, animal weight loss surpassing 20% of normal weight, the presence of ulcers at tumor growth sites, and sustained self-mutilation. Approval for these humane endpoints was granted by the Certification and Accreditation Administration of the People’s Republic of China (CNCA). All procedures involving animals were conducted by the guidelines set by the Tianjin Committee of Use and Care of Laboratory Animals, and approved by the Animal Ethics Committee of Nankai University (2021-SYDWLL-000380).

### Characterizations

^1^H and ^13^C nuclear magnetic resonance (NMR) spectra were acquired using a Bruker-DPX 400 spectrometer. High-resolution mass spectra (HRMS) were performed on a Bruker AutoflexIII LRF200-CID mass spectrometer in matrix-assisted laser desorption/ionization time-of-flight (MALDI-TOF) mode. The absorption spectra were detected using a Shimadzu UV-1800 spectrometer. Edinburgh FS5 was used to measure the photoluminescence (PL) spectra. Dynamic light scattering (DLS) measurements were performed on a Malvern Zeta sizer Nano ZS-90. Transmission electron microscope (TEM) images were captured using a Talos L120C G2 instrument by FEI, Czech. Molecular geometry optimization was conducted using the DFT method implemented in the Gaussian 09 program package (revision D. 01), with Cartesian coordinates provided in Supplementary Tables 1 and 2. The crystal structure characterization was carried out on XRD (D8 Advance, Bruker, Germany). PA imaging was conducted on a commercial small-animal optoacoustic tomography system (Vevo LAZR, FujiFilm VisualSonics, America). An in vivo imaging system (IVIS, NightOWL II LB983) and short-wavelength infrared (SWIR) imaging system (Wuhan Grand-imaging Technology Co., LTD) were respectively utilized for NIR-I and NIR-II fluorescence imaging of animals and organs.

### Hypoxia-responsive property of BN-O

For the hypoxia-responsive assessment, BN-O was preincubated with rat liver microsomes for 5 min at 37 °C in nitrogen ambient atmosphere. Subsequently, NADPH was added and the samples were taken at fixed intervals for monitoring the absorption and fluorescence spectra. Spectral parameters were as follows: absorbance spectra ranged from 450 nm to 900 nm; NIR-I FL: λex = 550 nm, emission range = 570–750 nm; NIR-II FL: λex = 730 nm, emission range = 900–1400 nm. To evaluate the PA performance in solution, the PA intensity of different concentrations of BN and BN-O solution was recorded using a Vevo® LAZR-X system. The parameters for in vitro PA measurement were set as follows: frequency = 30 MHz, wavelength range = 680–970 nm, PA gain = 40 dB, depth/width = 12.00/14.08 mm, pulse repetition rate = 20 Hz, duration of pulsed laser = 7 ns. Additionally, the PA signal of BN-O solution before and after hypoxia simulation was measured using the same parameters.

### Preparation of M1-like macrophage membrane and fabrication of BC@Z-M

First, RAW264.7 cells were stimulated with LPS and IFN-γ for 24 h to induce polarization towards M1-like macrophages. Subsequently, M1-polarized cells underwent three washes with phosphate-buffered saline (PBS), after which they were harvested and subjected to hypotonic lysis at 4 °C overnight. The cells were then mechanically disrupted using a grinding stick and centrifuged at 3000 × *g* for 10 min to obtain the supernatant. The collected supernatant was further centrifuged at 12,000× g × 10 min, followed by a final centrifugation at 100,000 × *g* for 1 h to isolate the membrane precipitate. Prior to storage, the protein concentration was quantified at 1 mg mL^−1^ (derived from 10^9^ cells, 500 μL) using the BCA protein assay kit.

BC@Z-M was then prepared as the following procedures. In detail, 10 mg of Zn(NO_3_)_2_•6H_2_O and 20 mg CA4P were dissolved in 5 mL of deionized water and then added dropwise under magnetic stirring to 5 mL of deionized water solution containing 400 mg of 2-MI and 20 mg of BN-O. The resulting mixture was stirred for 10 min, after which the product was collected by centrifugation (2400 × *g*) and washed three times with deionized water. The encapsulation efficiencies of CA4P and BN-O were determined to be 22.05% and 10.9%, respectively. The resulting BC@Z (100 µL) was then combined with 0.1 mg of freshly isolated M1-like macrophage membranes, which subsequently underwent sonication and extrusion through a 400 nm polycarbonate filter using an Avanti Mini extruder for 20 repeated cycles. Subsequently, the polycarbonate filter was replaced with filter with a 220 nm pore size and extruded 20 times consecutively. Finally, BC@Z-M was obtained after centrifugation at 2400 × *g* for 15 min.

The proteins extracted from macrophage membranes and BC@Z-M were then subjected to western blotting analysis. BC@Z without membrane coating served as the control group. M1-like macrophage membranes and different nanoformulations were lysed using RIPA buffer, and their total protein concentrations were determined using BCA protein assay. After mixing with loading buffer, the protein samples were denatured at 100 °C for 10 min. Following this, 20 μg of protein from each sample was subjected to SDS-PAGE electrophoresis and transferred onto a polyvinylidene fluoride (PVDF) membrane. The PVDF membrane was then blocked and incubated with primary antibodies (anti-iNOS, anti-CD86, anti-integrin α1, and anti-integrin β1; 1:1000 dilution, Abcam) at 4 °C overnight. Following primary antibody incubation, the PVDF membrane was further incubated with the HRP-conjugated secondary antibodies and the ECL chemiluminescence substrate kit (Servicebio), and observed with gel imaging system.

### pH-dependent drug release

For the assessment of drug release under varying pH conditions, BC@Z-M was dissolved in 1.0 mL of PBS (100 μg mL^−1^) and then transferred into dialysis bags. These bags were submerged in glass tubes containing 9.0 mL of solutions at different pH levels (pH 7.4 and pH 6.5). These glass tubes were placed within a darkened enclosure set at 37 °C with continuous shaking. At predetermined intervals, 1 mL of solution was withdrawn from each tube, and an equal volume of fresh solution at the corresponding pH was added to replenish each tube. The amount of CA4P released at each time point was quantified using an UV-Vis spectrophotometer. The analysis of the release of zinc ions from the nanocarrier at different pH values was conducted using ICP-MS.

### Cellular uptake

4T1 tumor cells and RAW264.7 cells were inoculated onto the confocal chambers and allowed to adhere overnight. The culture medium was then replaced with fresh medium containing BC@Z-M (20 µg mL^−1^ based on BN-O). After incubation, the cells were washed three times with PBS, fixed with 4% tissue fixative for 15 min, and then washed again three times with PBS. Subsequently, the cell nuclei were stained with DAPI for 10 min, followed by washing away of the residual dye with PBS. Finally, the cells were imaged using confocal laser scanning microscopy (CLSM) (LSM 800 with Airyscan, ZEISS, Germany) and analyzed using ZEN 2012 software.

### Hypoxia responsiveness of BC@Z-M in living cells

The hypoxic responsiveness of BC@Z-M in living cells was evaluated by incubating cells with BC@Z-M under either normoxic or hypoxic conditions. 4T1 tumor cells were cultured under normoxic conditions at 5% of CO_2_ and 20% of O_2_ at 37 °C. For anaerobic cell cultures, an anaerobic incubator chamber was utilized, filled with a gas mixture of 94% N_2_, 5% CO_2_, and 1% O_2_ (Tianjin Baiyao Gas Co., Ltd.). 4T1 mouse breast cancer cells were treated with BC@Z-M and maintained at 37 °C under either normoxic or hypoxic conditions. The intracellular NIR-I fluorescence (excitation at 530 nm, emission at 620 nm) or NIR-II fluorescence (excitation at 730 nm, emission at 1000 nm) signals from the nanoprobes were observed using CLSM at various time points. Additionally, 4T1 cells were cultured in 12-well plates and incubated with BC@Z-M (10 μM based on BN-O) under normoxic or hypoxic conditions for 3 h. Subsequently, the NIR-I and NIR-II fluorescence signals from the cell wells were monitored using an IVIS (NightOWL II LB983) and a short-wavelength infrared (SWIR) imaging system (Wuhan Grand-imaging Technology Co., LTD), respectively.

### Intracellular ROS detection

4T1 tumor cells were seeded in confocal chambers and allowed to adhere overnight. Following removal of the medium, fresh serum-free DMEM containing C@Z, BC@Z, or BC@Z-M (20 μg mL^−1^ based on BN-O) was added and co-incubated for 24 h at 37 °C under hypoxic conditions (anaerobic chamber, 1% O_2_). As a control, the cells treated with BC@Z-M under normoxic conditions (20% O_2_) were also included. Subsequently, the cells were washed three times with PBS and stained with 10 µM of 2’,7’-dichlorodihydrofluorescein diacetate (DCFH-DA) in the dark. After removing the free probe, the cells were irradiated with 730 nm laser (1.0 W cm^−2^) for 5 min and further incubated at 37 °C for another 30 min. For CLSM imaging, the excitation for dichlorofluorescein (DCF), generated by DCFH-DA reacting with ROS, was set at 488 nm with emission collected at 530 ± 20 nm.

### Cytotoxicity study

For the normoxic toxicity assessment, 4T1 cells were seeded in 96-well plates (5 × 10^3^ cells per well) and cultured overnight. The cells were then treated with varying concentrations of BC@Z-M for 24 h. Subsequently, the cells were washed thrice with PBS. For the dark toxicity assessment, 100 μL of MTT solution was directly added to each well. After 4 h, the MTT solution was aspirated, and 150 μL of DMSO was added to each well, followed by shaking at room temperature. The optical density (OD) values at 570 nm were finally measured by a microplate reader. In the phototoxicity group, the cells were exposed to 730 nm laser (1.0 W cm^−2^) for 5 min after 24 h of incubation with the NPs. After an additional 12 h incubation period, the MTT assay was conducted. For the toxicity evaluation of nanoformulations under anoxic conditions, the cells were cultured in a hypoxic atmosphere consisting of 94% N_2_, 5% CO_2_, and 1% O_2_. The subsequent steps were identical to those under normoxic conditions. Additionally, a calcein-AM/PI double staining kit was utilized to differentiate between dead and viable cells at 12 h after laser irradiation. The stained cells were finally observed using a fluorescence microscope. To evaluate cell apoptosis and death, the cells subjected to different treatments were stained using the Annexin V-FITC/PI kit (C1062S, Beyotime, China) following the manufacturer’s protocol, and subsequently analyzed by flow cytometry.

### Characterization of immunologic cell death in 4T1 cells

4T1 tumor cells were seeded at a density of 10^5^ cells per dish in confocal chambers and cultured overnight. Subsequently, the cells were incubated with C@Z, BC@Z, or BC@Z-M (20 μg mL^−1^ based on BN-O) in a hypoxic chamber for 24 h, followed by irradiation with 730 nm laser for 5 min. At 12 h post-irradiation, the cells were fixed with 4% paraformaldehyde for 15 min. DAMPs, including ecto-CRT, HMGB1, and ATP, were then analyzed for the characterization of ICD in tumor cells. For CRT and HMGB1 analyses, the cells were fixed, blocked, and incubated with rabbit primary antibodies (anti-CRT and anti-HMGB1) diluted 1:200 at 4 °C overnight. Subsequently, the cells were further incubated with secondary antibodies for 1 h in the dark. After three washes, the cells were stained with DAPI for 15 min, and the expression of CRT and HMGB1 was observed under CLSM. To detect the extracellular ATP, 4T1 tumor cells were seeded in 6-well plates at a density of 10^5^ cells per well. The cells were then incubated with C@Z, BC@Z, or BC@Z-M (20 μg mL^−1^ based on BN-O) in a hypoxic chamber for 24 h at 37 °C, followed by irradiation with a 730 nm laser (1.0 W cm^−2^) for 5 min. At 12 h post-photostimulation, the culture supernatant was centrifuged at 13,500 × *g* at 4 °C for 10 min. The level of secreted ATP in the supernatant was quantified using an ATP bioluminescence assay kit according to the manufacturer’s instructions. The levels of HMGB1 and CXCL10 in the cell supernatant were measured using HMGB1 ELISA kit (E-EL-M0676c, Elabscience) and CXCL10 ELISA kit (E-EL-M0021, Elabscience) according to the manufacturer’s instructions.

### Mitochondrial dysfunction and DNA breaks induced by nanoformulations

4T1 tumor cells, with a density of 10^5^ cells per dish, were cultured in confocal chambers and subjected to treatment with C@Z, BC@Z, or BC@Z-M (20 μg mL^−1^ based on BN-O) for 24 h under hypoxic conditions at 37 °C. Subsequently, the cells were subjected to the irradiation of 730 nm laser for 5 min. At 12 h post-irradiation, JC-1 probe, a sensitive indicator of mitochondrial membrane potential, was employed to detect mitochondrial function. Cellular mitochondria were stained with JC-1 at 37 °C for 30 min, and the changes in mitochondrial membrane potential were visualized using CLSM. The JC-1 aggregates, typically emitting red fluorescence around 590 nm, are commonly found in regions of the cell characterized by high mitochondrial membrane potential, whereas the JC-1 monomers, emitting green fluorescence around 529 nm, predominate in regions of the cell with low mitochondrial membrane potential. To assess DNA breaks, the cells were subjected to γ-H2AX immunofluorescence staining. Twelve hours after light irradiation, the cells were washed with PBS and then blocked with QuickBlock™ Blocking Buffer for 15 min. Subsequently, the cells were incubated at room temperature for 1 h with an anti-γ-H2AX primary antibody (rabbit monoclonal; Beyotime, Shanghai, China; diluted at 1:200). Following this, 4T1 cells were incubated with a fluorescein isothiocyanate (FITC)-conjugated secondary antibody (Beyotime, Shanghai, China; diluted at 1:500) for 1 h at room temperature, and the cell nuclei were stained with DAPI. The cells were imaged using CLSM to detect nuclear damage, with FITC excitation at 495 nm and emission at 519 ± 20 nm. For the detection of DNA oxidation, the pretreated cells were incubated with anti-8OHdG antibody (Santa Cruz Biotech, sc-393871, 1:50) to label the oxidized DNA following the manufacturer’s instructions. After washing with PBS, the cells were stained with DAPI to label nuclei and imaged using a confocal microscope.

### cGAS-STING pathway activation in 4T1 cancer cells

4T1 tumor cells were seeded into six-well plates and cultured overnight. The cells were then treated with PBS, C@Z, BC@Z, or BC@Z-M (20 μg mL^−1^ based on BN-O) for 24 h under hypoxic condition at 37 °C. Subsequently, the cells were exposed to 730 nm laser for 5 min. At 12 h post-irradiation, the cells were harvested and lysed for analysis of STING pathway-related protein expression using western blotting. The protein extracts were then heated in a boiling water bath for 10 min and subjected to SDS-PAGE electrophoresis, followed by transfer onto a PVDF membrane. After one-hour incubation in blocking solution, the PVDF membrane was then incubated overnight at 4 °C with a primary antibody solution containing anti-GAPDH (Proteintech, 10494-1-AP, 1:10,000), anti-STING (Abcam, ab288157, 1:1000), anti-p-STING (Cell Signaling Technology, 72971, 1:1000), anti-TBK1 (Abcam, ab40676, 1:1000), anti-p-TBK1 (Cell Signaling Technology, 5483, 1:1000), anti-IRF3 (Abcam, ab68481, 1:1000), and anti-p-IRF3 (Cell Signaling Technology, 29047, 1:1000). Following incubation, the PVDF membrane was washed with blotting buffer and then incubated with an HRP-conjugated anti-rabbit secondary antibody (Proteintech, SA00001-2, 1:5000) for 1 h at room temperature. After washing, the membrane was treated with chemiluminescent detection substrate and visualized using a gel imaging system. Simultaneously, the culture supernatants were collected for ELISA analysis to quantify the concentrations of TNF-α, IL-6, and IFN-β released by cells, following the manufacturer’s protocols. The STING-overexpressing 4T1 tumor cells were generated using a STING expression plasmid vector, while STING silencing was achieved with specific shRNAs. Silencing of AIM2, RIG-I, and MDA-5 in 4T1 tumor cells was accomplished using their respective shRNAs. The sequences for all shRNAs were provided in Supplementary Data 1.

### BMDC maturation

Bone marrow-derived dendritic cells (BMDCs) were isolated from the femurs and tibias of 6-8-week-old female BALB/c mice and induced to differentiate using BMDC medium, consisting of RPMI−1640 supplemented with 1% penicillin-streptomycin, 10% FBS, interleukin-4 (IL-4) (10 ng mL^−1^), and granulocyte-macrophage colony-stimulating factor (GM-CSF) (20 ng mL^−1^) for five days. For the in vitro BMDCs maturation study, 4T1, B16-F10 melanoma, or MC38 colon carcinoma cells were first seeded into the transwell chambers and allowed to attach overnight. Subsequently, the cells were treated with PBS, C@Z, BC@Z, or BC@Z-M (20 μg mL^‒1^ based on BN-O). After 24 h of incubation, the cells were subjected to 730 nm laser irradiation (1.0 W cm^−2^, 5 min). Following irradiation, the transwell chambers were transferred to the top layer of BMDC culture dishes and incubated for an additional 24 h. Finally, BMDCs from the bottom layer were harvested for flow cytometric analysis of the expression of CD11c, CD80, and CD86 to assess BMDCs maturation in each group. Additionally, culture supernatants were collected for ELISA analysis to measure the concentrations of TNF-α, IL-6, and IFN-β according to the manufacturer’s protocols.

### Antigen cross-presentation and antigen-specific T cell activation

The B16-OVA cells were treated with various formulations (20 μg mL^‒1^ based on BN-O), followed by light irradiation in the designated groups. The pretreated B16-OVA cells were then co-cultured with BMDCs in transwell chambers for 24 h. BMDCs from the bottom chamber were harvested to assess their maturation and surface H-2K^b^/SIINFEKL complex presentation via flow cytometry. To assess OVA-specific CD8^+^ T cell activation, T cells were isolated from the spleen of OT-1 mice and then co-cultured with pretreated BMDCs for 24 h. Subsequently, intracellular staining for IFN-γ was performed to analyze IFN-γ expression in CD8^+^ T cells. T cell proliferation was assessed using the CFSE cell division tracker kit (Biolegend, USA) according to the manufacturer’s protocol. To evaluate T cell-mediated tumor cell killing, OT-1-derived CD8^+^ T cells were co-incubated with pretreated BMDCs for 24 h to activate antigen-specific CD8^+^ T cells. B16-OVA cells were then introduced at an effector-to-target ratio of 10:1, and the culture was continued for an additional 4 h before conducting a LDH assay to assess tumor cell cytolysis.

### In vivo activatable NIR-II FL and PA imaging of tumors

To establish the 4T1 tumor-bearing mouse model, a suspension containing 1 × 10^6^ of 4T1 cells in PBS buffer was subcutaneously injected into the right flank of each female BALB/c mouse aged 6–8 weeks. To validate the real-time activation of ratiometric fluorescence changes of BC@Z-M in hypoxic tumors, BC@Z-M (50 μL, 1 mg mL^‒1^ based on BN-O) was first administered to tumor-bearing mice via intratumoral injection. For control, an equivalent dose of BC@Z-M was also injected subcutaneously into the left flanks of BALB/c mice without tumor inoculation. The alterations in NIR-I and NIR-II fluorescence at both the tumor site and the contralateral subcutaneous site were monitored at various time points post-injection using IVIS (NightOWL II LB983) and SWIR imaging system (Wuhan Grand-imaging Technology Co., LTD), respectively. The SWIR imaging system was equipped with an infrared InGaAs scientific-grade camera with a long-pass (LP) filter of 1000 nm. Image J software 1.53a was employed for data analysis.

To assess the hypoxic tumor imaging potential of NPs following intravenous administration, the mice were injected intravenously with BC@Z or BC@Z-M (200 μL, 1 mg mL^‒1^ based on BN-O). NIR-II fluorescence imaging was conducted using the SWIR imaging system at various time points following NP injection. Additionally, at 24 h post-administration, tumors and major organs (heart, liver, spleen, lung, and kidneys) were harvested for ex vivo NIR-II fluorescence. Photoacoustic imaging in living mice was performed using a VisualSonics Vevo LAZR small animal photoacoustic multimodal imaging system (Canada). The parameters for PA imaging system were set as follows: frequency = 30 MHz; wavelength range = 680–970 nm; PA gain = 33 dB; gain = 13 dB; depth/width = 11.00/15.36 mm. The ultrasound images and photoacoustic images were simultaneously acquired, and the merged PA/US images were generated by overlaying each photoacoustic image in pseudo-color onto the gray-scale ultrasound image.

### In vivo prophylactic vaccination experiments

4T1 cells were incubated with different formulations (PBS, BC@Z, or BC@Z-M; 20 μg mL^‒1^ based on BN-O) at 37 °C for 24 h. Then, cells in the light irradiation groups were irradiated with NIR light (1.0 W cm^‒2^) for 5 min. The mice were randomly divided into five groups (*n* = 5 mice per group) and immunized on day 0 by subcutaneous injection of 2 × 10^6^ various pretreated cells (in 100 µL sterile PBS) into the left flank of mice. The control group received an injection of PBS (no vaccine). Seven days later, the mice were challenged with live 4T1 cancer cells via subcutaneous injection of 1 × 10^6^ cells into the right flank. The tumor growth and body weights of the mice were monitored every two days.

### In vivo anticancer study

To establish the 4T1 tumor model, 100 μL of cold PBS containing 1 × 10^6^ of 4T1 murine breast cancer cells was injected into the right flank of female BALB/c mice (aged 7 weeks) on day −7. By day 0, the tumor volume reached approximately 100 mm^3^, at which point the tumor-bearing mice were randomly allocated into six groups: PBS, PBS + L, C@Z, BC@Z, BC@Z + L, and BC@Z-M + L. On day 0, the mice in each group were intravenously administered with PBS, PBS, C@Z, BC@Z, BC@Z, and BC@Z-M, respectively. For the groups designated for light irradiation, the tumor areas were irradiated at 24 h post-injection using a 730 nm laser (1.0 W cm^−2^) for 6 min. To monitor the real-time temperature increase induced by PTT effect at the tumor site, in vivo photothermal imaging was conducted using a thermal infrared camera (FLK-Ti200) during 730 nm laser irradiation. The intravenous administration of different formulations and phototherapy at the tumor site was repeated every 3 days for a total of 3 times. During the treatment process, an additional 5 × 10^5^ of 4T1 cells were intravenously injected into mice on day 6 to simulate malignant tumor invasion. The method of additional intravenous injection of tumor cells into tumor-bearing mice is commonly employed to replicate hematogenous metastasis. The body weights and tumor volumes of mice were meticulously recorded every two days, while the survival of mice was monitored daily to obtain survival rate curves. Tumor volume was calculated using the formula: Volume = (Length × Width × Width)/2.

On day 16, the mice were euthanized, and their major organs (heart, liver, spleen, lung, and kidneys) as well as tumors were harvested for further analysis. The tumor tissues were sectioned and subjected to hematoxylin and eosin (H&E) staining and terminal deoxynucleotidyl transferase dUTP nick end labeling (TUNEL) immunofluorescence staining. Additionally, tumor sections were stained with anti-CD31 antibody (Abcam, ab222783, 1:200) to assess the vascular shutdown induced by vascular disrupting agent-contained nanoformulations. For the evaluation of lung metastasis, the lungs were stained with India ink to directly observe the lung metastasis nodes. Furthermore, the lung and liver were sectioned and subjected to H&E staining to evaluate tumor metastasis in these tissues.

In the tumor rechallenge experiment, 2 × 10^5^ of 4T1 cells were injected into the left flank of mice that had survived in the BC@Z-M + L group on day 40. Simultaneously, an equivalent number of 4T1 cells were injected into the untreated naive mice as a control. Tumor volume and survival of the re-challenged mice were recorded every other day. A bilateral tumor model was further established to assess the abscopal effect. To this end, 10^6^ of 4T1 cells were injected into the right flank of BALB/c mice to form the primary tumor. Six days later, 5 × 10^5^ of 4T1 cells were inoculated into the left flank of the same mice to build a distant tumor, mimicking cancer metastasis. When the primary tumor reached ~100 mm^3^, the mice were randomly divided into two groups (*n* = 5 mice per group): PBS + L and BC@Z-M + L. On days 0, 3, and 6, the mice were intravenously injected with either PBS or BC@Z-M (200 μL, 1 mg mL^−1^ based on BN-O). For phototherapy, only the primary tumor was exposed to 730 nm laser irradiation (1.0 W cm^−2^, 6 min) at 24 h post-injection of the different formulations, while the distant left tumor remained untreated. The sizes of both bilateral tumors and the body weights of the mice were monitored every other day. On day 22, the mice were euthanized, and their tumor tissues were harvested for further tumor inhibition and immune analysis. For CD4^+^/CD8^+^ T-cell depletion, the mice were intraperitoneally injected with 100 μg of anti-CD4 antibody (BioXCell, clone GK1.5) or 100 μg of anti-CD8α antibody (BioXCell, clone 2.43) every 4 days starting from day 0.

### Antitumor immunity analysis

Initially, an equal amount of tumor tissue collected from mice in each group was homogenized, and the cytokines, including TNF-α, IL-6, and IFN-β, in the tumor tissue were analyzed using ELISA, following the manufacturer’s instructions. Additionally, on day 16, tumor-draining lymph nodes (TDLNs), spleens, and tumor tissues from each group of mice were harvested for flow cytometry analysis of immune cell populations. These tissues were homogenized to produce a single-cell suspension using a homogenizer. The cell suspensions were then collected by centrifugation and treated with a modified red blood cell (RBC) lysis buffer to remove red blood cells. Following wash with 1× PBS, the cell suspensions were passed through a 70 μm filter and transferred to flow tubes for staining. DC maturation in TDLNs was detected using anti-CD11c-FITC (Biolegend, cat. no. 117306, Clone: N418, 1:200 dilution), anti-CD80-PE (Biolegend, cat. no. 104707, Clone: 16-10A1, 1:200 dilution), and anti-CD86-APC (Biolegend, cat. no. 105011, Clone: GL-1, 1:200 dilution), following instructions provided by the manufacturer. The CD4^+^ T cells and CD8^+^ T cells infiltration in the tumor were detected using anti-CD3-FITC (Biolegend, cat. no. 100203, Clone: 17A2, 1:200 dilution), anti-CD4-BV421 (Biolegend, cat. no. 100543, Clone: RM4-5, 1:200 dilution), and anti-CD8-APC (Biolegend, cat. no. 100711, Clone: 53-6.7, 1:200 dilution) according to the standard protocols. Tregs in the tumor were detected using anti-CD3-FITC (Biolegend, cat. no. 100203, Clone: 17A2, 1:200 dilution), anti-CD4-BV421 (Biolegend, cat. no. 100543, Clone: RM4-5, 1:200 dilution), and anti-Foxp3-PE (Biolegend, cat. no. 320007, Clone: 150D, 1:200 dilution) referring to manufacturer’s protocol. TAM cells in the tumor were detected using anti-F4/80-PE (Biolegend, cat. no. 123109, Clone: BM8, 1:200 dilution), anti-CD11b-FITC (Biolegend, cat. no. 101205, Clone: M1/70, 1:200 dilution), anti-CD86-APC (Biolegend, cat. no. 105011, Clone: GL-1, 1:200 dilution), and anti-CD206-BV421 (Biolegend, cat. no. 141717, Clone: C068C2, 1:200 dilution) according to the standard protocols. T_EM_ cells in the spleen were detected using anti-CD3-FITC (Biolegend, cat. no. 100203, Clone: 17A2, 1:200 dilution), anti-CD8-APC (Biolegend, cat. no. 100711, Clone: 53-6.7, 1:200 dilution), anti-CD44-PE (Biolegend, cat. no. 103007, Clone: IM7, 1:200 dilution), and anti-CD62L-BV605 (Biolegend, cat. no. 104437, Clone: MEL-14, 1:200 dilution) referring to manufacturer’s protocol. For tertiary lymphoid structure staining, tumor slides were fixed in neutral buffered formalin and embedded in paraffin. Non-specific binding was blocked using a 5% bovine serum albumin blocking buffer, followed by incubation overnight at 4 °C with primary antibodies (anti-CD4, anti-CD8, and anti-CD20; 1:200 dilution). After three washes with PBS, the slides were incubated for 1 h in the dark with appropriate fluorescent secondary antibodies (1:400 dilution). Following additional washes with PBS, cell nuclei were stained with DAPI. The multiplexed stained slides were imaged using a Zeiss microscope.

### Transcriptomic analysis

Tumor tissues from the mice treated with or without BC@Z-M + L were collected, and the total RNA was extracted using TRIzol (Invitrogen) following the manufacturer’s protocol. RNA sequencing libraries were prepared using the TruSeq RNA LT Sample Prep Kit v2 (Illumina). The prepared libraries were then sequenced on an Illumina NovaSeq™ 6000 sequencer. Quality control of the RNA data was conducted using FastQC. Clean reads were aligned to the mouse reference genome (Ensembl v107) using HISAT2. The aligned reads for each sample were then assembled into transcripts using StringTie with default parameters. All individual transcriptomes were merged to create a comprehensive transcriptome using gffcompare (https://github.com/gpertea/gffcompare/). Following the generation of the final transcriptome, StringTie was used to estimate the expression levels of all transcripts.

### In vivo biocompatibility evaluation

The in vivo biosafety profiles of BC@Z-M were evaluated in healthy BALB/c mice. Briefly, BC@Z-M (200 μL, 1 mg mL^‒1^ based on BN-O) were intravenously injected into healthy mice (*n* = 3 mice) on days 0, 3, and 6, respectively. The PBS-treated healthy mice served as controls (*n* = 3 mice). The body weights of mice were monitored every other day. On day 10, all mice were sacrificed, and blood samples were collected for biochemical analyses and assessment of indicators related to liver and kidney functions. Additionally, the major organ tissues (heart, liver, spleen, lung, and kidneys) from both PBS-treated and BC@Z-M-injected mice were subjected to H&E staining after paraffin sectioning to observe morphological and pathological changes.

### Statistical analysis

All statistical analyses were conducted using GraphPad Prism 8.0.2. Flow cytometry data were analyzed using FlowJo v10.8.1 software. Statistical comparisons were performed using one-way ANOVA for multiple comparisons or two-tailed Student’s *t*-test for comparisons between two groups. Data are presented as mean ± standard deviation (SD). The *p-*value of less than 0.05 was considered statistically significant.

### Reporting summary

Further information on research design is available in the Nature Portfolio Reporting Summary linked to this article.

## Supplementary information

Supplementary Information

Description of Additional Supplementary Files

Supplementary Data 1

Reporting Summary

## Source data

Source Data

Transparent Peer Review file

## Data Availability

The RNAseq data used in this study are available in the Gene Expression Omnibus (GEO) database with accession number GSE278201. The remaining data that support the findings of this study are available within the Article, Supplementary information, or Source Data file. Source data are provided with this paper.
