# Supplementary material for: A hypoxia-activated and microenvironment-remodeling nanoplatform for multifunctional imaging and potentiated immunotherapy of cancer
Source: Nat Commun. 2024 Nov 29;15:10395. doi: 10.1038/s41467-024-53906-x (PMC11607447; doi:10.1038/s41467-024-53906-x)
Supplement: Supplementary file 1 — Supplementary Information [file 41467_2024_53906_MOESM1_ESM.pdf]

Supplementary Information for

A hypoxia-activated and microenvironment-remodeling nanoplatform for multifunctional imaging and potentiated immunotherapy of cancer

Jianwen Song,[1] He Wang,[2] Xue Meng,[1] Wen Li[3] & Ji Qi[1]*

[1]State Key Laboratory of Medicinal Chemical Biology, Key Laboratory of Bioactive Materials, Ministry of Education, and College of Life Sciences, Nankai University, Tianjin, China.

[2]Department of Urology, The First Affiliated Hospital of Soochow University, Suzhou, China.

[3]Tianjin Key Laboratory of Biomedical Materials and Key Laboratory of Biomaterials and Nanotechnology for Cancer Immunotherapy, Institute of Biomedical Engineering, Chinese Academy of Medical Sciences and Peking Union Medical College, Tianjin, China.

*Corresponding author. E-mail: qiji@nankai.edu.cn

Supplementary Methods:

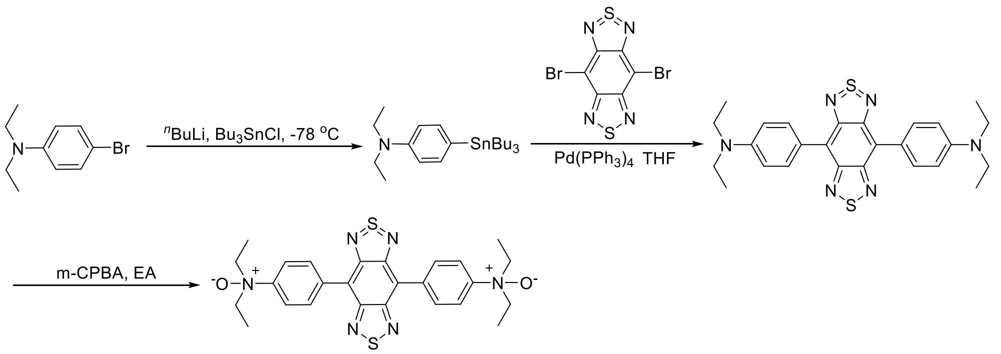

Supplementary Fig. 1 Synthetic route to BN-O.

Syntheses processes and characterizations

Synthesis of *N*,*N*-diethyl-4-(tributylstannyl)aniline

4-Bromo-*N*,*N*-diethylaniline (1.37 g, 6 mmol) was dissolved in anhydrous THF (40 mL) in argon atmosphere. The reaction was cooled to −78 °C using the mixture of dry ice-acetone, and maintained for 30 min, after which *n*-butyllithium (^{n}BuLi) (2.5 M hexane solution, 2.4 mL, 6 mmol) was added. After stirring at this temperature for 2 h, tri-butyltin chloride (1.63 mL, 6 mmol) was introduced, and the mixture was allowed to slowly warm to room temperature, then stirred overnight. Subsequently, water was added to quench the reaction, and the resulting mixture was extracted with CH_2Cl_2 three times. The collected organic phase was combined, dried using $MgSO_4$. The solvent was evaporated, and the obtained crude product was used directly.

Synthesis of 4,4'-(benzo[1,2-*c*:4,5-*c*']bis[1,2,5]thiadiazole-4,7-diyl)bis(*N*,*N*-diethylaniline) (BN)

N,*N*-diethyl-4-(tributylstannyl)aniline (1.31 g, 3 mmol), 4,7-dibromobenzo[1,2-*c*:4,5-*c*']bis([1,2,5]thiadiazole) (0.35 g, 1 mmol), and $Pd(PPh_3)_4$ (0.35 g, 0.3 mmol) were combined in argon atmosphere, following which anhydrous THF (50 mL) was introduced. The mixture was heated to reflux, stirring continuously for 24 h. Upon cooling to room temperature, water was added, and the resulting mixture was extracted with CH_2Cl_2 three times. The collected organic phase was consolidated, dried using $MgSO_4$, and concentrated. The crude product was purified by column chromatography on silica gel

using CH_2Cl_2/hexane (v/v 1:2) as the eluting solvent, resulting in 4,4'-(benzo[1,2-*c*:4,5-*c*']bis[1,2,5]thiadiazole-4,7-diyl)bis(*N*,*N*-diethylaniline) as a green solid (73% yield). ^{1}H NMR (400 MHz, $CDCl_3$) δ 8.22 (d, J = 9.0 Hz, 4H), 6.92 (d, J = 9.0 Hz, 4H), 3.49 (m, J = 7.0 Hz, 8H), 1.26 (t, J = 7.1 Hz, 12H). ^{13}C NMR (100 MHz, $CDCl_3$) δ 152.76, 147.96, 133.09, 122.62, 119.70, 111.23, 44.47, 12.87. HRMS (MALDI-TOF, *m*/*z*): $[M + H]^+$ calcd. for $C_{26}H_{28}N_6S_2$, 489.1897; found, 489.1890.

Synthesis of 4,4'-(benzo[1,2-*c*:4,5-*c*']bis[1,2,5]thiadiazole-4,7-diyl)bis(*N*,*N*-diethylaniline oxide) (BN-O)

The synthesis of BN-O was carried out under normoxic condition. 200 mg of BN was dissolved in 10 mL of ethyl acetate and then the reaction mixture was placed in an ice bath. After a slow dropwise addition of 210 mg of ethyl acetate solution of meta-chloroperoxybenzoic acid (*m*-CPBA), the resulting mixture was stirred for 4 hours. It was concentrated and purified on a silica gel column using methanol as eluent. The mixed eluent was evaporated and the product was evaporated and redissolved in dichloromethane. The mixture was filtered to remove the silica gel. The dichloromethane solution was condensed to give a purplish red powder in 86% yield. ^{1}H NMR (400 MHz, $CDCl_3$) δ 8.30 (d, J = 8.5 Hz, 4H), 8.02 (d, J = 8.5 Hz, 4H), 3.80–3.62 (m, 8H), 1.20 (t, J = 6.9 Hz, 12H). ^{13}C NMR (100 MHz, $CDCl_3$) δ 152.65, 149.77, 135.00, 132.44, 121.94, 67.02, 49.96, 29.65, 8.59. HRMS (MALDI-TOF, *m*/*z*): $[M + H]^+$ calcd. for $C_{26}H_{28}N_6O_2S_2$, 521.1795; found, 521.1792.

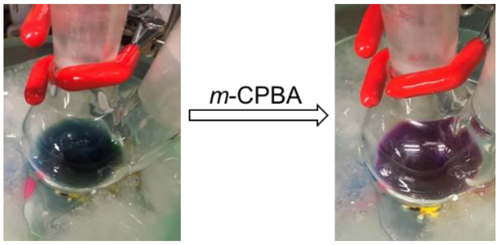

Supplementary Fig. 2 Photographs showing the color change from BN to BN-O throughout the oxidation reaction.

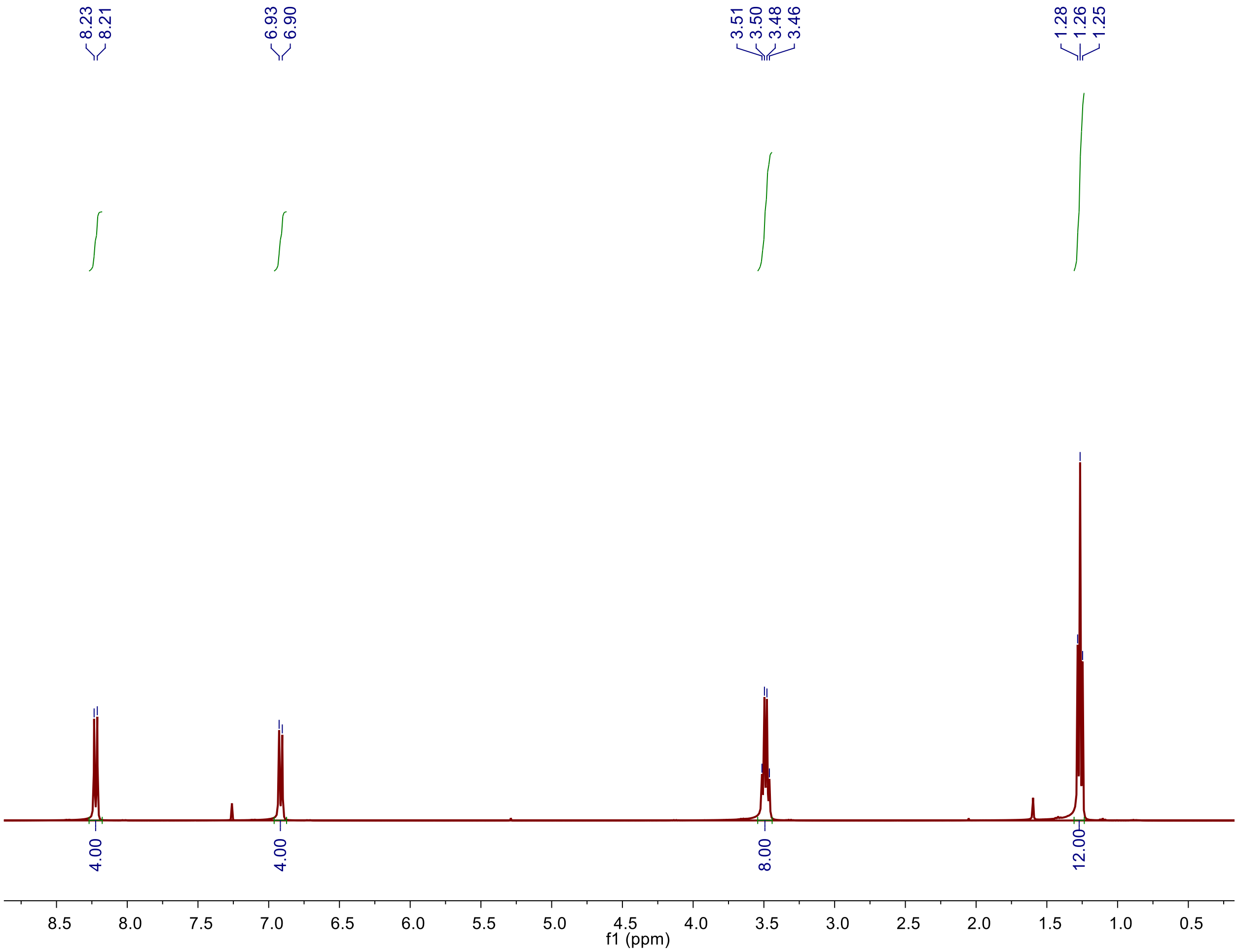

Supplementary Fig. 3 ^1H NMR spectrum of BN in $CDCl_3$ at 298 K.

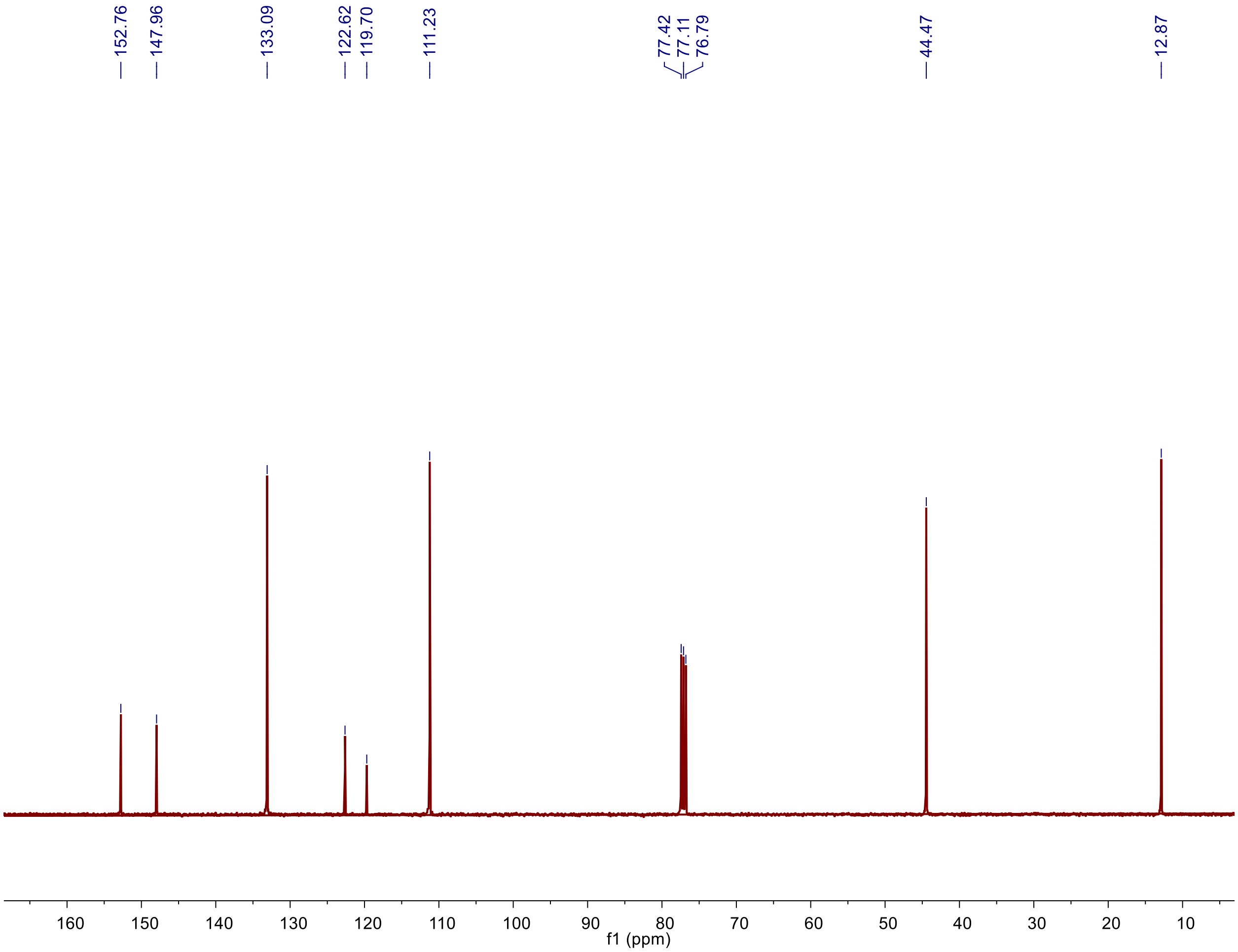

Supplementary Fig. 4 ^{13}C NMR spectrum of BN in $CDCl_3$ at 298 K.

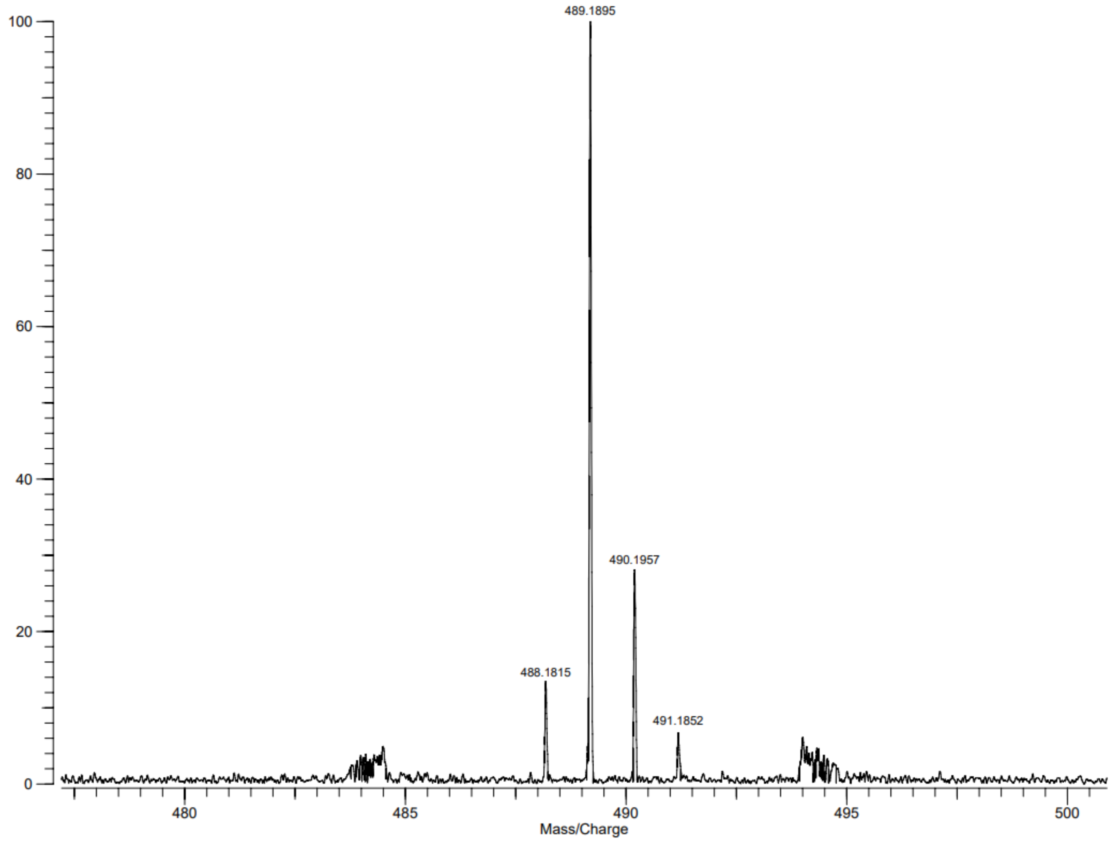

Supplementary Fig. 5 HRMS of BN.

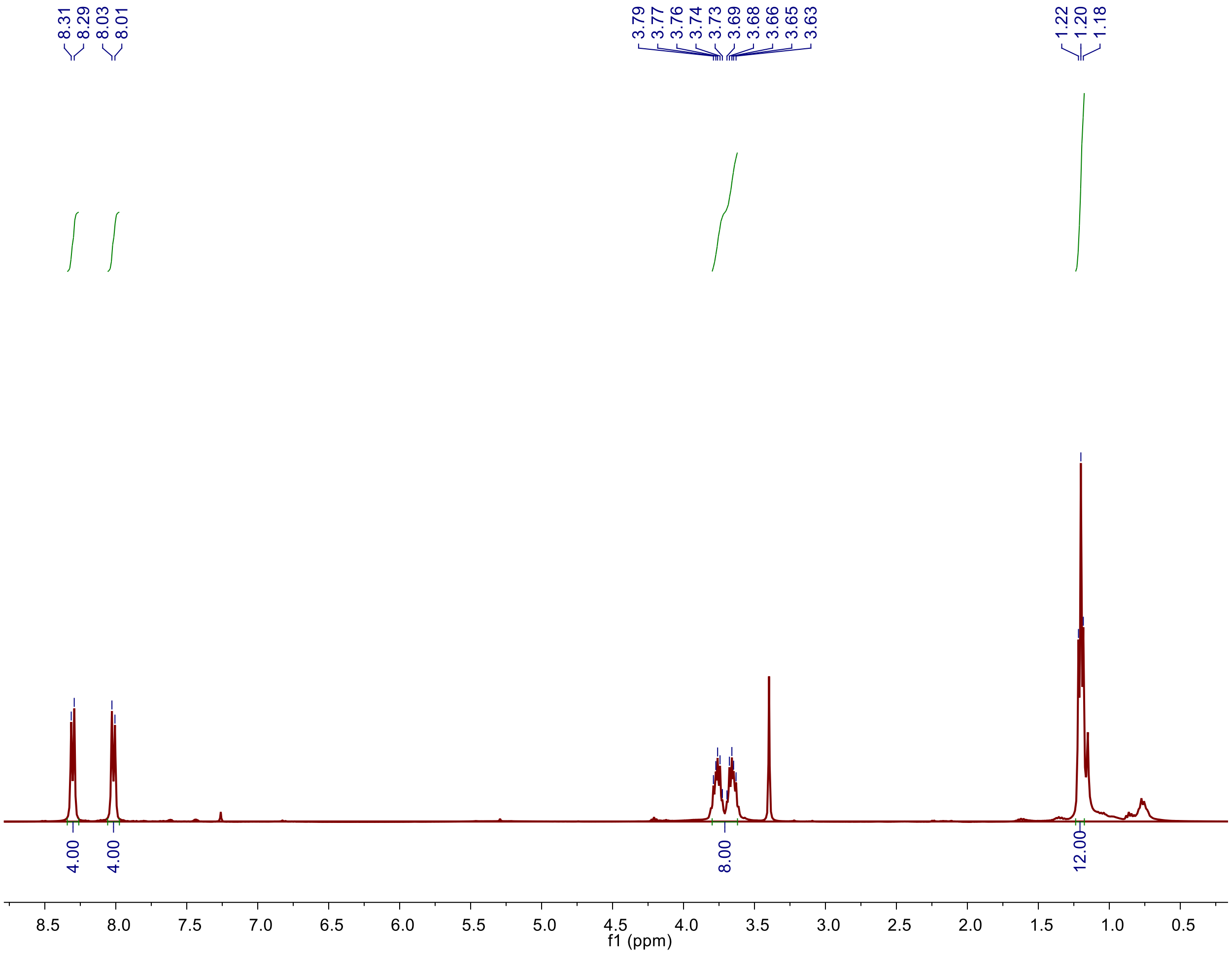

Supplementary Fig. 6 ^{1}H NMR spectrum of BN-O in $CDCl_3$ at 298 K.

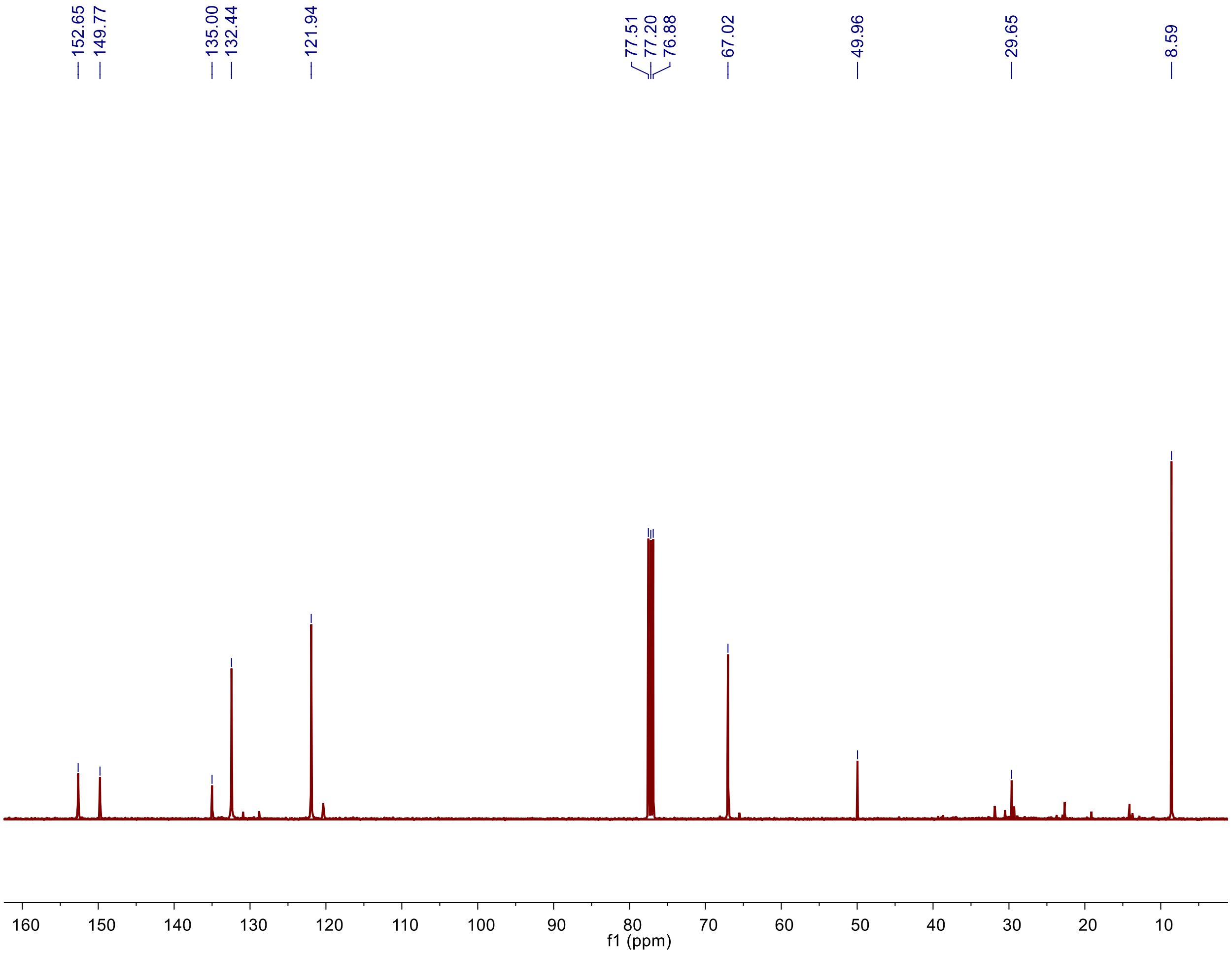

Supplementary Fig. 7 ^{13}C NMR spectrum of BN-O in $CDCl_3$ at 298 K.

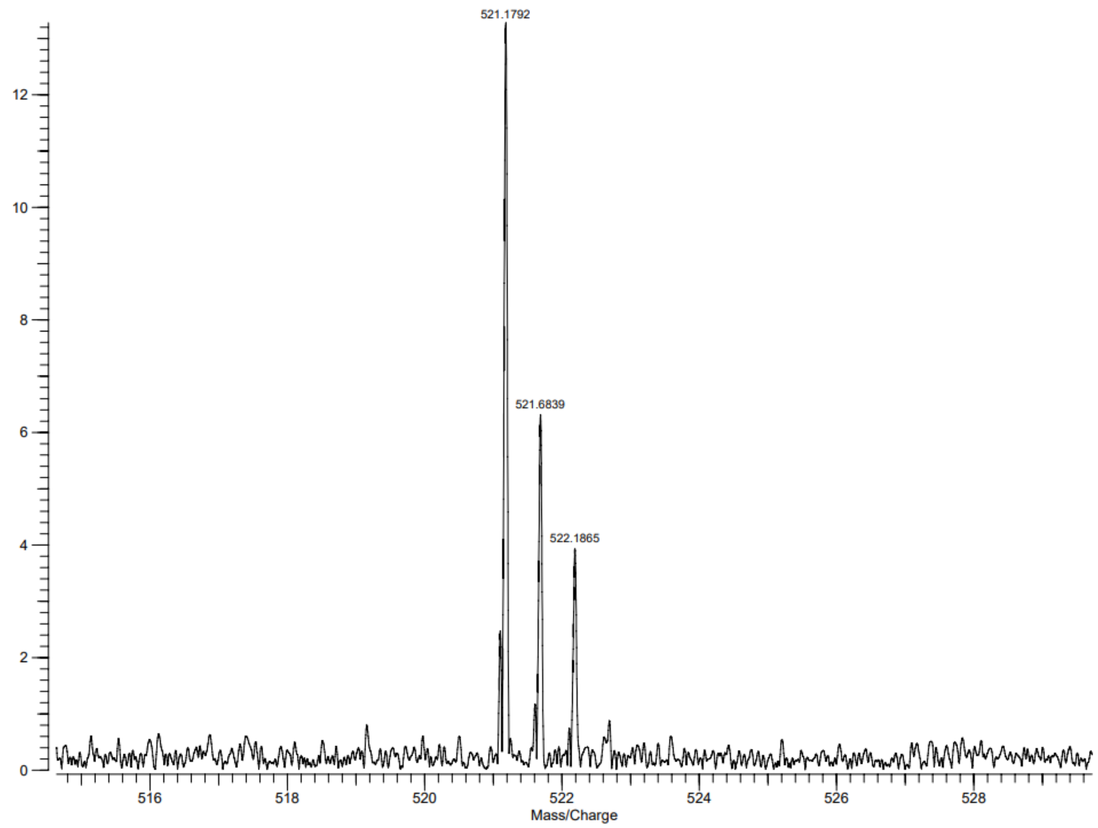

Supplementary Fig. 8 HRMS of BN-O.

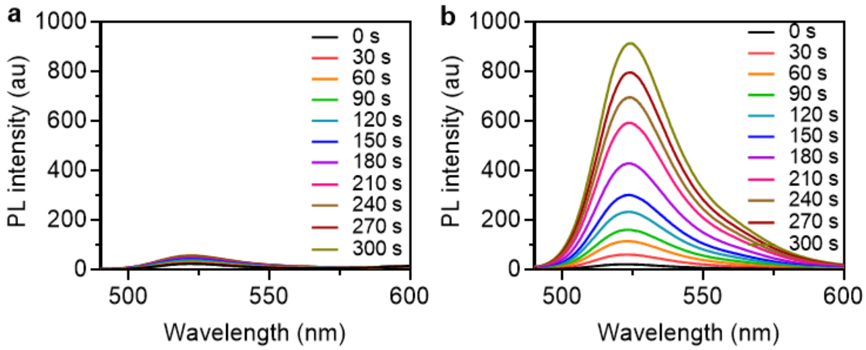

Supplementary Fig. 9 PL spectra of DCFH-DA (10 μM) in the presence of (a) BN-O and (b) BN under 730 NIR light (1.0 W cm^{-2}) irradiation for different time. Experiment was repeated three times independently with similar results. Source data are provided as a Source Data file.

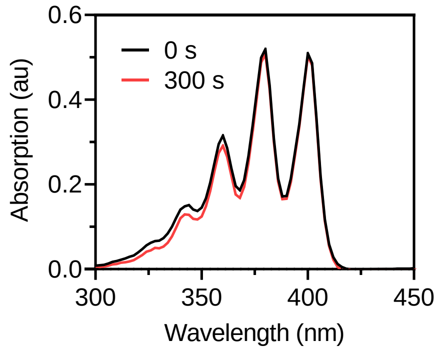

Supplementary Fig. 10 Absorption spectra of ABDA (10 μM) in the presence of BN before and after 730 NIR light (1.0 W cm^{-2}) irradiation. Experiment was repeated three times independently with similar results. Source data are provided as a Source Data file.

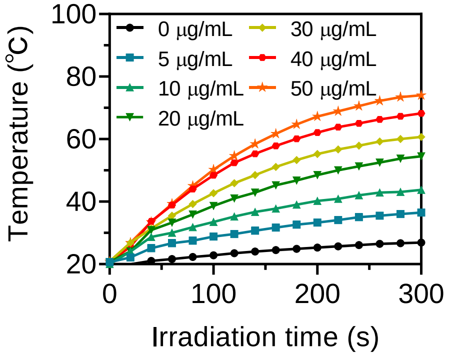

Supplementary Fig. 11 Temperature changes of different concentrations of BN under 730 nm NIR light (1.0 W cm^{-2}) irradiation. Experiment was repeated three times independently with similar results. Source data are provided as a Source Data file.

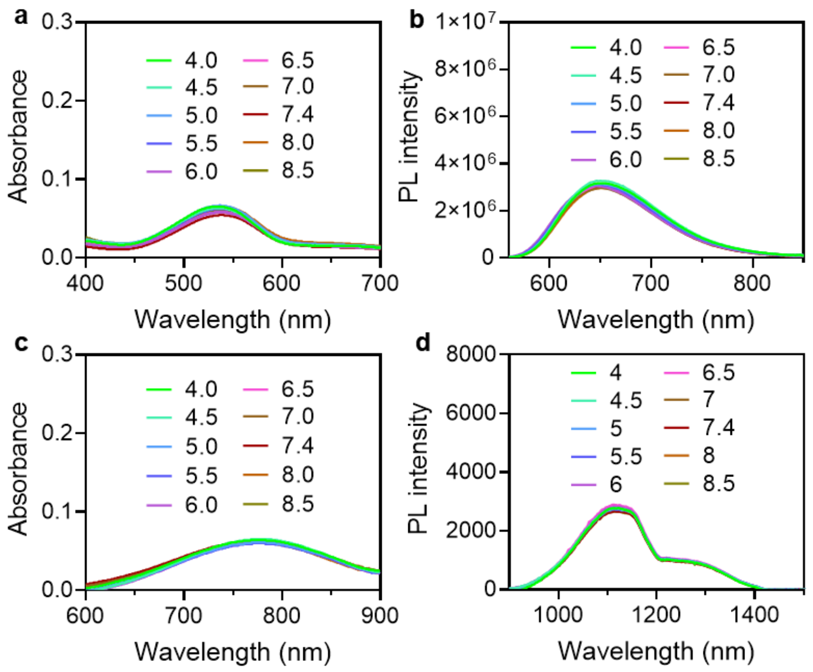

Supplementary Fig. 12 Absorption and PL spectra of (a,b) BN-O and (c,d) BN in various pH conditions. Experiment was repeated three times independently with similar results. Source data are provided as a Source Data file.

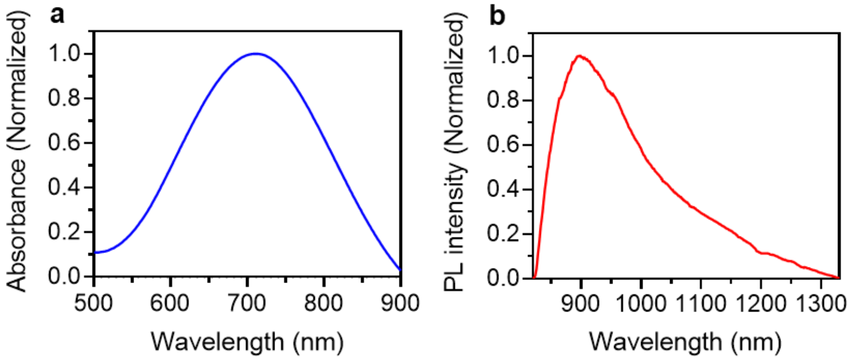

Supplementary Fig. 13 (a) Absorption and (b) PL spectra of BN. Experiment was repeated three times independently with similar results. Source data are provided as a Source Data file.

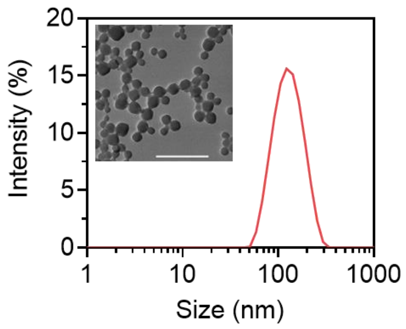

Supplementary Fig. 14 Representative DLS result and TEM image of BC@Z. Scale bar: 500 nm. Experiment was repeated three times independently with similar results. Source data are provided as a Source Data file.

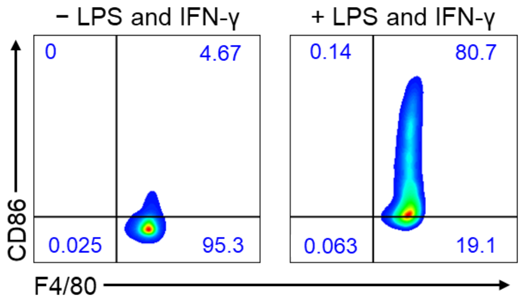

Supplementary Fig. 15 Flow cytometry plots of M1 phenotype (F4/80^{+} CD86^{+}) in RAW264.7 cells before and after treating with LPS and IFN-γ.

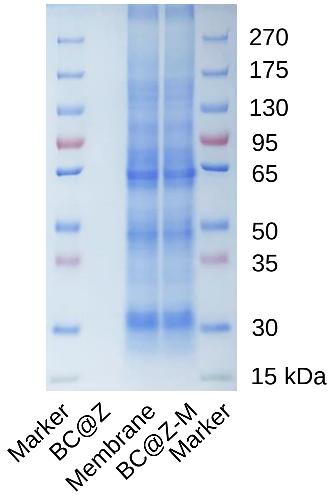

Supplementary Fig. 16 SDS-PAGE showing the protein profiles of BC@Z, M1-like macrophage membrane, and BC@Z-M.

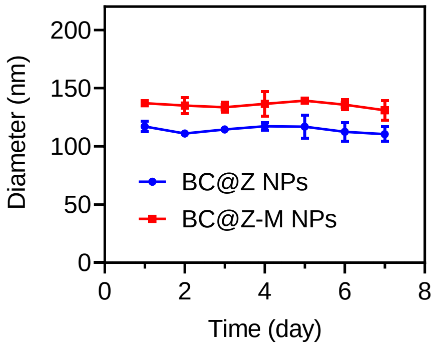

Supplementary Fig. 17 The hydrodynamic diameter of BC@Z-M after storage in PBS for seven days monitored using DLS. Data are presented as mean ± SD (n = 3 independent samples). Source data are provided as a Source Data file.

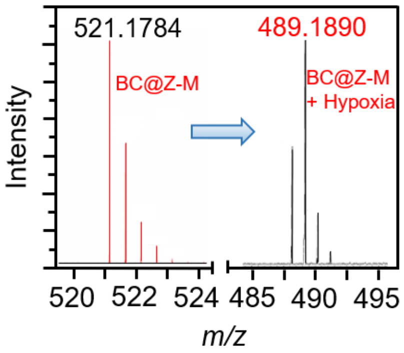

Supplementary Fig. 18 HRMS analysis showing the conversion of BN-O in BC@Z-M to BN upon hypoxia treatment. Experiment was repeated three times independently with similar results.

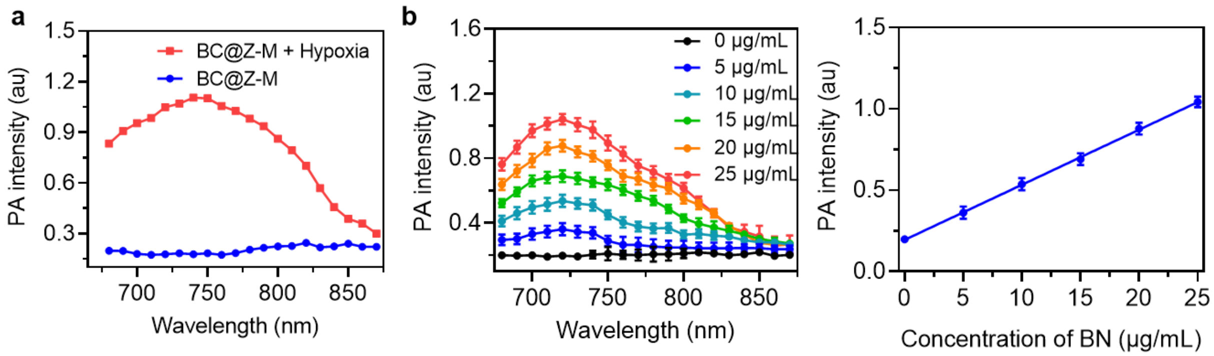

Supplementary Fig. 19 (**a**) PA intensity of BC@Z-M before and after hypoxia treatment at different wavelengths. (**b**) PA profiles and corresponding PA intensity of various concentrations of BC@Z-M after hypoxia activation. Data are presented as mean ± SD (*n* = 3 independent samples). Source data are provided as a Source Data file.

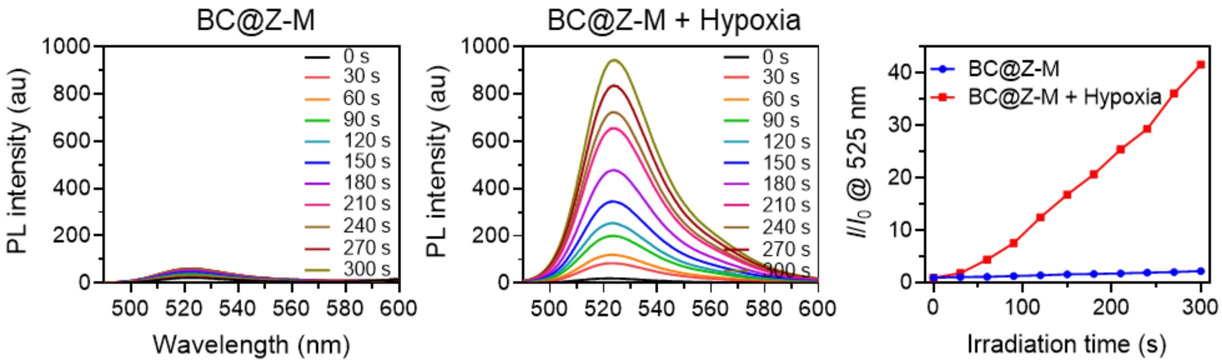

Supplementary Fig. 20 PL intensity of DCFH-DA (10 μM) at 525 nm in the presence of BC@Z-M before and after hypoxia treatment under the irradiation of 730 nm NIR light (1.0 W cm^{-2}) for various durations. I_0 and I denote the PL intensity of DCFH-DA at 525 nm before and after 730 nm NIR light irradiation, respectively. Experiment was repeated three times independently with similar results. Source data are provided as a Source Data file.

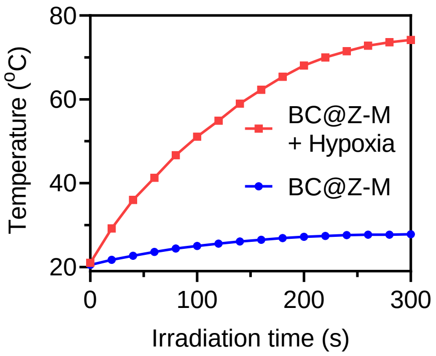

Supplementary Fig. 21 Temperature changes of BC@Z-M before and after hypoxia treatment under 730 nm NIR light (1.0 W cm^{-2}) irradiation for different time. Experiment was repeated three times independently with similar results. Source data are provided as a Source Data file.

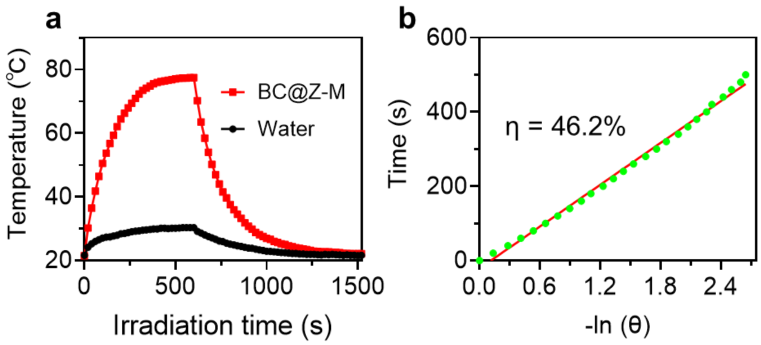

Supplementary Fig. 22 (**a**) The photothermal behavior of water and BC@Z-M under hypoxia conditions. The samples were irradiated with 730 nm laser for 10 min, then the laser was removed, and the samples were naturally cooling down. (**b**) Plots of irradiation time versus -ln(θ) for BC@Z-M. Experiment was repeated three times independently with similar results. Source data are provided as a Source Data file.

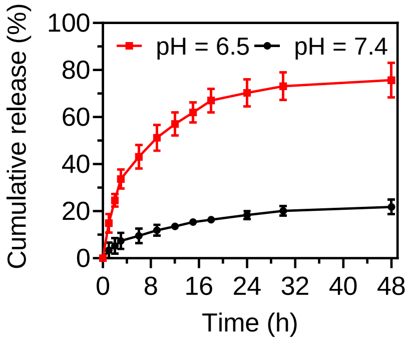

Supplementary Fig. 23 The release profiles of BN-O from BC@Z-M at different pH conditions. Data are presented as mean ± SD (*n* = 3 independent samples). Source data are provided as a Source Data file.

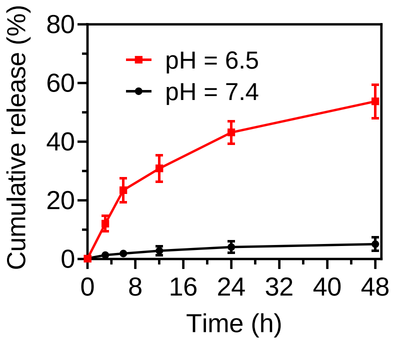

Supplementary Fig. 24 The release profiles of Zn^{2+} from BC@Z-M at different pH conditions. Data are presented as mean ± SD (*n* = 3 independent samples). Source data are provided as a Source Data file.

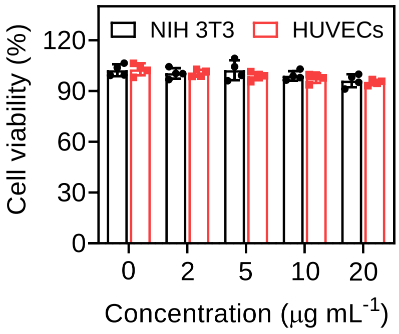

Supplementary Fig. 25 The viabilities of NIH 3T3 and HUVEC cells assessed using MTT assay following treatment with varying concentrations of BC@Z-M. Data are presented as mean ± SD (n = 4 independent samples). Source data are provided as a Source Data file.

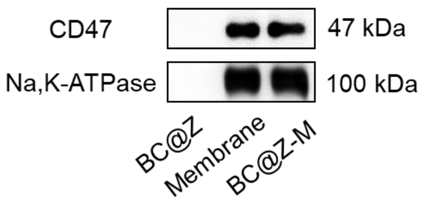

Supplementary Fig. 26 Western blot analysis of the expression of CD47 on BC@Z, M1-like macrophage membrane, and BC@Z-M. Experiment was repeated three times independently with similar results.

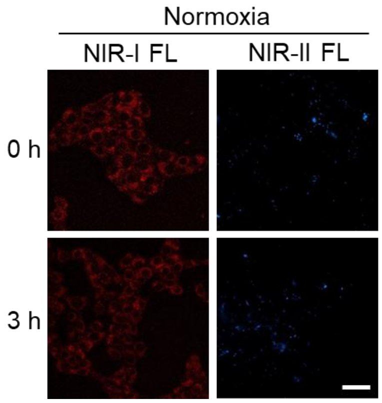

Supplementary Fig. 27 NIR-I and NIR-II microscopic fluorescence images of 4T1 cells after incubation with BC@Z-M under normoxic conditions for 0 or 3 h. Scale bar: 100 μm. Experiment was repeated three times independently with similar results.

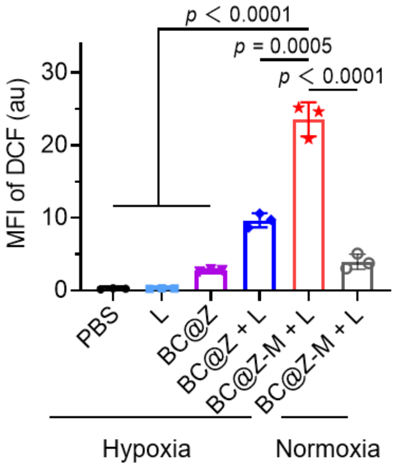

Supplementary Fig. 28 Mean fluorescence intensity (MFI) of DCF in Fig. 4h showing the ROS generation in 4T1 cells after different treatments. Data are presented as mean ± SD (*n* = 3 independent samples). Statistical significance was determined using one-way ANOVA. Source data are provided as a Source Data file.

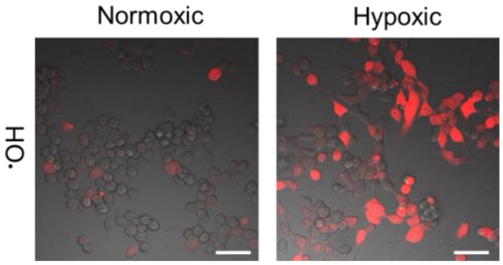

Supplementary Fig. 29 Representative CLSM images showing the type-I ROS generation in 4T1 cancer cells following "BC@Z-M + L" treatment under normoxic or hypoxic condition. Scale bars: 50 µm. HPF (50 µM), a specific probe for hydroxyl radicals (•OH), was used to detect the intracellular type-I ROS. Experiment was repeated three times independently with similar results.

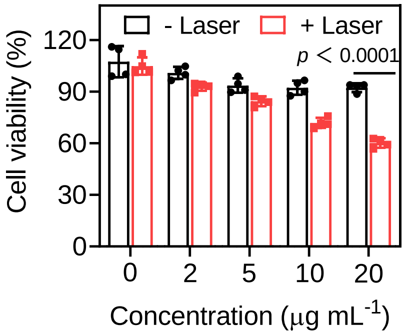

Supplementary Fig. 30 Viability of 4T1 tumor cells in normoxia condition with the treatment of different concentrations of BC@Z-M without or with 730 nm light (1.0 W cm^{-2}) irradiation. Data are presented as mean ± SD (n = 4 independent samples). Statistical significance was determined using two-tailed Student's t-test. Source data are provided as a Source Data file.

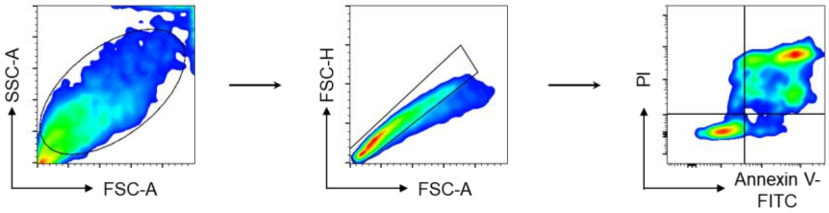

Supplementary Fig. 31 Gating strategy for the flow cytometry analysis of the proportions of Annexin V-FITC and PI co-stained 4T1 cells in Supplementary Fig. 32.

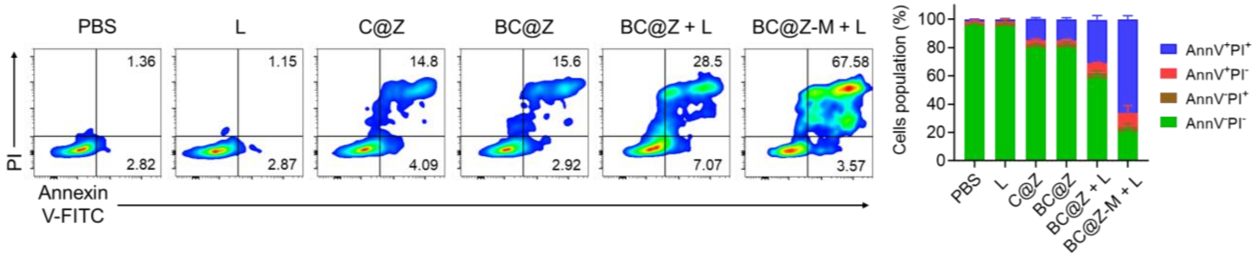

Supplementary Fig. 32 Representative flow cytometry plots and quantitative analyses of the populations of annexin-V and PI co-stained 4T1 cells following various treatments. Data are presented as mean ± SD (n = 3 independent samples). Source data are provided as a Source Data file.

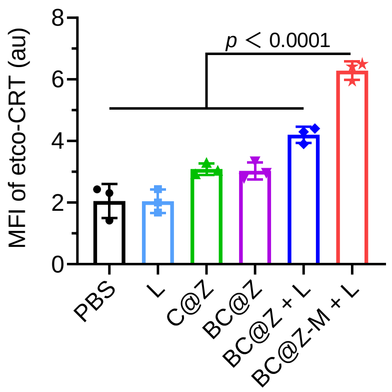

Supplementary Fig. 33 MFI of etco-CRT on 4T1 tumor cells according to Fig. 5a. Data are presented as mean ± SD (n = 3 independent samples). Statistical significance was determined using one-way ANOVA. Source data are provided as a Source Data file.

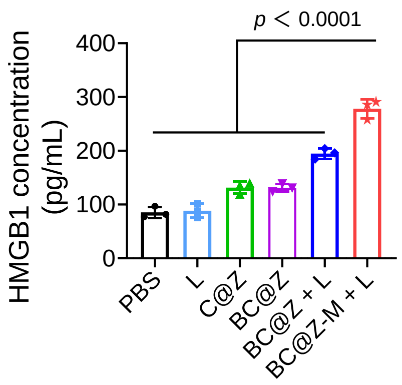

Supplementary Fig. 34 Analysis of HMGB1 concentrations in the supernatant of 4T1 tumor cells after different treatments by ELISA assay. Data are presented as mean ± SD (n = 3 independent samples). Statistical significance was determined using one-way ANOVA. Source data are provided as a Source Data file.

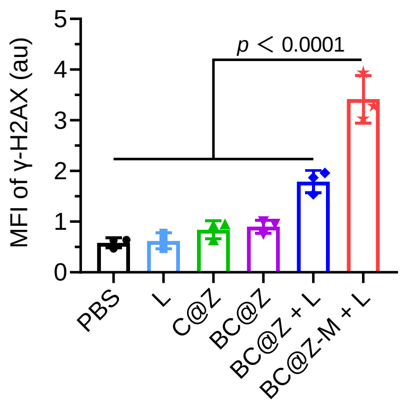

Supplementary Fig. 35 MFI of γ-H2AX in 4T1 tumor cells in Fig. 5f. Data are presented as mean ± SD (n = 3 independent samples). Statistical significance was determined using one-way ANOVA. Source data are provided as a Source Data file.

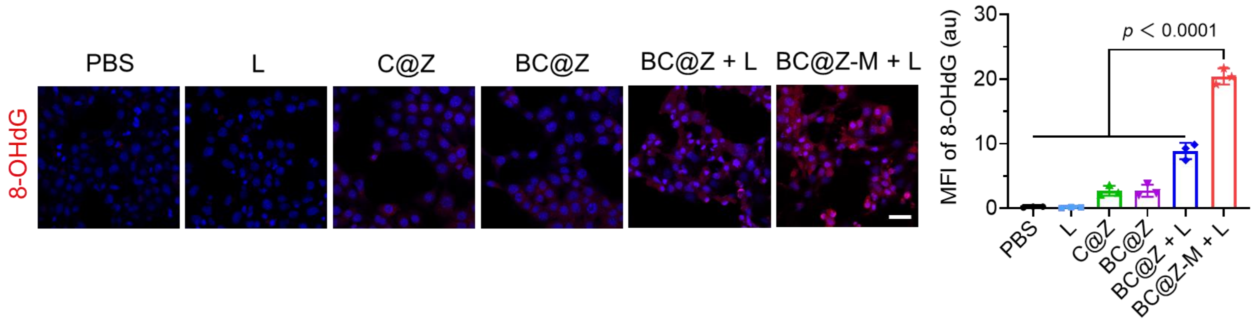

Supplementary Fig. 36 4T1 tumor cells were treated with the indicated formulations, followed by light irradiation in selected groups. After 24 h, the cells were stained with anti-8-OHdG antibody to label oxidized DNA, and imaged using confocal microscopy. Scale bars, 50 μm. The corresponding quantitative analysis of the 8OHdG fluorescence intensity was conducted. Data are presented as mean ± SD (n = 3 independent samples). Statistical significance was determined using one-way ANOVA. Source data are provided as a Source Data file.

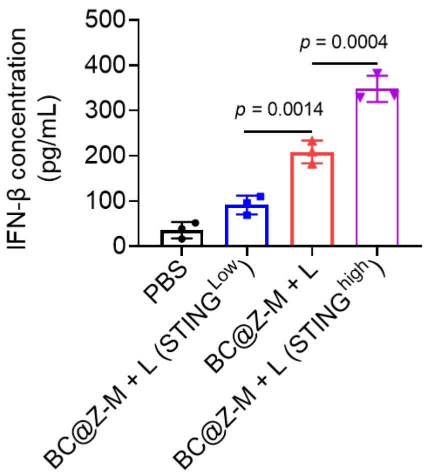

Supplementary Fig. 37 4T1 tumor cells with normal, knockdown, or overexpressed STING expression were treated with BC@Z-M + L, and IFN-β expression levels in each group were then measured using ELISA kit. Data are presented as mean ± SD (n = 3 independent samples). Statistical significance was determined using one-way ANOVA. Source data are provided as a Source Data file.

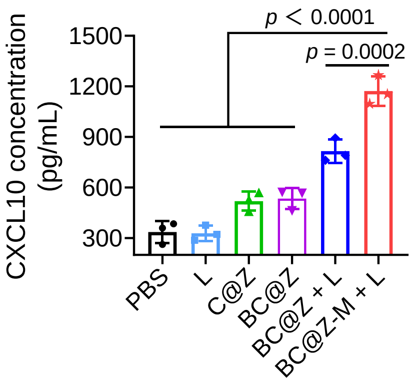

Supplementary Fig. 38 The expression levels of CXCL10 from 4T1 tumor cells after different treatments as indicated. Data are presented as mean ± SD (n = 3 independent samples). Statistical significance was determined using one-way ANOVA. Source data are provided as a Source Data file.

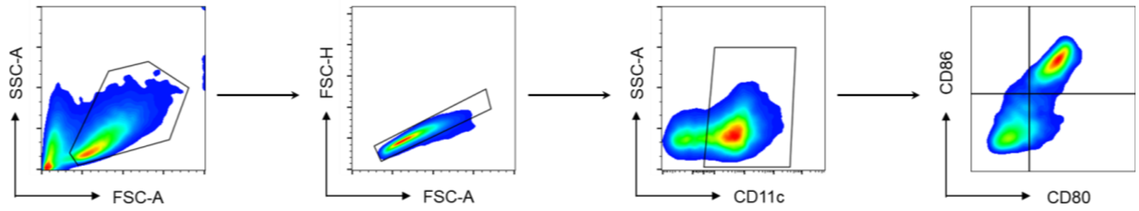

Supplementary Fig. 39 Gating strategy for the flow cytometry analysis of the proportions of BMDC maturation ($CD11c^+CD80^+CD86^+$) in vitro in Fig. 5l and Supplementary Fig. 41-43, and 46e.

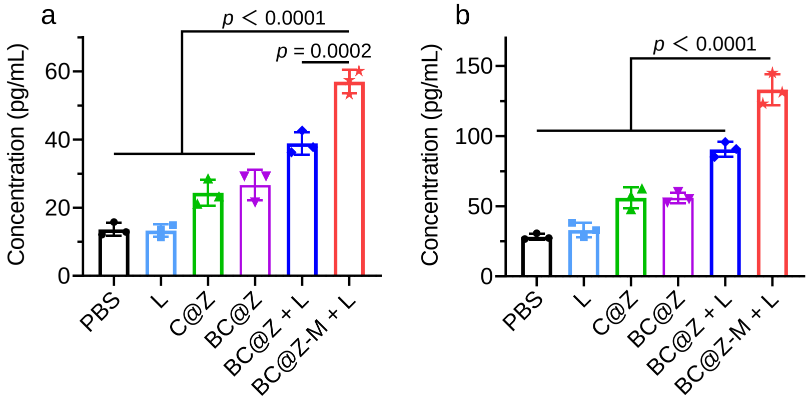

Supplementary Fig. 40 The expression levels of (a) THF-α and (b) IFN-β from BMDCs after different treatments. Data are presented as mean ± SD (n = 3 independent samples). Statistical significance was determined using one-way ANOVA. Source data are provided as a Source Data file.

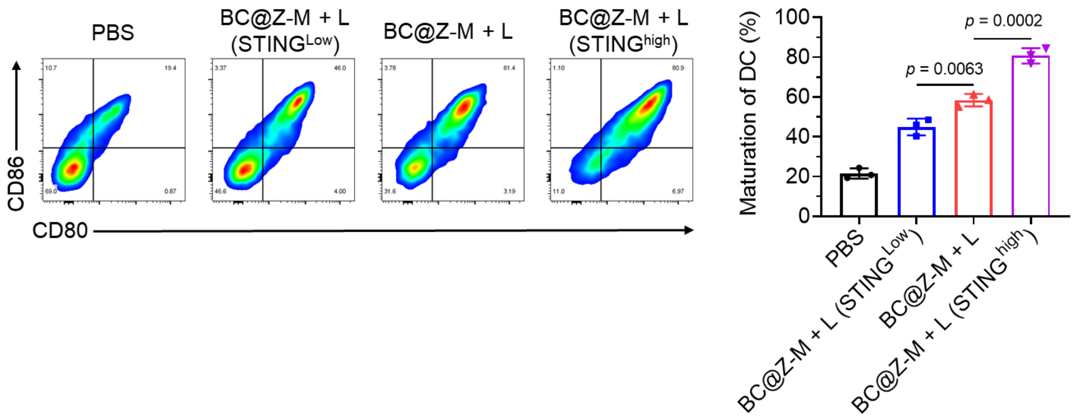

Supplementary Fig. 41 4T1 tumor cells with normal, knockdown, or overexpressed STING expression were treated with "BC@Z-M + L" and subsequently co-cultured with BMDCs in a Transwell system for 24 h. Representative flow cytometry results and quantitative analysis showing the maturation of DCs ($CD80^+$ $CD86^+$) following different treatments. Data are presented as mean ± SD (*n* = 3 independent samples). Statistical significance was determined using one-way ANOVA. Source data are provided as a Source Data file.

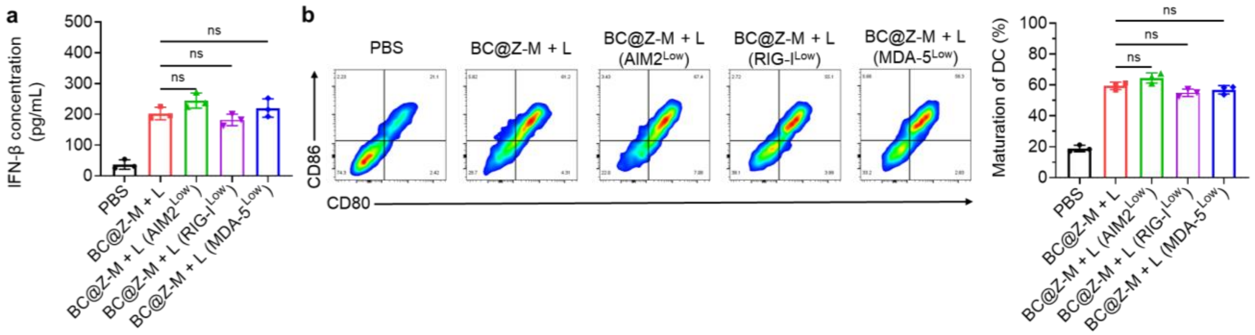

Supplementary Fig. 42 (**a**) 4T1 tumor cells with AIM2, RIG-I, or MDA-5 knockdown were treated with BC@Z-M + L, and IFN-β expression levels in each group were measured using ELISA kit. (**b**) 4T1 tumor cells with AIM2, RIG-I, or MDA-5 knockdown were treated with BC@Z-M + L and subsequently co-cultured with BMDCs in a Transwell system for 24 h. Representative flow cytometry results and quantitative analysis showing the maturation of DCs ($CD80^+CD86^+$) following different treatments. Data are presented as mean ± SD (*n* = 3 independent samples). Statistical significance was determined using one-way ANOVA. Source data are provided as a Source Data file.

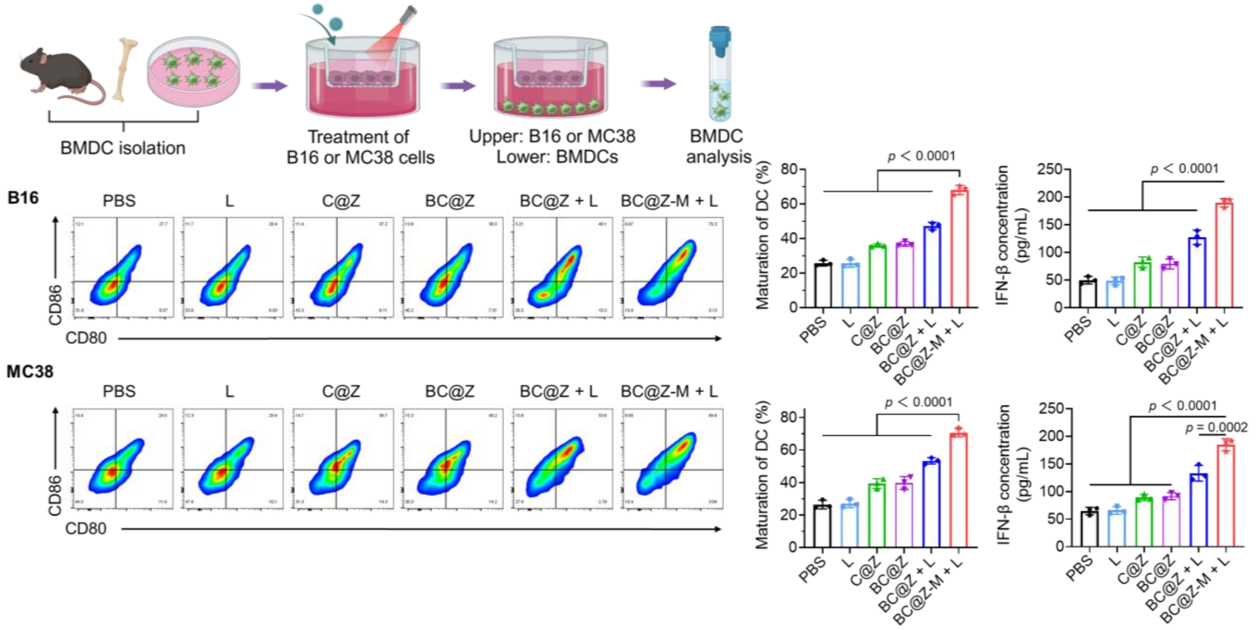

Supplementary Fig. 43 Schematic illustration of the in vitro assessment of BMDC maturation after co-culture with B16 or MC38 cells subjected to various treatments as indicated. Created in BioRender. Li, W. (2024) BioRender.com/d76f921. Representative flow cytometry results and quantitative analysis of mature DCs ($CD80^{+}CD86^{+}$) following different treatments. The expression levels of IFN-β from BMDCs in different groups were also analyzed. Data are presented as mean ± SD (n = 3 independent samples). Statistical significance was determined using one-way ANOVA. Source data are provided as a Source Data file.

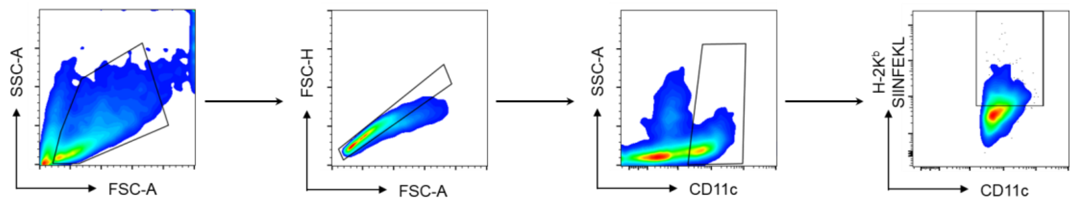

Supplementary Fig. 44 Gating strategy for the flow cytometry analysis of the surface antigen (H-$2K^{b}$/SIINFEKL complex) presentation on BMDCs in Supplementary Fig. 46b.

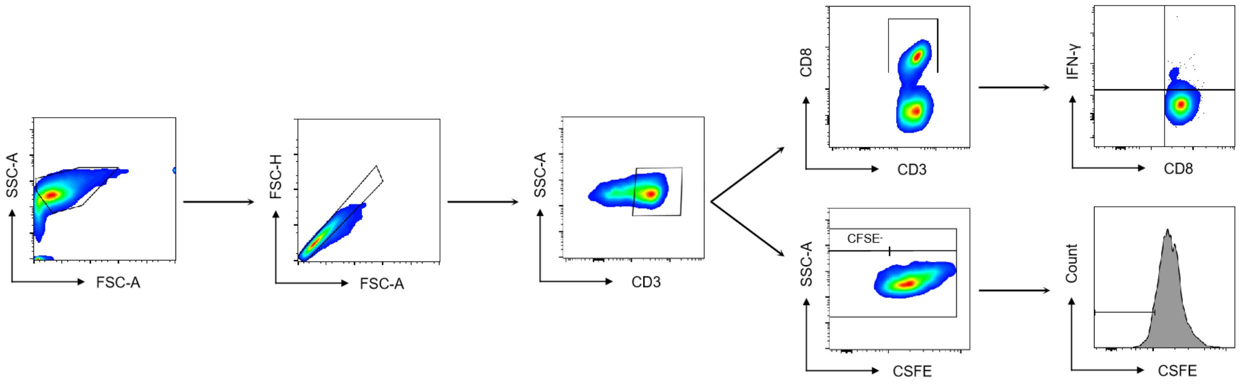

Supplementary Fig. 45 Gating strategy for the flow cytometry analysis of the proportions of IFN-γ^+CD3$^+$CD8$^+$ cells and the proliferation of T cells in Supplementary Fig. 46.

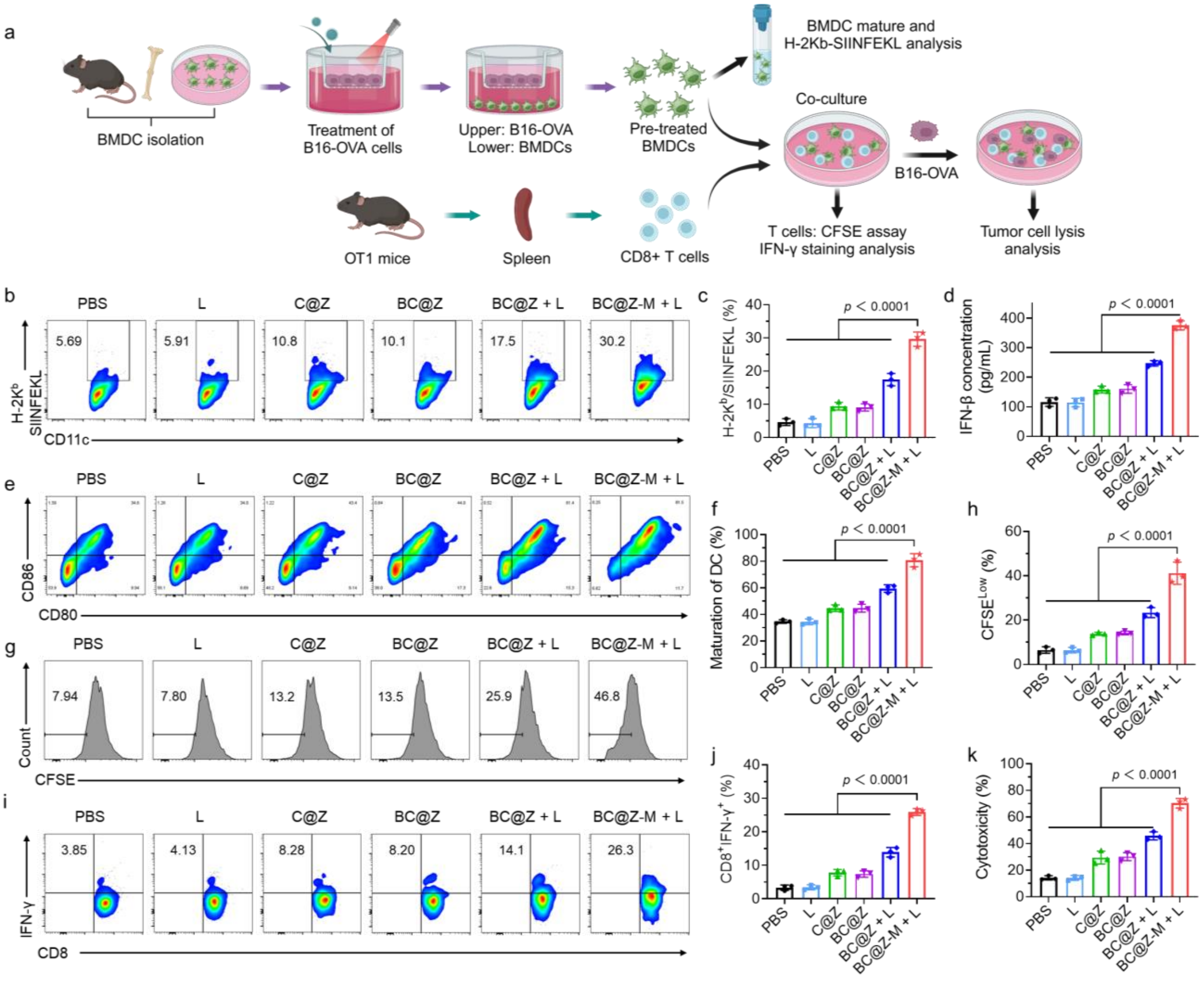

Supplementary Fig. 46 (**a**) Schematic illustration of evaluating the ability of "BC@Z-M + L"-pretreated B16-OVA cells to activate the immune response, including the promotion of DC maturation and antigen cross-presentation, activation of antigen-specific T cells, and enhancement of T cell-mediated killing of tumor cells. Created in BioRender. Li, W. (2023) BioRender.com/i51k615. (**b**) Representative flow cytometry analysis and (**c**) corresponding quantification of surface antigen (H-2Kb/SIINFEKL complex) presentation on BMDCs after co-incubation with B16-OVA cells pretreated with different formulations. (**d**) Expression levels of IFN-β in BMDCs across different treatment groups. (**e**) Representative flow cytometry analysis and (**f**) corresponding quantification of maturation markers (CD80 and CD86) on BMDCs following co-incubation with B16-OVA cells pretreated with various formulations. (**g**) Representative flow cytometry analysis and (**h**) corresponding quantification of CFSE-stained $CD8^+$ T cells in different groups. The

proliferation of OVA-specific $CD8^+$ T cells was assessed through CFSE dilution. (**i**) Representative flow cytometry analysis and (**j**) corresponding quantification of the populations of $CD8^+$ $IFN\gamma^+$ T cells in different groups. (**k**) $CD8^+$ T cell-mediated tumor killing was determined by LDH release using a cytotoxicity detection kit. Data are represented mean ± SD (*n* = 3 independent samples). Statistical analysis was performed using one-way ANOVA. Source data are provided as a Source Data file.

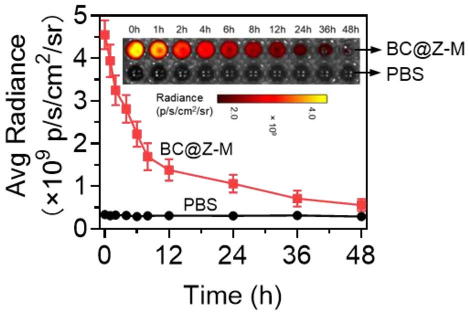

Supplementary Fig. 47 Ex vivo images (inset) and quantified fluorescence intensity of whole blood collected at various time points after intravenous injection with 200 μL of BC@Z-M (1 mg mL^{-1} based on BN-O, red line). The black line indicated the fluorescence intensity of blood from the mice injected with only PBS. Data are presented as mean ± SD (*n* = 3 mice). Source data are provided as a Source Data file.

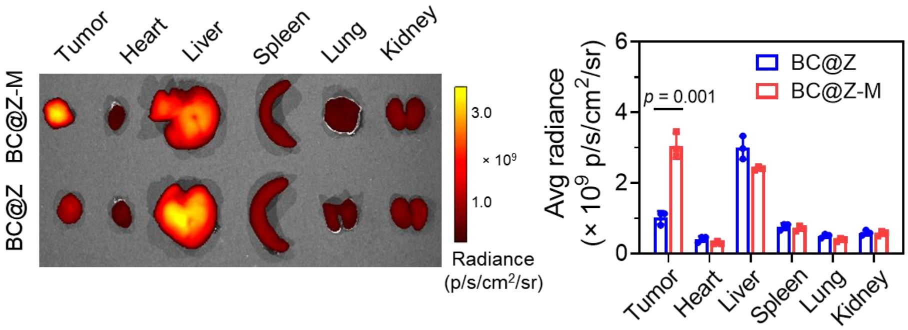

Supplementary Fig. 48 Representative ex vivo fluorescence images of the major organs collected at 24 h after the intravenous injection of DiR-labeled BC@Z or BC@Z-M. Quantification analysis of the fluorescence intensity of different organs. Data are presented as mean ± SD ($n = 3$ mice). Experiment was repeated three times independently with similar results. Source data are provided as a Source Data file.

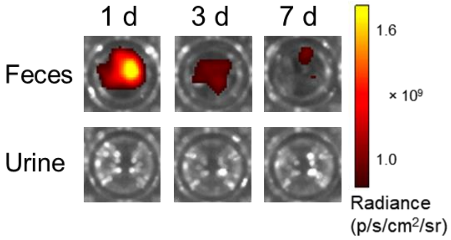

Supplementary Fig. 49 Fluorescence images of the collected feces and urine at various time points after intravenous injection with 200 μL of BC@Z-M (1 mg mL^{-1} based on BN-O) into mice. Experiment was repeated three times independently with similar results.

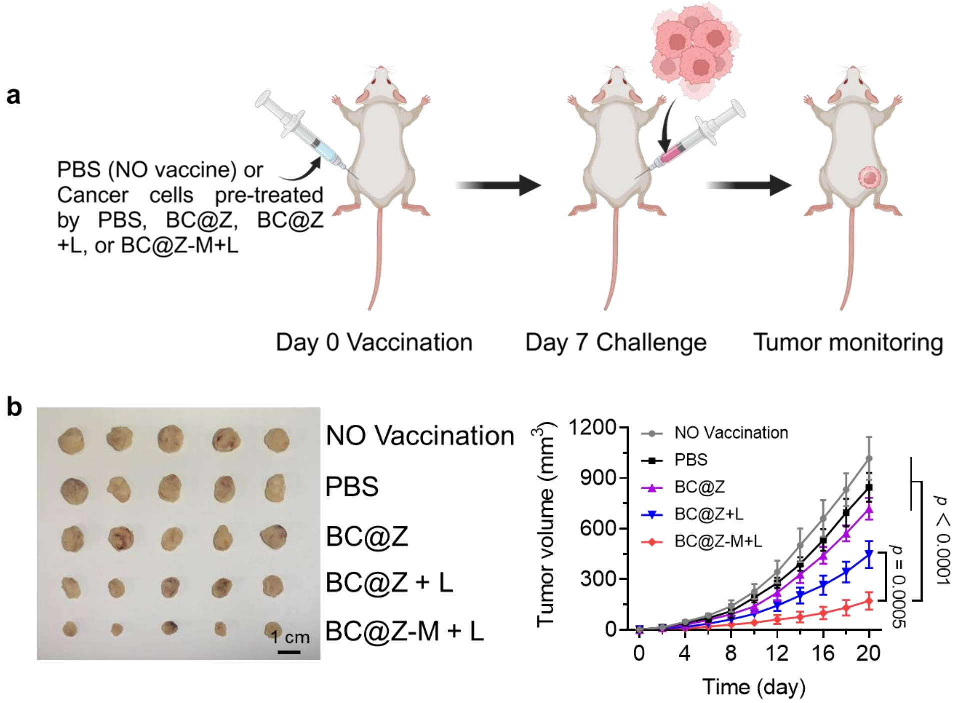

Supplementary Fig. 50 (**a**) Schematic illustration of the in vivo prophylactic vaccination model and experimental timeline. Created in BioRender. Li, W. (2024) BioRender.com/n09m508. (**b**) Photographs and tumor volumes of the re-challenged right tumors in mice that were immunized with either PBS or cancer cells pretreated with different treatments. Data are represented as mean ± SD (*n* = 5 mice). Statistical significance was determined using one-way ANOVA. Source data are provided as a Source Data file.

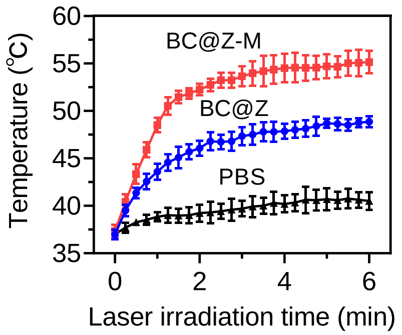

Supplementary Fig. 51 Photothermal curves of tumor sites from mice injected with PBS, BC@Z, and BC@Z-M under the irradiation of 730 nm light (1.0 W cm^{-2}). Data are presented as mean ± SD (*n* = 3 mice). Source data are provided as a Source Data file.

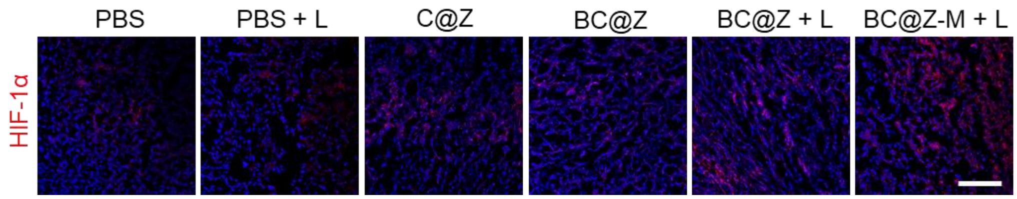

Supplementary Fig. 52 The detection of tumor hypoxia levels following various treatments using HIF-1α staining. The cell nuclei were stained with DAPI (blue fluorescence). Scale bar: 100 μm. Experiment was repeated three times independently with similar results.

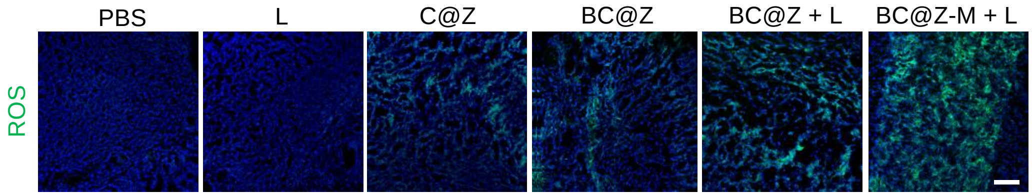

Supplementary Fig. 53 The tumor ROS levels in 4T1 tumor-bearing mice following various treatments detected using DCFH-DA staining. The cell nuclei were stained with DAPI (blue fluorescence). Scale bar: 100 μm. Experiment was repeated three times independently with similar results.

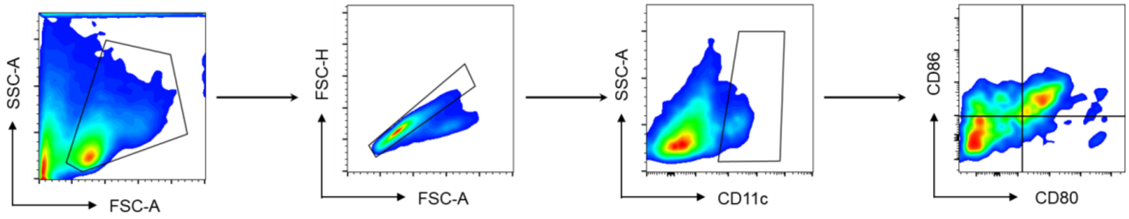

Supplementary Fig. 54 Gating strategy for the flow cytometry analysis of the proportions of DC maturation ($CD11c^{+}CD80^{+}CD86^{+}$) in lymph nodes of 4T1 tumor-bearing mice in Fig. 8f.

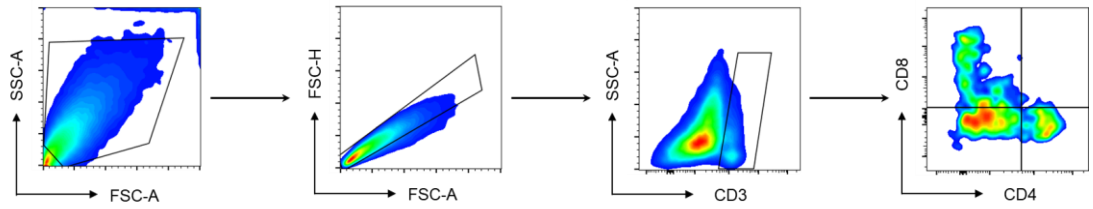

Supplementary Fig. 55 Gating strategy for the flow cytometry analysis of the proportions of tumor-infiltrating CD3$^+$CD4$^+$/CD8$^+$T cells in Fig. 8g and 9e.

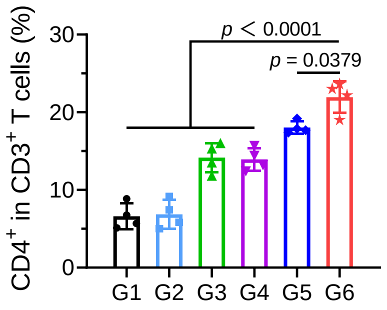

Supplementary Fig. 56 Quantitative analysis of the populations of tumor-infiltrating CD4$^+$ T cells in mice after different treatment according to Fig. 8g. Data are presented as mean ± SD (n = 4 mice). Statistical significance was determined using one-way ANOVA. Source data are provided as a Source Data file.

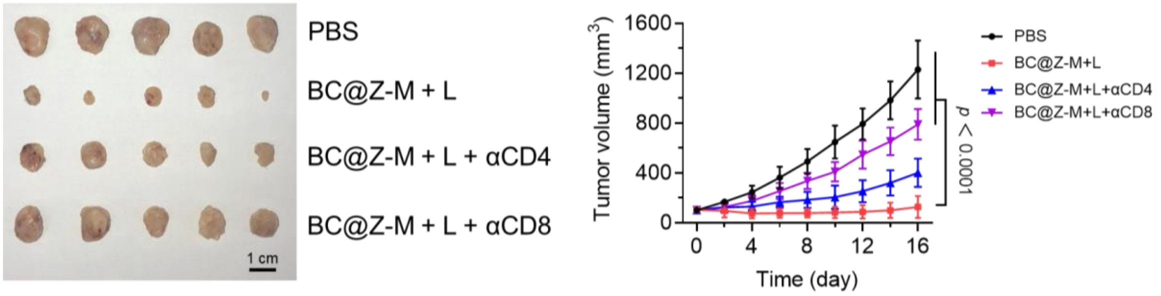

Supplementary Fig. 57 The tumor photographs and tumor volume of 4T1 tumor-bearing mice treated with PBS, BC@Z-M + L, BC@Z-M + L combined with anti-CD4 antibodies, or BC@Z-M + L combined with anti-CD8 antibodies. Data are presented as mean ± SD (n = 5 mice). Statistical significance was determined using one-way ANOVA. Source data are provided as a Source Data file.

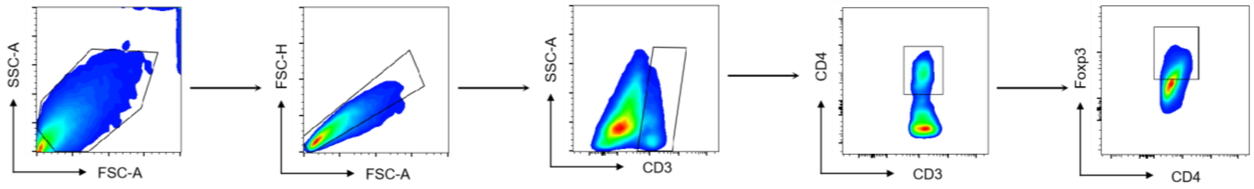

Supplementary Fig. 58 Gating strategy for the flow cytometry analysis of the proportions of tumor-infiltrating $CD3^+CD4^+Foxp3^+$ Tregs in Fig. 8h, 9g.

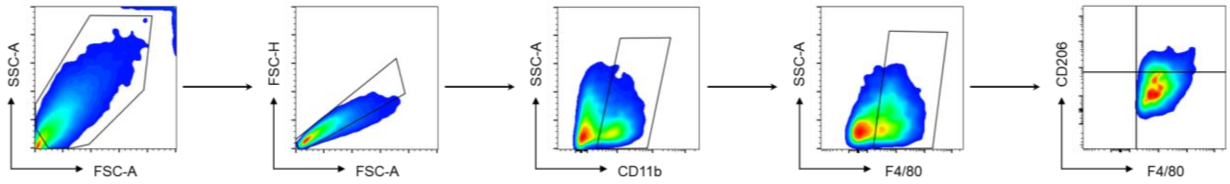

Supplementary Fig. 59 Gating strategy for the flow cytometry analysis of the proportions of M2-like macrophages ($CD11b^+F4/80^+CD206^+$) in tumor in Fig. 8i.

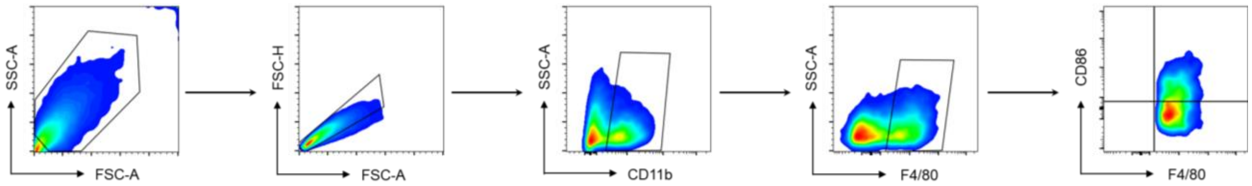

Supplementary Fig. 60 Gating strategy for the flow cytometry analysis of the proportions of M1-like macrophages ($CD11b^+F4/80^+CD86^+$) in tumor in Fig. 8j.

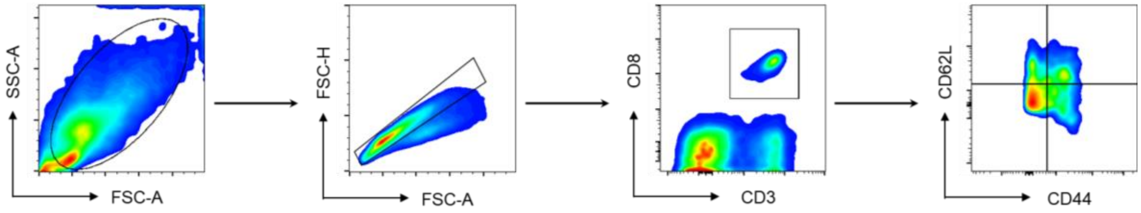

Supplementary Fig. 61 Gating strategy for the flow cytometry analysis of the proportions of $CD3^+CD8^+CD62L^-CD44^+$ effector memory T cells (T_{EM}) in spleen of 4T1 tumor-bearing mice in Fig. 8k, 9i.

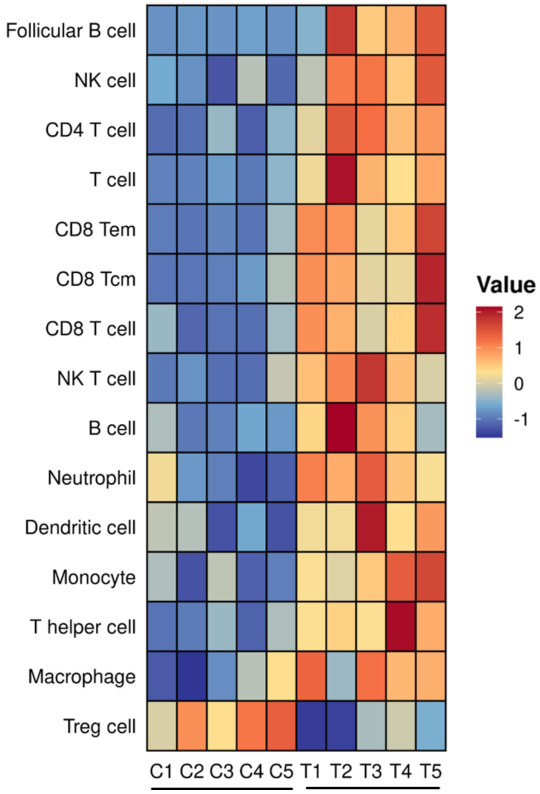

Supplementary Fig. 62 The transcriptomic analyses of various tumor-infiltrating immune cells before (C1-C5 samples) and after (T1-T5 samples) the treatment of BC@Z-M + L. Experiment was repeated three times independently with similar results. Source data are provided as a Source Data file.

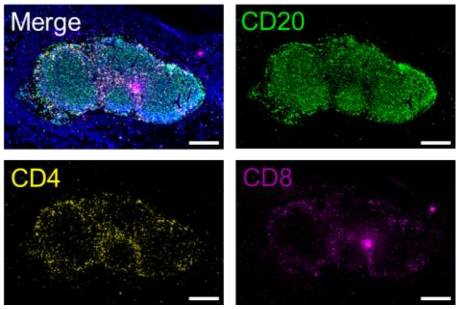

Supplementary Fig. 63 Representative immunofluorescence staining images showing the tertiary lymphoid structures in tumor tissue from the mice treated with “BC@Z-M + L”. Scale bars: 200 μm. Experiment was repeated three times independently with similar results.

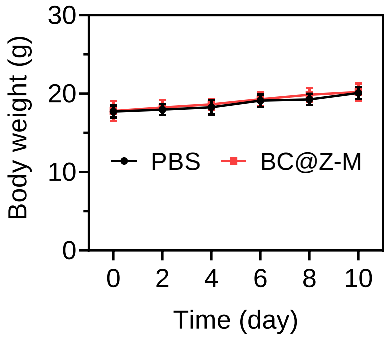

Supplementary Fig. 64 The body weight of healthy mice after the treatment of PBS or BC@Z-M. Data are presented as mean ± SD (n = 3 mice). Source data are provided as a Source Data file.

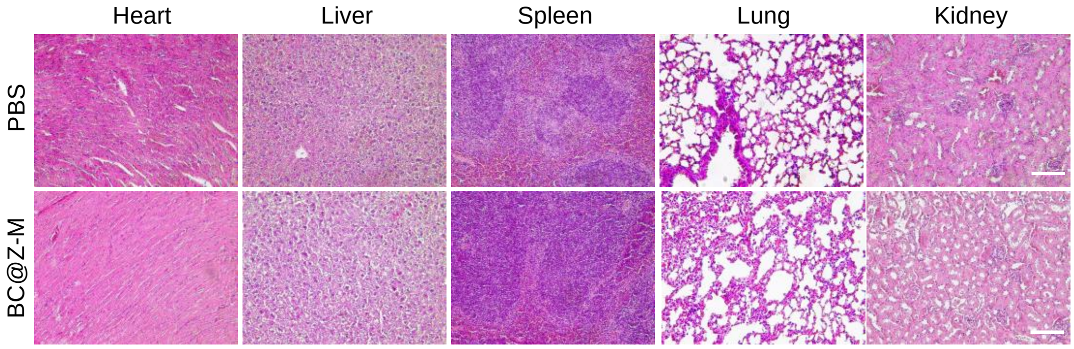

Supplementary Fig. 65 H&E staining of tissue sections from major organs of healthy mice after the treatment of PBS or BC@Z-M. Scale bars: 100 μm. Experiment was repeated three times independently with similar results.

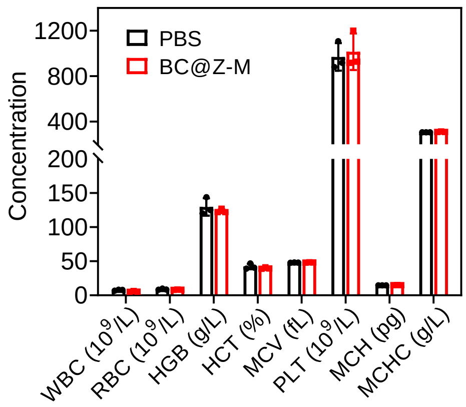

Supplementary Fig. 66 The routine blood test data of the mice after the treatment of PBS or BC@Z-M. The parameters include white blood cell count (WBC), red blood cell count (RBC), hemoglobin (HGB), hematocrit (HCT), mean corpuscular volume (MCV), platelets (PLT), mean corpuscular hemoglobin (MCH), and mean corpuscular concentration (MCHC). Data are presented as mean ± SD (n = 3 mice). Source data are provided as a Source Data file.

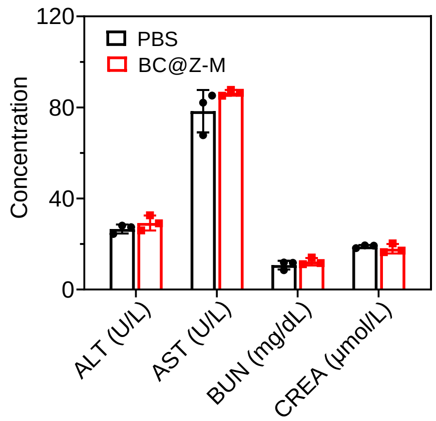

Supplementary Fig. 67 The hepatorenal indicator analysis of the mice with different treatments. The parameters include alanine transaminase (ALT), aspartate transaminase (AST), blood urea nitrogen (BUN), and creatinine (CREA). Data are presented as mean ± SD (n = 3 mice). Source data are provided as a Source Data file.

Supplementary Table 1. Cartesian coordinates of BN calculated by the DFT, B3LYP/6-31G(d), Gaussian 09 program.

atom	x	y	z
C	1.5138815	0.0633831	-0.0505989
C	0.7364594	-1.1227997	-0.0220701
C	-0.7366691	-1.1227171	0.0230319
C	-1.5139467	0.063553	0.0511861
C	-0.7363272	1.2495754	0.0273441
C	0.7363752	1.249502	-0.0269979
C	-2.9772223	0.0652624	0.0998845
C	2.977125	0.0649106	-0.099474
C	3.7419895	1.038676	0.5759104
C	5.1260291	1.0417156	0.5413273
C	5.8532891	0.0752283	-0.2007355
C	5.0841314	-0.9115065	-0.868433
C	3.6986879	-0.9105258	-0.8152918
C	-3.7418607	1.0388229	-0.5760507
C	-5.1259082	1.0419902	-0.5417198
C	-5.8533844	0.0758565	0.2005524
C	-5.0844665	-0.9106206	0.8688396
C	-3.6989864	-0.9097708	0.8159966
N	-7.2351267	0.1022772	0.2838296
N	7.234906	0.1013936	-0.284367
C	7.9070012	-1.041588	-0.9199812
C	7.9949597	0.9187838	0.662914
C	9.4136859	-0.8853119	-1.1000708
C	8.0694236	0.3201541	2.0710574
C	-7.9074331	-1.0401886	0.9201242
C	-7.9947264	0.9176892	-0.6655592
C	-9.4140961	-0.8833952	1.1000277
C	-8.0678236	0.3164614	-2.072668
N	1.2527033	-2.3736554	-0.0176745
S	-0.0002156	-3.4143165	0.0008721
N	-1.2530373	-2.3735279	0.0190632
N	-1.2512489	2.500493	0.066309
S	0.000133	3.5410241	-0.0000924
N	1.2514128	2.5003559	-0.0662283
H	3.2378747	1.7986788	1.1600318
H	5.6437647	1.8033323	1.1093287
H	5.5672097	-1.6922798	-1.4401468

H	3.1604082	-1.6827919	-1.3507489
H	-3.2375585	1.7985728	-1.1603411
H	-5.6434776	1.8035038	-1.1100199
H	-5.5676986	-1.6911417	1.4407622
H	-3.1608862	-1.6818619	1.351888
H	7.7003648	-1.9662907	-0.3581144
H	7.4657559	-1.1740712	-1.9128705
H	8.9990107	1.0550451	0.2614579
H	7.5563608	1.9190024	0.6923572
H	9.7800819	-1.7432196	-1.6727198
H	9.6603676	0.0227704	-1.6599262
H	9.9546394	-0.8689613	-0.1493958
H	8.6412249	0.9769504	2.7358466
H	7.0687085	0.1881793	2.4947828
H	8.561623	-0.6584124	2.0517035
H	-7.7009864	-1.9653589	0.3589328
H	-7.4662618	-1.1720303	1.9131179
H	-8.9991354	1.0543192	-0.2651116
H	-7.5565037	1.9180177	-0.6965063
H	-9.7807071	-1.7405304	1.6736935
H	-9.66055	0.0254338	1.6587781
H	-9.9550373	-0.8680541	0.1493274
H	-8.6393814	0.9717782	-2.739123
H	-7.066689	0.1841953	-2.4953178
H	-8.5595915	-0.6622948	-2.0519355

Supplementary Table 2. Cartesian coordinates of BN-O calculated by the DFT, B3LYP/6-31G(d), Gaussian 09 program.

atom	x	y	z
C	1.5036064	-0.0562564	0.0129708
C	0.7329212	-1.2403797	0.0225825
C	-0.737629	-1.2371713	0.0414146
C	-1.5027671	-0.0495255	0.0489365
C	-0.732109	1.1345541	0.0420592
C	0.7383684	1.1313979	0.0256281
C	-2.9754105	-0.0463935	0.0592991
C	2.9760519	-0.0599889	-0.0141884
C	3.7145838	0.8544916	0.7592481
C	5.1055396	0.8542763	0.7345589
C	5.7766492	-0.0611767	-0.0771355
C	5.0726444	-0.9716482	-0.8519902
C	3.6810021	-0.9731382	-0.8173423
C	-3.6984696	0.8687924	-0.7276141
C	-5.0895886	0.8715372	-0.7273721
C	-5.776754	-0.0433247	0.0712776
C	-5.08811	-0.9534178	0.8605609
C	-3.695996	-0.9568886	0.8514319
N	-7.2857083	-0.0825053	0.1103268
N	7.2840023	-0.0992687	-0.1467595
C	7.7338754	1.3113227	-0.5837153
C	7.756329	-0.4701546	1.2659931
C	9.1263743	1.3501189	-1.1843575
C	9.2570731	-0.6587761	1.3819692
C	-7.723515	1.2885614	0.649022
C	-7.7400919	-0.3536138	-1.3373855
C	-9.210883	1.3958723	0.9294284
C	-9.1870929	-0.7941095	-1.4575496
N	1.2485488	-2.4932891	0.0293199
S	-0.0074371	-3.5247441	0.0313377
N	-1.2588686	-2.4877328	0.0337664
N	-1.2476311	2.3873399	0.0629343
S	0.0079254	3.419081	0.0369822
N	1.2590937	2.3820737	0.0078771
O	-7.7357349	-1.063504	0.9338031
O	7.717337	-1.0341866	-1.0309083
H	3.196074	1.5638964	1.3918956

H	5.6432973	1.5683473	1.3481052
H	5.6416081	-1.651947	-1.4710209
H	3.1342786	-1.6806022	-1.4285826
H	-3.1675745	1.5753782	-1.3531468
H	-5.6141333	1.5826187	-1.3554256
H	-5.6693032	-1.6363881	1.4651821
H	-3.1614144	-1.6654979	1.4720529
H	7.0030885	1.6052931	-1.3388461
H	7.6292898	1.9780568	0.2753468
H	7.2370129	-1.4108187	1.4565803
H	7.3845619	0.2835356	1.963603
H	9.2692779	2.3329943	-1.6459613
H	9.9167287	1.205092	-0.4471769
H	9.2076613	0.5771819	-1.9490642
H	9.4679249	-1.2150306	2.3009997
H	9.7950125	0.2902718	1.4376405
H	9.6176228	-1.2363091	0.5288253
H	-7.1572178	1.376808	1.57771
H	-7.3783233	2.0584757	-0.0445077
H	-7.0819273	-1.160026	-1.6650473
H	-7.5232569	0.5349991	-1.934325
H	-9.3762061	2.2392333	1.6076506
H	-9.798405	1.5753347	0.0269528
H	-9.5529452	0.4797548	1.4140407
H	-9.3304077	-1.2315272	-2.4510819
H	-9.8956959	0.0278233	-1.3478125
H	-9.3929274	-1.5509056	-0.6994999
