# Supplementary material for: A hypoxia-activated and microenvironment-remodeling nanoplatform for multifunctional imaging and potentiated immunotherapy of cancer
Source: Nat Commun. 2024 Nov 29;15:10395. doi: 10.1038/s41467-024-53906-x (PMC11607447; doi:10.1038/s41467-024-53906-x)
Supplement: Supplementary file 2 — Description of Additional Supplementary Files [file 41467_2024_53906_MOESM2_ESM.pdf]

Description of Additional Supplementary Files

Supplementary Data 1. The sequences for plasmid vectors and shRNAs used in this study.
