# Supplementary material for: A hypoxia-activated and microenvironment-remodeling nanoplatform for multifunctional imaging and potentiated immunotherapy of cancer
Source: Nat Commun. 2024 Nov 29;15:10395. doi: 10.1038/s41467-024-53906-x (PMC11607447; doi:10.1038/s41467-024-53906-x)
Supplement: Supplementary file 4 — Reporting Summary [file 41467_2024_53906_MOESM4_ESM.pdf]

nature portfolio

Corresponding author(s): Ji Qi

Last updated by author(s): Oct 15, 2024

Reporting Summary

Nature Portfolio wishes to improve the reproducibility of the work that we publish. This form provides structure for consistency and transparency in reporting. For further information on Nature Portfolio policies, see our Editorial Policies and the Editorial Policy Checklist.

Statistics

For all statistical analyses, confirm that the following items are present in the figure legend, table legend, main text, or Methods section.

n/a	Confirmed	
☐	☒	The exact sample size (*n*) for each experimental group/condition, given as a discrete number and unit of measurement
☐	☒	A statement on whether measurements were taken from distinct samples or whether the same sample was measured repeatedly
☐	☒	The statistical test(s) used AND whether they are one- or two-sided<br>*Only common tests should be described solely by name; describe more complex techniques in the Methods section.*
☒	☐	A description of all covariates tested
☐	☒	A description of any assumptions or corrections, such as tests of normality and adjustment for multiple comparisons
☐	☒	A full description of the statistical parameters including central tendency (e.g. means) or other basic estimates (e.g. regression coefficient) AND variation (e.g. standard deviation) or associated estimates of uncertainty (e.g. confidence intervals)
☐	☒	For null hypothesis testing, the test statistic (e.g. *F*, *t*, *r*) with confidence intervals, effect sizes, degrees of freedom and *P* value noted<br>*Give P values as exact values whenever suitable.*
☒	☐	For Bayesian analysis, information on the choice of priors and Markov chain Monte Carlo settings
☒	☐	For hierarchical and complex designs, identification of the appropriate level for tests and full reporting of outcomes
☒	☐	Estimates of effect sizes (e.g. Cohen's *d*, Pearson's *r*), indicating how they were calculated

Our web collection on statistics for biologists contains articles on many of the points above.

Software and code

Policy information about availability of computer code

Data collection	NMR spectra: Bruker-DPX 400 spectrometer; HRMS: Varian 7.0T FTMS Mass Spectrometer System; DLS: Malvern Zetasizer Nano-ZS; TEM: FEI Talos L120C G2; UV-vis: Shimadzu UV-3600 plus spectrometer; FL: Edinburgh FLS1000 spectrometer; Laser: Changchun Laser Optical 730; DFT: Gaussian 09 program package (revision D. 01) at the level of B3LYP/6-31G*; Thermal imaging camera: FLK-Ti200; Confocal imaging: LSM 800 with Airyscan; Microplate reader: spark spectrometer; In-vivo imaging: PerkinElmer, IVIS Spectrum; PA imaging: Vevo® LAZR-X, Fuji VisualSonics; In-vivo Imaging: NightOWL II LB983 and in vivo NIR-II fluorescence imaging system; Flow cytometry: FACSAria III.
Data analysis	MestReNova (version 14.0); GaussView (version 5.0); DLS: Zetasizer Software 7.12; Graphpad Prism (version 8.0.2); Image J2x (version 2.1.4.7); FlowJo (version 10.0); PA image: Vevo LAB 5.6.1; ZEN 2012 (version 1.1.2.0).

For manuscripts utilizing custom algorithms or software that are central to the research but not yet described in published literature, software must be made available to editors and reviewers. We strongly encourage code deposition in a community repository (e.g. GitHub). See the Nature Portfolio guidelines for submitting code & software for further information.

Data

Policy information about availability of data

All manuscripts must include a data availability statement. This statement should provide the following information, where applicable:

- Accession codes, unique identifiers, or web links for publicly available datasets
- A description of any restrictions on data availability
- For clinical datasets or third party data, please ensure that the statement adheres to our policy

The data that support the findings of this study are available within the Article, Supplementary Information, or Source Data file. The RNAseq data used in this study are available in the Gene Expression Omnibus (GEO) database with accession number GSE278201.

Research involving human participants, their data, or biological material

Policy information about studies with human participants or human data. See also policy information about sex, gender (identity/presentation), and sexual orientation and race, ethnicity and racism.

Reporting on sex and gender	not applicable
Reporting on race, ethnicity, or other socially relevant groupings	not applicable
Population characteristics	not applicable
Recruitment	not applicable
Ethics oversight	not applicable

Note that full information on the approval of the study protocol must also be provided in the manuscript.

Field-specific reporting

Please select the one below that is the best fit for your research. If you are not sure, read the appropriate sections before making your selection.

- [x] Life sciences
- [ ] Behavioural & social sciences
- [ ] Ecological, evolutionary & environmental sciences

For a reference copy of the document with all sections, see nature.com/documents/nr-reporting-summary-flat.pdf

Life sciences study design

All studies must disclose on these points even when the disclosure is negative.

Sample size	Sample size estimates have been performed on previous experience to obtain statistical significance and reproducibility. For in vitro experiments and biodistribution, n = 3 was selected in order to measure the distribution closer to the true distribution on the premise of cost saving. For in vivo antitumor study and assessment of immune responses, each group contained at least 4 (n ≥ 4) for evaluating the statistical significance. A precise value of 'n' was provided in the legends of figures. Sample size was chosen in consideration of animal individual differences and the 4R principle for the credibility of the results.
Data exclusions	No data were excluded from the analyses.
Replication	All experiments underlying main conclusions of this study have been successfully replicated multiple times and corroborated by several models. Experiment repeat numbers are reported in the figure legends.
Randomization	All samples/organisms were randomly allocated into experimental groups.
Blinding	The investigators were blinded to group allocation during data collection and analysis.

Reporting for specific materials, systems and methods

We require information from authors about some types of materials, experimental systems and methods used in many studies. Here, indicate whether each material, system or method listed is relevant to your study. If you are not sure if a list item applies to your research, read the appropriate section before selecting a response.

Materials & experimental systems

n/a	Involved in the study	
	☒	Antibodies
	☒	Eukaryotic cell lines
☒		Palaeontology and archaeology
	☒	Animals and other organisms
☒		Clinical data
☒		Dual use research of concern
☒		Plants

Methods

n/a	Involved in the study	
☒		ChIP-seq
	☒	Flow cytometry
☒		MRI-based neuroimaging

Antibodies

Antibodies used

The following antibodies were used for western blot. They are listed as antigen first, followed by supplier, catalog number and clone/lot number as applicable.
1) Anti-mouse CD86 antibody (Cell Signaling Technology, Rabbit mAb, #19589, 1:1000 dilution)
2) Recombinant Anti-mouse iNOS antibody (Abcam, Rabbit mAb, #ab178945, 1: 1000 dilution)
3) Anti-mouse Na,K-ATPase Antibody (Cell Signaling Technology, #3010 ,1:1000 dilution)
4) Recombinant Anti-mouse Integrin alpha 4/CD49D (Abcam, Rabbit mAb, #ab75760, 1:1000 dilution)
5) Recombinant Anti-mouse Integrin beta 1 (Abcam, Rabbit mAb, #ab179471, 1: 1000 dilution)
6) Anti-mouse phospho-TBK1/NAK (Ser172) (D52C2) (Cell Signaling Technology, #5483, 1:1000 dilution)
7) Anti-mouse TBK1/NAK (EP611Y) (Abcam, ab40676, 1:1000 dilution)
8) Anti-mouse phospho-IRF3 (Ser396) (D6O1M) (Cell Signaling Technology, #29047, 1:1000 dilution)
9) Anti-mouse IRF-3 (EPR2418Y) (Abcam, ab68481, 1:1000 dilution)
10) Anti-mouse phospho-STING (Ser365) (D8F4W) (Cell Signaling Technology, #72971, 1:1000 dilution)
11) Anti-mouse STING (EPR25090-107) (Abcam, ab288157, 1:1000 dilution)
12) Anti-mouse GAPDH (Proteintech, #10494-1-AP, 1:10,000 dilution)
13) Anti-mouse CD47 antibody (ABclonal, #A1838, 1:1000 dilution)
14) Anti-mouse MDA-5 antibody (Cell Signaling Technology, #5321, 1:1000 dilution)
15) Anti-mouse RIG-I antibody (Cell Signaling Technology, #3743, 1:1000 dilution)
16) Anti-mouse AIM2 antibody (Absin, abs125828, 1:1000 dilution)
17) Anti-mouse β-actin antibody (Cell Signaling Technology, #4967S, 1:1000 dilution)

The following antibodies were used for immunofluorescence staining. They are listed as antigen first, followed by supplier, catalog number and clone/lot number as applicable.
1) Recombinant Anti-mouse CD31 antibody (Abcam, Rabbit mAb, #ab222783, 1:200 dilution)
2) Recombinant Anti-mouse Calreticulin antibody (Abcam, Rabbit mAb, #ab92516, 1:200 dilution)
3) Recombinant Anti-mouse HMGB1 antibody (Abcam, Rabbit mAb, #ab79823, 1:200 dilution)
4) Anti-mouse HIF-1α Antibody (Cohesion, CPA3239, 1:200 dilution)
5) Goat Anti-Rabbit IgG H&L (Alexa Fluor® 488) (Abcam, #ab150077, 1:1000 dilution)
6) Donkey Anti-Rabbit IgG H&L (Alexa Fluor® 647) (Abcam, #ab150075, 1:1000 dilution)

The following antibodies were used for flow cytometry. They are listed as antigen first, followed by supplier, catalog number and clone/lot number as applicable.
1) FITC anti-mouse CD11c Antibody (Biolegend, #117306, 1:200 dilution)
2) PE anti-mouse CD80 Antibody (Biolegend, #104707, 1:200 dilution)
3) APC anti-mouse CD86 Antibody (Biolegend, #105011, 1:200 dilution)
4) FITC anti-mouse CD3 Antibody (Biolegend, #100203, 1:200 dilution)
5) Brilliant Violet 421™ anti-mouse CD4 Antibody (Biolegend, #100543, 1:200 dilution)
6) APC anti-mouse CD8a Antibody (Biolegend, #100711, 1:200 dilution)
7) PE anti-mouse Foxp3 Antibody (Biolegend, #320007, 1:200 dilution)
8) PE anti-mouse F4/80 Antibody (Biolegend, #123109, 1:200 dilution)
9) FITC anti-mouse CD11b Antibody (Biolegend, #101205, 1:200 dilution)
10) Brilliant Violet 421™ anti-mouse CD206 Antibody (Biolegend, #141717, 1:200 dilution)
11) PE anti-mouse CD44 Antibody (Biolegend, #103007, 1:200 dilution)
12) Brilliant Violet 605™ anti-mouse CD62L Antibody (Biolegend, #104437, 1:200 dilution)
13) PE/Cyanine7 anti-mouse H-2Kb bound to SIINFEKL Antibody (Biolegend, #141607, 1:200 dilution)
14) PE anti-mouse IFN-γ Antibody (Biolegend, #505808, 1:200 dilution)
15) APC/Cyanine7 anti-mouse CD3 Antidody (Biolegend, #100221, 1:200 dilution)

Validation	For all the antibodies, we carried out western blot according to the method on the company's website, and detected whether the band size met the expectation with molecular weight marker, and added appropriate positive control and negative control. Antibodies purchased from Cell Signaling Technology and Abcam were validated as per their website stating "Antibody signal is measured in model systems with known presence/absence of target signal. Besides, each antibody's manual contains authentic data results from the companies (Biolegend, Abcam, Cell Signaling Technology, and Proteintech) validating specificity, and our data also verifies the corresponding antibody's specificity.

Eukaryotic cell lines

Policy information about cell lines and Sex and Gender in Research

Cell line source(s)	4T1 (Catalog #: TCM 32), RAW264.7 (Catalog #: TCM 13) and NIH 3T3 cells (Catalog #: GNM 6) were purchased from Cell Bank of Shanghai, Chinese Academy of Sciences (Shanghai, China). HUVECs (Catalog #: HUVEC-20001) were purchased from Cyagen Biosciences (Guangzhou, China). All cell lines used in this study were tested mycoplasma free.
Authentication	The cell lines were certified by the manufacturers (surface markers, morphology).
Mycoplasma contamination	Cells were routinely screened for free of Mycoplasma contaminations. All cell lines are Mycoplasma negative with this study.
Commonly misidentified lines (See ICLAC register)	No commonly misidentified cell lines were used in the study.

Animals and other research organisms

Policy information about studies involving animals; ARRIVE guidelines recommended for reporting animal research, and Sex and Gender in Research

Laboratory animals	Female BALB/c mice (6-8 weeks old) were purchased from Laboratory Animal Center of the Academy of Military Medical Sciences (Beijing, China). All procedures involving animals were conducted in accordance with the guidelines set by the Tianjin Committee of Use and Care of Laboratory Animals, and approved by the Animal Ethics Committee of Nankai University. All mice were cultured in suitable temperature and humidity environment (25 ℃, suitable humidity (typically 50%), 12 hour dark/light cycle), and fed with sufficient water and food.
Wild animals	The study did not involve wild animals.
Reporting on sex	Because the selected model was breast cancer, female animals were selected for experiments. Although we have used single-sex animals in our research, we think that the research results were not only applicable to single sex.
Field-collected samples	The study did not involve samples collected from the field.
Ethics oversight	All animal studies were conducted under the guidelines set by Tianjin Committee of Use and Care of Laboratory Animals, and the overall project protocols were approved by the Animal Ethics Committee of Nankai University.

Note that full information on the approval of the study protocol must also be provided in the manuscript.

Plants

Seed stocks	not applicable
Novel plant genotypes	not applicable
Authentication	not applicable

Flow Cytometry

Plots

Confirm that:

- [x] The axis labels state the marker and fluorochrome used (e.g. CD4-FITC).
- [x] The axis scales are clearly visible. Include numbers along axes only for bottom left plot of group (a 'group' is an analysis of identical markers).
- [x] All plots are contour plots with outliers or pseudocolor plots.
- [x] A numerical value for number of cells or percentage (with statistics) is provided.

Methodology

Sample preparation	For tissue sample, the tissue was first mechanically disrupted from mice and divided into small pieces and homogenized in cold staining buffer to form single cell suspensions in the presence of digestive enzyme.
Instrument	BD FACSAria III
Software	Data analysis: FlowJo version 10.0
Cell population abundance	No cell sorting was performed.
Gating strategy	Gating was first based on FSC/SSC and singlet cells were gated for further analysis. The cell populations were then analyzed based on expression of markers. Gating was then based on positive level.

- [x] Tick this box to confirm that a figure exemplifying the gating strategy is provided in the Supplementary Information.
