# Supplementary material for: A hypoxia-activated and microenvironment-remodeling nanoplatform for multifunctional imaging and potentiated immunotherapy of cancer
Source: Nat Commun. 2024 Nov 29;15:10395. doi: 10.1038/s41467-024-53906-x (PMC11607447; doi:10.1038/s41467-024-53906-x)
Supplement: Supplementary file 5 — Source Data [file 41467_2024_53906_MOESM5_ESM.pdf]

nature portfolio

Peer Review File

A hypoxia-activated and microenvironment-remodeling nanoplatform for multifunctional imaging and potentiated immunotherapy of cancer

Corresponding Author: Professor Ji Qi

Parts of this Peer Review File have been redacted as indicated to remove third-party material.

This file contains all reviewer reports in order by version, followed by all author rebuttals in order by version.

Version 0:

Reviewer comments:

Reviewer #1

(Remarks to the Author)
In the manuscript "Hypoxia-activated and tumor microenvironment remodeling-amplified nanoplatforms for multifunctional imaging and potentiated immunotherapy of cancer" (#NCOMMS-24-24735-T), Song and colleagues describe a hypoxia-activatable theranostic platform able to induce tumor-selective immunogenic cell death. The message of the article is important, novel and within scope for Nature Communications. Findings are abundant, technically diverse and well presented, and this reviewer had NO concerns about image manipulation or data fabrication. Globally, the authors must be congratulated on the abundant amount of work and quality of presentation. However, some of the conclusions, are not supported by the dataset, and hence must be appropriately revisited (or additional experiments performed in this respect). Alongside, a few experiments are missing that would considerably strengthen the paper, and some minor issues emerged that need to be addressed, as detailed here below. In summary, this reviewer believes the article by Song and colleagues represents a strong candidate for publication in Nature Communications upon revision.
Major points:
1) The authors interpret their dataset in several instances as to suggest that STING-IFN-I signaling is responsible for ICD after combined BC@Z-M+L treatment, and openly suggest in the discussion that “The hypoxia-triggered and TME remodeling boosted PTT/PDT attributes jointly activated the ICD and cGAS-STING pathway, engaging both the innate and adaptive immune system to bolster tumor-specific T-cell immune responses and alleviate immunological resistance”. However, the evidences for the cGAS-STING role are circumstantial. The authors show through western blot analysis the patterns of phosphorylation of STING and downstream TBK1 and IRF3 factors, which is indicative but not demonstrative. I recommend to perform in vitro experiments in the gain (overexpression in parental cells) and loss (silencing by shRNAs or knockout by CRIPSR/Cas9) of either STING or IFNAR to show whether STING-IFN-I axis really regulates the immunogenicity of the proposed therapeutic approach.

2) The authors show γ-H2AX-related DNA damage after diverse treatments and pose it as the rationale to investigate STING pathway. Have they excluded the effects of other DNA (such AIM2) or even RNA (RIG-I, MDA-5) sensors?

3) According to the author model, BC@Z-M+L is able to induce direct cancer cell killing while activating anticancer immune responses (ICD), which they demonstrate by analyzing the exposure/release of diverse alarmirs (calreticulin, HMGB1, ATP and IFNs-I). Alongside, they propose the ability of these alarmins to activate dendritic cells by flow cytometry. Are then, these activated dendritic cells able to phagocytose apoptotic cancer cells and to cross-present their antigens to T cells? Are then, these activated T-cells able to kill neighboring cancer cells? The authors should test the immune activation and thus the real immunogenicity of cancer cell death either by in vitro reproducing the crucial steps of the cancer-immunity cycle, or by in vivo vaccinating mice with cancer cells pre-treated with BC@Z, BC@Z+L, BC@Z-M+L or no vaccine and challenging mice with parental cells. Also, it would be great to see the effect in NSG mice, too, to check for a real involvement of the adaptive immune system. Even better, CD4/CD8 depletion should be implemented in immunocompetent mice, as NSG are highly immunodeficient.

4) It would be great to see a spatial proteomic or even transcriptomic analysis of tumor biopsies to evaluate the type of

infiltrating immune cells (pre and post treatment). Also, it would be great to see tertiary lymphoid structures after treatment, but this reviewer acknowledges it may be beyond the scope of the current investigation.
5) The number of experimental replicates for in vitro and in vivo studies is missing. Please provide it.

6) The authors use 4T1 breast carcinoma cells as the sole cancer model, a highly used triple negative breast cancer model, known to be responsive to immunotherapy. To exclude that their observations are related to the model used, at least in vitro experiments should be performed in other tumor models, maybe of a different background (C57BL/6 mice for example) y comparing polyclonal with monoclonal systems (as the OVA-OT-1).

Minor points:
1. Figure 1 is too complicated for an original article and is more appropriate for commentary-like pieces. Please replace with a more schematic picture.

Reviewer #2

(Remarks to the Author)
This manuscript introduces a hypoxia-activatable nanotheranostic system with NIR-II fluorescence/PA imaging, PDT/PTT properties, and multifunctional photoimmunotherapy for cancer treatment. The modification of a vascular disrupting agent induces remodeling of the tumor microenvironment, thus amplifying the hypoxia-responsive phototheranostics. Moreover, the probe enables activatable NIR-II fluorescence and PA imaging for tumor delineation, achieving high therapeutic efficacy and immune activation effects in multiple tumor models. Overall, the work is interesting, and the activatable multifunctional theranostic nanosystem demonstrates high performance in both molecular imaging and immunotherapy. Therefore, it can be considered acceptance in Nature Communications after addressing the following issues.
1. The authors verified the conversion of BN-O into BN in molecular state. Please provide further evidence to demonstrate whether the BN-O encapsulated in BC@Z-M can undergo conversion to BN in hypoxic environment.
2. While the authors indicated that BC@Z-M could efficiently kill tumor cells under light irradiation (Figure 4j), further investigation is needed to examine the extent of tumor cell death and apoptosis following various treatments.
3. The intracellular DNA oxidation plays an important role in the activatable PDT/PTT-mediated STING activation. Although the authors studied the mitochondrial damage and DNA damage (Figure 5d-f), the intracellular DNA oxidation was not evaluated.
4. Immunofluorescence staining was utilized to analyze HMGB1 release from cells undergoing ICD, but it did not sufficiently demonstrate the release of HMGB1 into extracellular space. Therefore, I suggest the authors to evaluate the HMGB1 release using ELISA.
5. The authors studied the signal of BC@Z-M in major organs under NIR-II fluorescence imaging (Fig. 6f). However, the NIR-II fluorescence signal is activated in hypoxia conditions, thus the NIR-II signal in different tissues may not reflect their real levels. Therefore, the authors are suggested to measure the organ distribution of the nanoagent by other method.
6. The authors conducted MTT assay to investigate the cytotoxicity to tumor cells. But how about the biocompatibility of the nanoagent to normal cells? Please supplement some other tests.
7. Since BN-O is a responsive molecule, and the Synthesis Methods section of Supplementary Material didn't mention whether the synthesis of BN-O was carried out under aerobic or hypoxic conditions, please provide the concentration of oxygen at which BN-O was synthesized.
8. How about the photothermal conversion efficiency of BC@Z-M after hypoxia treatment? Please provide additional data regarding this.
9. Please provide references to support the following statement: "... coupled with the release of Zn^{2+} from the nanocarrier, augmented the activation of STING pathway ...".
10. In biosafety evaluation, it is essential to monitor the mice body weight changes after the injection of BC@Z-M into healthy mice.

Reviewer #3

(Remarks to the Author)
Qi et al. developed a tumor microenvironment-responsive nanoplatform that integrated hypoxia-activatable multimode bioimaging and immunotherapy for the diagnosis and treatment of cancer. In this system, the hypoxia-responsive phototheranostic features of the probe enabled sensitive diagnosis and on-demand treatment, and the vascular disrupting agent further amplified the properties. The turn-on NIR II FL/PA imaging combined with the ICD/STING co-activated immunotherapy were finally achieved.
Given the novelty and comprehensive investigation in this work, I would recommend the acceptance of this manuscript in Nat. Commun. Despite the manuscript is well organized, several concerns still need to be addressed to further improve the quality.
-- The probe BN-O showed hypoxia-responsive and hydrophilic properties, why the authors did not directly inject the probe into mice for tumor-selective theranostic application? Instead, the acid-responsive nanocarrier was used to encapsulate the molecular probe. However, the release profiles of molecular probe from nanocarriers under different pH conditions were not given, which was required to predict the release behavior of BN-O in acidic tumor microenvironment.
-- For in vitro hypoxia responsiveness, the probe was incubated with rat liver microsomes and NADPH system. Why rat liver microsomes + NADPH could mimic the hypoxia condition? For the measurement of hypoxia response to various biospecies, what's the experiment condition?

¬¬¬-- Fig. 2h and Fig. S11, the authors studied the laser power- and concentration-dependent photothermal effect. However, the detailed laser power and concentration for each curve were not given, please clearly label them in the figures. Additionally, the photodynamic process was evaluated using DCFH-DA, ABDA, and DHR123 as the indicators, and the concentrations for each probe should be provided.
-- Fig. 4a-c, the experiment results suggested that the nanoparticles camouflaged with M1 macrophage membrane exhibited higher uptake by 4T1 tumor cells while lower uptake by RAW cells. What’s the reason behind this?
-- In the article, the production of type-I ROS by BC@Z-M after hypoxia activation was demonstrated in solution. As type-I PDT is favorable for hypoxic tumor treatment, the authors are suggested to further assess the type-I ROS generation property of BC@Z-M in cellular level under normoxic/hypoxic conditions.
-- After STING activation, the authors showed an increase in the release of IFN-β, TNF-α, and IL-6 from cells. CXCL10 is a crucial cytokine produced downstream of IFN following STING activation, which facilitates immune cell recruitment. Therefore, assessing the release of CXCL10 post-STING activation is necessary.
-- The in vivo pharmacokinetics and clearance process are important for nanomedicine. Thus, the authors are suggested to investigate the in vivo metabolism property of BC@Z-M after intravenous injection.
-- For the flow cytometry data in the manuscript, the sequential gating/sorting strategies should be provided in the SI.
-- Fig. S31, HIF-a staining was conducted to study the hypoxia conditions after different treatments. But it was labelled as “tumor ROS levels” in the figure caption. Please double check.

Reviewer #4

(Remarks to the Author)
This manuscript reported a hypoxia-activated nanoplatform for imaging and therapy of tumors. Although the results presented in the manuscript prove the conclusion, the manuscript as a whole lacks innovation. First, nitrogen oxides are widely reported to be responded to the hypoxic tumor microenvironment, and there has been much similar work using this property for imaging and therapy (e.g., Advanced Functional Materials, 2019, 29(34): 1903278; Journal of the American Chemical Society, 2023, 145(3): 1707-1713.) In addition, there is no correlation between the two kinds of imaging (NIR-II fluorescence and PA imaging) achieved by the imaging system, which is just a simple multimodal imaging with an archaic concept. Moreover, photothermal therapy (PTT) and photodynamic therapy (PDT) used to enhance the immune response is also a very archaic concept. Therefore, the novelty is not high enough for Nature Communications.

Version 1:

Reviewer comments:

Reviewer #1

(Remarks to the Author)
In the manuscript "Hypoxia-activated and tumor microenvironment remodeling-amplified nanoplatforms for multifunctional imaging and potentiated immunotherapy of cancer” (#NCOMMS-24-24735A), Song and colleagues describe a novel theranostic platform that, upon hypoxia activation, is able to induce tumor-selective immunogenic cell death.
The authors embarked in a extraordinary amount of novel experiments to address the concerns I pose, which are globally cleared. Overall, the manuscript has been greatly improved after the revision and I consider it a very strong candidate for publication in Nature Communication.
I just fount two typos that I suggest to correct: etco-calreticulin should be ecto-calrecticulin.

Reviewer #2

(Remarks to the Author)
The authors have addressed all my concerns, thus I recommend its acceptance in the current state.

Reviewer #3

(Remarks to the Author)
accept

Open Access This Peer Review File is licensed under a Creative Commons Attribution 4.0 International License, which permits use, sharing, adaptation, distribution and reproduction in any medium or format, as long as you give appropriate credit to the original author(s) and the source, provide a link to the Creative Commons license, and indicate if changes were made.
In cases where reviewers are anonymous, credit should be given to 'Anonymous Referee' and the source.
The images or other third party material in this Peer Review File are included in the article's Creative Commons license, unless indicated otherwise in a credit line to the material. If material is not included in the article's Creative Commons license and your intended use is not permitted by statutory regulation or exceeds the permitted use, you will need to obtain permission directly from the copyright holder.
To view a copy of this license, visit https://creativecommons.org/licenses/by/4.0/

Responses to reviewers' comments for the manuscript titled "Hypoxia-activated and tumor microenvironment remodeling-amplified nanoplatforms for multifunctional imaging and potentiated immunotherapy of cancer".

We sincerely thank the reviewers for their positive feedback and valuable comments concerning our article. These comments and suggestions are of great importance to improve the quality of our article. The point-by-point responses to those comments are presented as below and all changes to the manuscript are highlighted by using blue colored text. We hope that the reviewers are satisfied with these changes and the manuscript will be accepted for publication in Nature Communications.

Responses to the Comments and Suggestions of Reviewer #1

In the manuscript "Hypoxia-activated and tumor microenvironment remodeling-amplified nanoplatforms for multifunctional imaging and potentiated immunotherapy of cancer" (#NCOMMS-24-24735-T), Song and colleagues describe a hypoxia-activatable theranostic platform able to induce tumor-selective immunogenic cell death. The message of the article is important, novel and within scope for Nature Communications. Findings are abundant, technically diverse and well presented, and this reviewer had NO concerns about image manipulation or data fabrication. Globally, the authors must be congratulated on the abundant amount of work and quality of presentation. However, some of the conclusions, are not supported by the dataset, and hence must be appropriately revisited (or additional experiments performed in this respect). Alongside, a few experiments are missing that would considerably strengthen the paper, and some minor issues emerged that need to be addressed, as detailed here below. In summary, this reviewer believes the article by Song and colleagues represents a strong candidate for publication in Nature Communications upon revision.

Response: We sincerely appreciate your positive feedback and insightful comments on our work. Your constructive and professional suggestions have greatly enhanced the quality of our manuscript. Below, we provide detailed responses to each of your comments.

Major points:

1) The authors interpret their dataset in several instances as to suggest that STING-IFN-I signaling is responsible for ICD after combined BC@Z-M+L treatment, and openly suggest in the discussion that "The hypoxia-triggered and TME remodeling boosted PTT/PDT attributes jointly activated the ICD and cGAS-STING pathway, engaging both the innate and

adaptive immune system to bolster tumor-specific T-cell immune responses and alleviate immunological resistance". However, the evidences for the cGAS-STING role are circumstantial. The authors show through western blot analysis the patterns of phosphorylation of STING and downstream TBK1 and IRF3 factors, which is indicative but not demonstrative. I recommend to perform in vitro experiments in the gain (overexpression in parental cells) and loss (silencing by shRNAs or knockout by CRIPSR/Cas9) of either STING or IFNAR to show whether STING-IFN-I axis really regulates the immunogenicity of the proposed therapeutic approach.

Response: We sincerely appreciate your constructive suggestions regarding our study. In this work, the "BC@Z-M + L" treatment has the potential to activate both ICD and STING pathways in tumor cells, which work synergistically to enhance the anti-tumor immune response. Following the reviewer's recommendation, we have conducted additional in vitro experiments to further validate the involvement of STING pathway in immune activation.

Specifically, we conducted gain-of-function studies by overexpressing STING in parental cells using STING expression plasmid vector and loss-of-function studies by silencing STING using shRNAs. According to western blot analysis (**Figure 1**), the STING expressions were significantly elevated or reduced following overexpression and silencing, respectively, indicating successful STING expression manipulation. Our results showed that "BC@Z-M + L" treatment promoted the release of IFN-β from 4T1 tumor cells and the subsequent activation of BMDCs. However, STING knockdown in tumor cells obviously impaired the IFN-β secretion and reduced BMDC maturation (**Figure 2**), indicating the crucial involvement of STING pathway in the immune response. The incomplete suppression of BMDC maturation in the STING-knockdown group may be due to the fact that BC@Z-M + L also induces ICD, which could also promote BMDC maturation. Similarly, we found that STING overexpression further amplified both the IFN-β secretion and BMDC maturation induced by BC@Z-M + L treatment. These experiments confirmed that the STING axis indeed regulated the immunogenicity of our proposed therapeutic approach. The results of these experiments have been included in the revised manuscript (Supplementary Fig. 37,41). Thank you once again for your insightful recommendations.

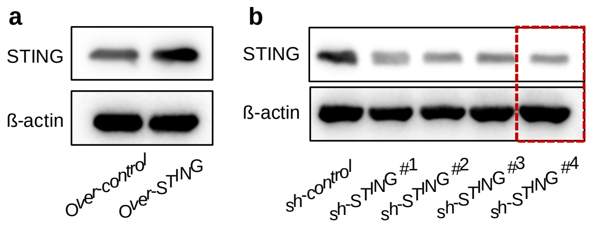

Figure 1. Western blot analysis of STING expression in 4T1 tumor cells following (a) STING overexpression using a STING expression plasmid vector and (b) STING knockdown using shRNAs (four different shRNAs were screened, with shRNA #4 selected for further experiments).

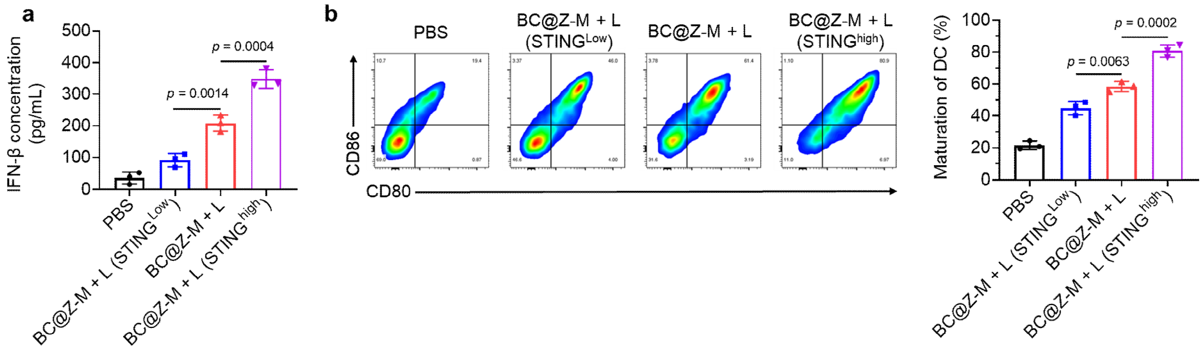

Figure 2. (a) 4T1 tumor cells with normal, knockdown, or overexpressed STING expression were treated with BC@Z-M + L, and IFN-β expression levels in each group were measured using ELISA kit. (b) 4T1 tumor cells with normal, knockdown, or overexpressed STING expression were treated with BC@Z-M + L and subsequently co-cultured with BMDCs in a Transwell system for 24 h. Representative flow cytometry results and quantitative analysis showing the maturation of DCs ($CD80^+/CD86^+$) following different treatments. Data were presented as mean ± SD (*n* = 3 experimental replicates). Statistical significance was determined using one-way ANOVA.

2) The authors show γ-H2AX-related DNA damage after diverse treatments and pose it as the rationale to investigate STING pathway. Have they excluded the effects of other DNA (such AIM2) or even RNA (RIG-I, MDA-5) sensors?

Response: We appreciate the reviewer's insightful question. To address this, we performed silencing experiments targeting AIM2, RIG-I, and MDA-5 using their specific shRNAs. Western blot analysis confirmed the effective knockdown of these sensors (**Figure 3**). Following the knockdown, we assessed the secretion of IFN-β and BMDC maturation in response to our treatments. The results showed that silencing AIM2, RIG-I, or MDA-5 did not significantly alter the secretion of IFN-β and BMDC maturation compared to the control cells (**Figure 4**). However, silencing STING expression markedly reduced IFN-β release and BMDC maturation (**Figure 2** above). These findings suggested that the STING pathway played an important role in the immune response, while AIM2, RIG-I, and MDA-5 might have weak involvement here. The potent PDT and PTT effects induced by BC@Z-M under

hypoxic conditions can cause DNA damage, resulting in their leakage into the cytoplasm. The release of free Zn^{2+} from the nanocarrier can further sensitize the STING pathway by promoting cyclic GMP-AMP (cGAMP) production and increasing the binding affinity between cGAMP and STING (Adv. Mater. 2022, 34, 2206915; ACS Nano 2024, 18, 10439). We have included the new results in the revised manuscript (Supplementary Fig. 42).

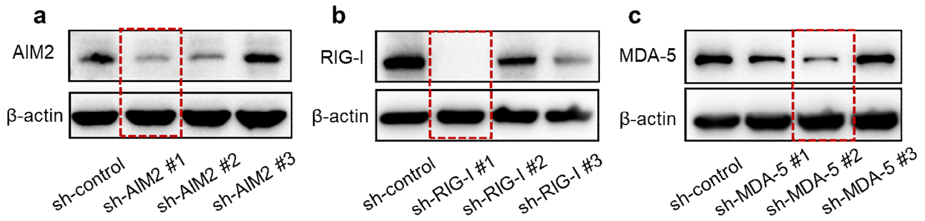

Figure 3. Western blot analysis of (a) AIM2, (b) RIG-I, and (c) MDA-5 expression in 4T1 tumor cells following their knockdown using specific shRNAs. For each target, three different shRNAs were screened, and those highlighted by the red square were selected for further experiments.

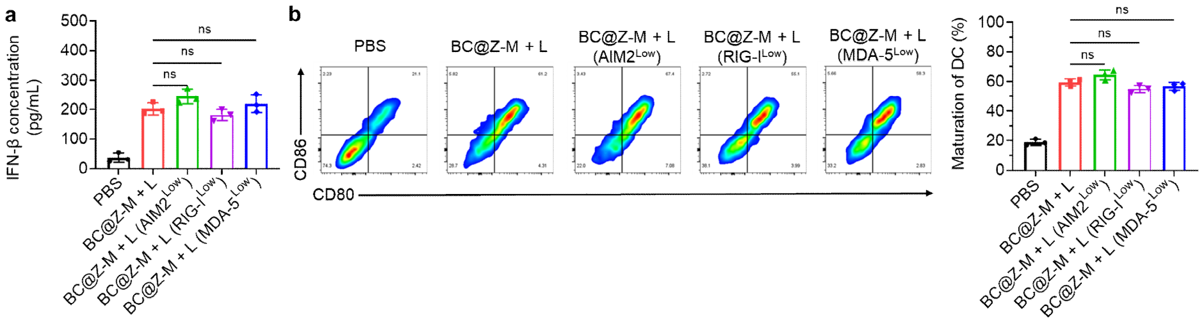

Figure 4. (a) 4T1 tumor cells with AIM2, RIG-I, or MDA-5 knockdown were treated with BC@Z-M + L, and IFN-β expression levels in each group were then measured using ELISA kit. (b) 4T1 tumor cells with AIM2, RIG-I, or MDA-5 knockdown were treated with BC@Z-M + L and subsequently co-cultured with BMDCs in a Transwell system for 24 h. Representative flow cytometry results and quantitative analysis showing the maturation of DCs ($CD80^+$/$CD86^+$) following different treatments. Data were presented as mean ± SD (n = 3 experimental replicates). Statistical significance was determined using one-way ANOVA.

3) According to the author model, BC@Z-M+L is able to induce direct cancer cell killing while activating anticancer immune responses (ICD), which they demonstrate by analyzing

the exposure/release of diverse alarmirs (calreticulin, HMGB1, ATP and IFNs-I). Alongside, they propose the ability of these alarmins to activate dendritic cells by flow cytometry. Are then, these activated dendritic cells able to phagocytose apoptotic cancer cells and to cross-present their antigens to T cells? Are then, these activated T-cells able to kill neighboring cancer cells? The authors should test the immune activation and thus the real immunogenicity of cancer cell death either by in vitro reproducing the crucial steps of the cancer-immunity cycle, or by in vivo vaccinating mice with cancer cells pre-treated with BC@Z, BC@Z+L, BC@Z-M+L or no vaccine and challenging mice with parental cells. Also, it would be great to see the effect in NSG mice, too, to check for a real involvement of the adaptive immune system. Even better, CD4/CD8 depletion should be implemented in immunocompetent mice, as NSG are highly immunodeficient.

Response: We appreciate the reviewer's valuable suggestions regarding the immunogenicity and immune activation. To assess the immune activation and real immunogenicity of cancer cell death, we conducted in vivo prophylactic vaccination experiments following the reviewer's suggestions. As shown in **Figure 5a** below, we vaccinated mice with 4T1 cancer cells pre-treated with PBS, BC@Z, BC@Z + L, or BC@Z-M + L, or no vaccine on day 0, and subsequently challenged them with parental cancer cells on day 7 by subcutaneous injection of live 4T1 tumor cells. Tumor growth at the injection site was monitored every two days. As presented in **Figure 5b**, prophylactic vaccination with 4T1 cells pre-treated by BC@Z-M + L effectively protected the mice against subsequent tumor cell challenge, evidenced by the obviously delayed tumor growth compared to other groups. These results indicated the robust immunogenicity and anti-tumor immunity elicited by cancer cell death induced by BC@Z-M + L. The new results have been added in the revised manuscript (Supplementary Fig. 50).

[panel redacted]

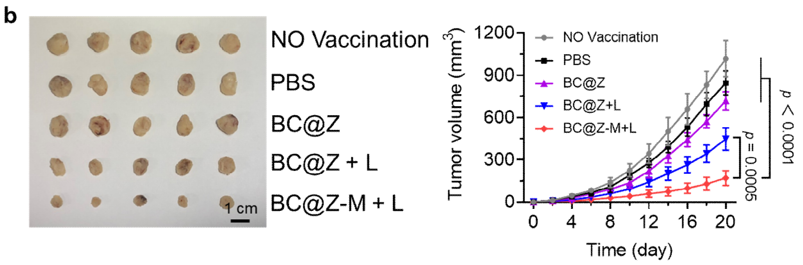

Figure 5. (a) Schematic illustration of the in vivo prophylactic vaccination model and experimental timeline. The illustration was created using BioRender.com. (b) Photographs and volumes of re-challenged right tumors in mice that were immunized with either PBS or

cancer cells pretreated with different treatments. Data were presented as mean ± SD (n = 5 mice). Statistical significance was determined using one-way ANOVA.

Additionally, to evaluate the involvement of adaptive immune system, we performed CD4/CD8 T cell depletion experiments in immunocompetent BALB/c mice by intraperitoneally injecting anti-CD8 and anti-CD4 antibodies. The therapeutic effect of "BC@Z-M + L" in tumor suppression was significantly compromised after depletion of these T cell subsets (**Figure 6**), underscoring the critical roles of T lymphocyte cells in mediating the immune response against cancer. The new results have been added in the revised manuscript (Supplementary Fig. 57).

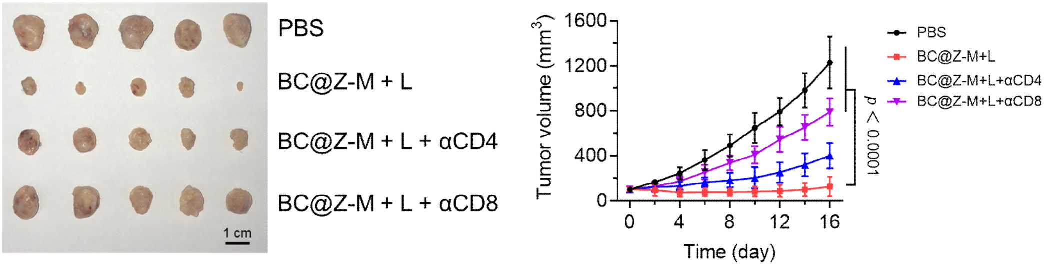

Figure 6. The tumor photographs and tumor volume of 4T1 tumor-bearing mice treated with PBS, BC@Z-M + L, BC@Z-M + L combined with anti-CD4 antibodies, or BC@Z-M + L combined with anti-CD8 antibodies. Data were presented as mean ± SD (n = 5 mice). Statistical significance was determined using one-way ANOVA.

4) It would be great to see a spatial proteomic or even transcriptomic analysis of tumor biopsies to evaluate the type of infiltrating immune cells (pre and post treatment). Also, it would be great to see tertiary lymphoid structures after treatment, but this reviewer acknowledges it may be beyond the scope of the current investigation.

Response: According to the reviewer's suggestion, we have conducted the transcriptomic analysis of tumor tissue to evaluate the types of tumor-infiltrating immune cells before and after treatment. The results revealed substantial alterations in the immune landscape following "BC@Z-M + L" treatment (**Figure 7**). Specifically, there was an increased infiltration of $CD4^+$ and $CD8^+$ T cells in tumor tissue, indicating enhanced anti-tumor immune activation. Additionally, we observed elevated abundances of tumor-infiltrating B cells, dendritic cells, macrophages, and natural killer (NK) cells while reduced levels of

immunosuppressive Treg cells in tumor deposits receiving "BC@Z-M + L" treatment. These findings were consistent with our previous flow cytometry analysis, confirming the role of our treatment in modulating the tumor immune microenvironment and potentiating the anti-tumor immune response. The new results have been added in the revised manuscript (Supplementary Fig. 62).

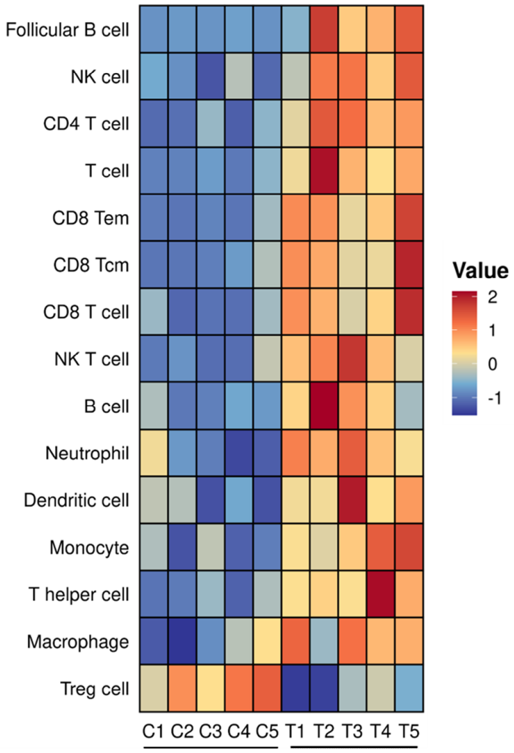

Figure 7. The transcriptomic analyses of various tumor-infiltrating immune cells before (C1-C5 samples) and after (T1-T5 samples) the treatment of BC@Z-M + L.

Regarding the evaluation of tertiary lymphoid structures, while this is beyond the scope of the current investigation, we recognize its importance for assessing immune response and predicting therapeutic outcomes. In our preliminary study, we observed the presence of tertiary lymphoid structures within the tumor of mice treated with BC@Z-M + L (**Figure 8**), characterized by a distinct $CD20^{+}$ B cell adjacent to a $CD4^{+}$ and $CD8^{+}$ T cell zone. The formation of tertiary lymphoid structures can potentially revitalize local anti-tumor immunity and enhance the efficacy of immunotherapeutic treatments. In further research, we plan to conduct a more in-depth analysis of tertiary lymphoid structures to gain more comprehensive understanding of the immune landscape. The new results have been added in the revised manuscript (Supplementary Fig. 63).

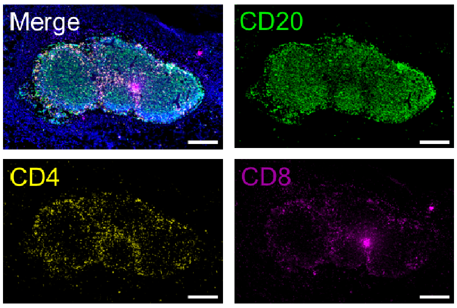

Figure 8. Representative immunofluorescence staining images showing tertiary lymphoid structures in tumor tissue from the mice treated with BC@Z-M + L. Scale bars: 200 μm.

5) The number of experimental replicates for in vitro and in vivo studies is missing. Please provide it.

Response: Thank you for requesting clarification on the number of experimental replicates. For in vitro studies and in vivo bioimaging experiments, each experiment was conducted in triplicate to ensure reproducibility. For the in vivo antitumor study, five mice were included in each group. The replicate numbers have been clearly indicated in the figure legends in the revised manuscript.

6) The authors use 4T1 breast carcinoma cells as the sole cancer model, a highly used triple negative breast cancer model, known to be responsive to immunotherapy. To exclude that their observations are related to the model used, at least in vitro experiments should be performed in other tumor models, maybe of a different background (C57BL/6 mice for example) y comparing polyclonal with monoclonal systems (as the OVA-OT-1).

Response: We appreciate the reviewer's suggestion to validate our findings in additional tumor models. In response, we have conducted in vitro experiments using other tumor cell lines, including the B16-F10 melanoma and MC38 colon carcinoma model (C57BL/6 background). As shown in **Figure 9** below, co-incubation of BMDCs with B16-F10 or MC38 cancer cells pretreated with "BC@Z-M + L" markedly increased the expression of maturation markers CD80 and CD86, indicating strong DC maturation compared to other groups. Additionally, the levels of IFN-β in the supernatant of BMDCs were significantly elevated in "BC@Z-M + L" group compared to other groups. The new results have been included in the revised manuscript (Supplementary Fig. 43).

[figure redacted]

Figure 9. Schematic illustration of the in vitro assessment of BMDC maturation after co-culture with B16 or MC38 cells subjected to various treatments as indicated. Representative flow cytometry results and quantitative analysis of mature DCs ($CD80^{+}/86^{+}$) following different treatments as shown. The expression levels of IFN-β from BMDCs in different groups were also analyzed. Data were presented as mean ± SD (n = 3 experimental replicates). Statistical significance was determined using one-way ANOVA.

We further employed B16-OVA tumor cells, which express the model antigen ovalbumin, to investigate the antigen-specific immune response (monoclonal system) (**Figure 10a**). The B16-OVA cells were treated with various formulations, followed by light irradiation in the indicated groups. These pretreated B16-OVA cells were then co-cultured with BMDCs to assess their maturation and antigen presentation via flow cytometry. The results demonstrated that B16-OVA cells pretreated with "BC@Z-M + L" induced the strongest antigen presentation (DC displaying H-2Kb-SIINFEKL) (**Figure 10b, c**) and BMDC maturation ($CD80^{+}CD86^{+}$ DC) (**Figure 10e, f**) among all groups. Additionally, the type I interferon response, such as IFN-β expression, was significantly elevated in "BC@Z-M + L" group (**Figure 10d**). Given that MHC/antigen cross-presentation and costimulatory signals are essential for inducing tumor-specific cytotoxic T lymphocyte (CTL) responses, we further evaluated CTL activation. BMDCs pretreated with B16-OVA cells subjected to different treatments were co-cultured with $CD8^{+}$ T cells isolation from OT-1 mice, which possess T-cell receptors specific for the ovalbumin-derived peptide SIINFEKL. A carboxy fluorescein succinimidyl ester (CFSE) staining assay was performed to evaluate $CD8^{+}$ T cell proliferation. Significant proliferation of antigen-specific $CD8^{+}$ T cells was observed in the "BC@Z-M + L" group (**Figure 10g, h**). Furthermore, the percentages of IFN-γ^{+} $CD8^{+}$ T cells were greatly elevated after co-culture with BMDCs pretreated with "BC@Z-M + L"-killed B16-OVA cells (**Figure 10i, j**), indicating efficient $CD8^{+}$ T cell activation. We also assessed the ability of these activated $CD8^{+}$ T cells to kill B16-OVA tumor cells by co-culturing B16-OVA cells with activated T cells and using a LDH assay for cancer cell cytolysis evaluation. The results suggested that the activated $CD8^{+}$ T cells exhibited efficient target cell killing activity, with the highest tumor cell lysis and LDH release observed in the BC@Z-M + L group (**Figure 10k**). Our findings confirmed the robustness of our treatment in eliciting a strong immune response in different tumor models. These additional experiments have been included in the revised manuscript (Supplementary Fig. 46).

[figure redacted]

Figure 10. (a) Schematic illustration of evaluating the ability of "BC@Z-M + L"-pretreated B16-OVA cells to activate the immune response, including the promotion of DC maturation and antigen cross-presentation, activation of antigen-specific T cells, and enhancement of T cell-mediated killing of tumor cells. (b) Representative flow cytometry analysis and (c) corresponding quantification of surface antigen (H-2Kb/SIINFEKL complex) presentation on BMDCs after co-incubation with B16-OVA cells pretreated with different formulations. (d) Expression levels of IFN-β in BMDCs across different treatment groups. (e) Representative flow cytometry analysis and (f) corresponding quantification of maturation markers (CD80 and CD86) on BMDCs following co-incubation with B16-OVA cells pretreated with various formulations. (g) Representative flow cytometry analysis and (h) corresponding quantification of CFSE-stained $CD8^+$ T cells in different groups. The proliferation of $CD8^+$ T cells was assessed through CFSE dilution using flow cytometry. (i) Representative flow cytometry analysis and (j) corresponding quantification of the population of $IFN\gamma^+$ $CD8^+$ T cells in different groups. (k) $CD8^+$ T cell-mediated tumor killing was determined by LDH release using a cytotoxicity detection kit. Data were presented as mean ± SD (n = 3 experimental replicates). Statistical significance was determined using one-way ANOVA.

Minor points:

1. Figure 1 is too complicated for an original article and is more appropriate for commentary-like pieces. Please replace with a more schematic picture.

Response: We appreciate the reviewer's feedback regarding the scheme. In response, we have replaced it with a more schematic and simplified illustration. We believe this new schematic makes it easier for readers to understand our core concepts and enhance the accessibility of our scheme.

Responses to the Comments and Suggestions of Reviewer #2

This manuscript introduces a hypoxia-activatable nanotheranostic system with NIR-II fluorescence/PA imaging, PDT/PTT properties, and multifunctional photoimmunotherapy for cancer treatment. The modification of a vascular disrupting agent induces remodeling of the tumor microenvironment, thus amplifying the hypoxia-responsive phototheranostics. Moreover, the probe enables activatable NIR-II fluorescence and PA imaging for tumor delineation, achieving high therapeutic efficacy and immune activation effects in multiple

tumor models. Overall, the work is interesting, and the activatable multifunctional theranostic nanosystem demonstrates high performance in both molecular imaging and immunotherapy. Therefore, it can be considered acceptance in Nature Communications after addressing the following issues.

1. The authors verified the conversion of BN-O into BN in molecular state. Please provide further evidence to demonstrate whether the BN-O encapsulated in BC@Z-M can undergo conversion to BN in hypoxic environment.

Response: We appreciate the reviewer's insightful comment. To address this point, we have conducted additional experiment to investigate the conversion of BC@Z-M under hypoxic stimuli. After BC@Z-M was incubated in hypoxic condition, it was analyzed using high-resolution mass spectrometry (HRMS). The results indicated an obvious conversion of BN-O to BN, with a clear peak corresponding to BN observed in MS spectra. The new result has been included in the revised manuscript (Supplementary Fig. 18).

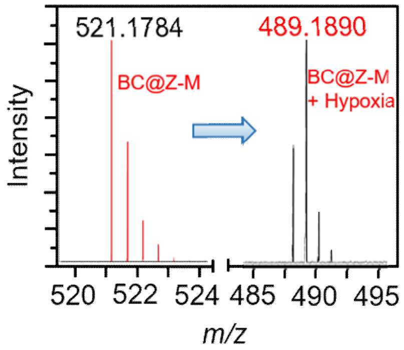

Figure 1. HRMS analysis showing the conversion of BN-O in BC@Z-M to BN following the exposure to hypoxic condition.

2. While the authors indicated that BC@Z-M could efficiently kill tumor cells under light irradiation (Figure 4j), further investigation is needed to examine the extent of tumor cell death and apoptosis following various treatments.

Response: According to the reviewer's suggestion, we further evaluated cell death and apoptosis using the annexin-V and PI co-staining method. Annexin-V staining is commonly used for detecting phosphatidylserine exposure on apoptotic cells, while PI can permeate dead cells and bind to cellular nucleic acids, resulting in intense red fluorescence. Following the treatments of 4T1 cells with different formulations, the cells were stained with Annexin V-FITC and PI and analyzed via flow cytometry. As demonstrated in **Figure 2** below, the

proportion of Annexin V$^+$ and PI$^+$ cells significantly increased after treatment with BC@Z-M under light irradiation, indicating that the "BC@Z-M + L" treatment induced a high level of apoptosis and cell death. These results were consistent with our MTT assay findings, further confirming the potent tumor cell-killing ability of BC@Z-M under light irradiation. The new results have been added and discussed in the revised manuscript (Supplementary Fig. 31,32).

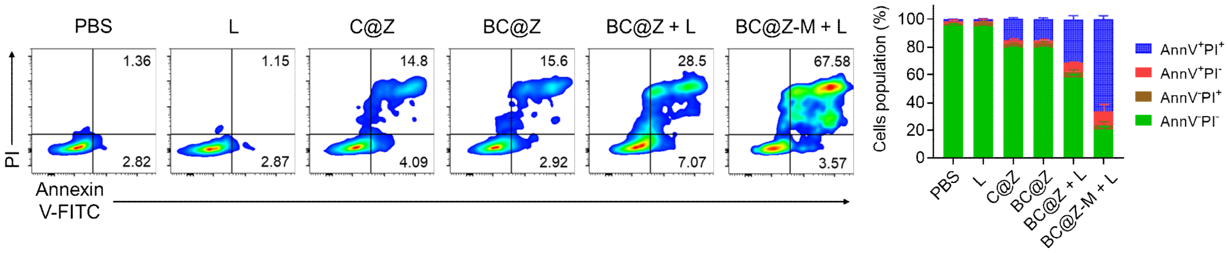

Figure 2. Representative flow cytometry plots and quantitative analyses of the populations of annexin-V and PI co-stained 4T1 cells following various treatments. Data were presented as mean ± SD (*n* = 3 experimental replicates).

3. The intracellular DNA oxidation plays an important role in the activatable PDT/PTT-mediated STING activation. Although the authors studied the mitochondrial damage and DNA damage (Figure 5d-f), the intracellular DNA oxidation was not evaluated.

Response: Thank you for your comment. In the revised manuscript, we have investigated the DNA oxidation using fluorescent antibodies to label 8-OHdG, a well-known biomarker of DNA oxidation. The immunofluorescence staining images (**Figure 3** below) demonstrated that the “BC@Z-M + L” treatment induced significant DNA oxidation, whereas the untreated control, light alone, or nanoparticles alone exhibited only minimal levels of DNA oxidation. These findings suggested that the "BC@Z-M + L"-mediated PTT and PDT could potentially cause DNA damage and oxidation. As reported previously, oxidized DNA showed enhanced stability in the presence of DNase (Immunity 2013, 39, 482), which would facilitate its transport and activate the cGAS-STING signaling pathway. The new results have been added in the revised manuscript (Supplementary Fig. 36).

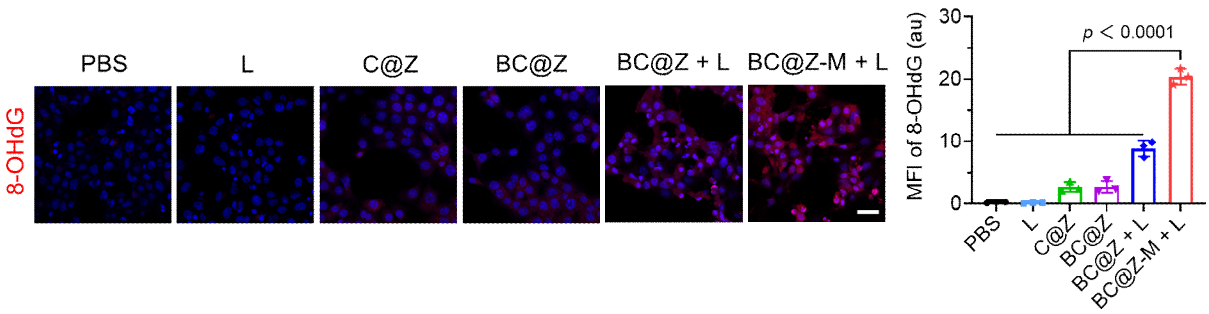

Figure 3. 4T1 tumor cells were treated with the indicated formulations, followed by light irradiation in selected groups. After 24 h, the cells were stained with an anti-8OHdG antibody to label oxidized tumor DNA, and subsequently imaged using confocal microscopy. Scale bar: 50 μm. The corresponding quantitative analysis of the 8OHdG fluorescence intensity was conducted. Data were presented as mean ± SD (n = 3 experimental replicates). Statistical significance was determined using one-way ANOVA.

4. Immunofluorescence staining was utilized to analyze HMGB1 release from cells undergoing ICD, but it did not sufficiently demonstrate the release of HMGB1 into extracellular space. Therefore, I suggest the authors to evaluate the HMGB1 release using ELISA.

Response: Based on the reviewer's suggestions, we further analyzed the HMGB1 released in the cell supernatant using an ELISA assay following the manufacturer's instructions. The concentrations of HMGB1 in different treatment groups were presented in **Figure 4** below, and we found an obvious release of HMGB1 from 4T1 cells following "BC@Z-M + L" treatment, which was consistent with our immunofluorescence staining results. The new results have been added in the revised manuscript (Supplementary Fig. 34).

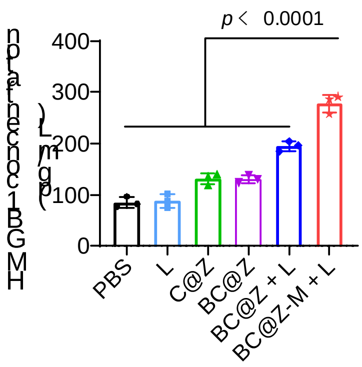

Figure 4. Analysis of HMGB1 concentrations in the supernatant of 4T1 tumor cells after different treatments by ELISA assay. Data were presented as mean ± SD (n = 3 experimental replicates). Statistical significance was determined using one-way ANOVA.

5. The authors studied the signal of BC@Z-M in major organs under NIR-II fluorescence imaging (Fig. 6f). However, the NIR-II fluorescence signal is activated in hypoxia conditions, thus the NIR-II signal in different tissues may not reflect their real levels. Therefore, the authors are suggested to measure the organ distribution of the nanoagent by other method.

Response: We appreciate the reviewer's insightful comment regarding the biodistribution of BC@Z-M. In the revised manuscript, we addressed this by labeling the nanoformulations with a NIR dye, 1,1-dioctadecyl-3,3,3,3-tetramethylindotricarbocyanine iodide (DiR). Using the same method, DiR was loaded into the NPs instead of our probe. At 24 h after intravenous injection, the major organs were collected for ex vivo fluorescence imaging using IVIS. The results indicated that all the nanoagents showed obvious accumulation in the liver due to the unavoidable nanoparticle capture by the reticuloendothelial system, a phenomenon widely reported in the literature. However, the tumors of mice injected with BC@Z-M displayed significantly higher signals compared to those injected with BC@Z lacking cell membrane coating, demonstrating the enhanced tumor-targeting ability mediated by the membrane coating. The new results have been added and discussed in the revised manuscript (Supplementary Fig. 48).

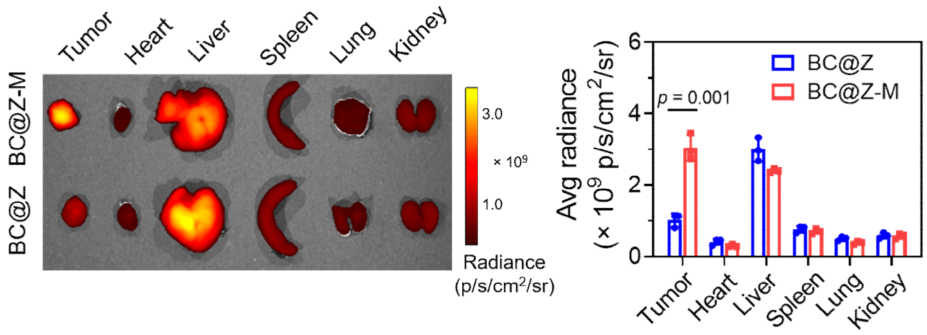

Figure 5. Representative ex vivo fluorescence images of the major organs collected at 24 h after intravenous injection of DiR-labeled BC@Z or BC@Z-M. Quantification analysis of the fluorescence intensity of different organs. Data were presented as mean ± SD ($n = 3$ mice per group). Statistical significance was determined using two-tailed Student's t-test.

6. The authors conducted MTT assay to investigate the cytotoxicity to tumor cells. But how about the biocompatibility of the nanoagent to normal cells? Please supplement some other tests.

Response: According to the reviewer's suggestion, we have further evaluated the cytotoxicity of BC@Z-M towards normal cell lines, including NIH 3T3 (mouse embryonic fibroblast) and HUVECs (human umbilical vein endothelial cells). Following the treatment with varying concentrations of BC@Z-M, the viability of both cell types remained over 90%, indicating good cytocompatibility of BC@Z-M. The new results have been added and discussed in the revised manuscript (Supplementary Fig. 25).

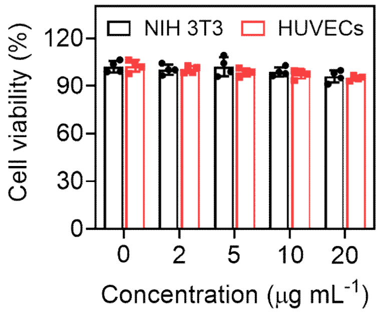

Figure 6. The viabilities of NIH 3T3 and HUVEC cells were assessed using MTT assay following the treatment with varying concentrations of BC@Z-M. Data were presented as mean ± SD ($n = 3$ experimental replicates).

7. Since BN-O is a responsive molecule, and the Synthesis Methods section of Supplementary Material didn't mention whether the synthesis of BN-O was carried out under aerobic or hypoxic conditions, please provide the concentration of oxygen at which BN-O was synthesized.

Response: Thank you for pointing out this. The synthesis of BN-O was carried out under normoxic conditions. We have updated the Synthesis Methods section of Supplementary Information to include this information.

8. How about the photothermal conversion efficiency of BC@Z-M after hypoxia treatment? Please provide additional data regarding this.

Response: In response to the reviewer's comment, we assessed the photothermal conversion efficiency of BC@Z-M after hypoxia treatment. The photothermal conversion efficiency of BC@Z-M following hypoxia treatment was measured as 46.2%. The new results have been added and discussed in the revised manuscript (Supplementary Fig. 22).

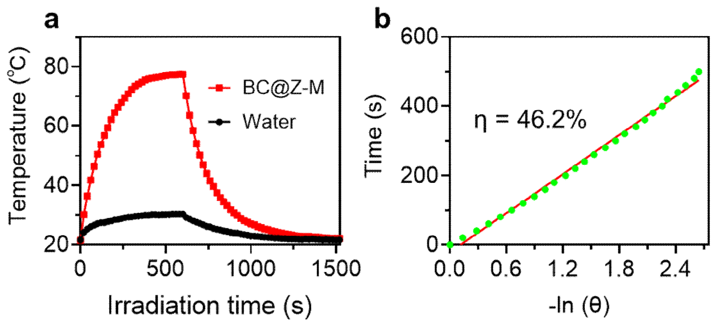

Figure 7. (a) The photothermal behavior of water and BC@Z-M, which were irradiated with 730 nm light for 10 min, then the laser was removed, and the samples were naturally cooling down. (b) Plots of irradiation time versus -ln(θ) for BC@Z-M.

9. Please provide references to support the following statement: "... coupled with the release of Zn^{2+} from the nanocarrier, augmented the activation of STING pathway ...".

Response: In the revised manuscript, we have included several references to support the role of Zn^{2+} in enhancing STING pathway activation, including Adv. Mater. 2021, 33, 2104037; Adv. Sci. 2023, 10, 2302967; Adv. Mater. 2022, 34, 2206915; ACS Nano 2024, 18, 10439.

10. In biosafety evaluation, it is essential to monitor the mice body weight changes after the injection of BC@Z-M into healthy mice.

Response: Thank you for your valuable suggestion. In the in vivo biosafety evaluation, we further monitored the body weight changes of mice following administration of either BC@Z-M or PBS. Our observations indicated no significant differences in weight trends between the mice injected with PBS and BC@Z-M. These findings, combined with histopathological staining and blood indicator results, support the favorable safety profile of BC@Z-M. The new results have been added and discussed in the revised manuscript (Supplementary Fig. 64).

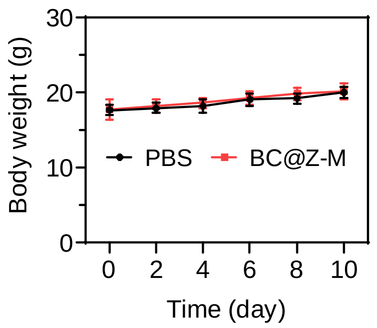

Figure 8. The body weight of healthy mice after the treatment of PBS or BC@Z-M. Data were presented as mean ± SD (n = 3 mice per group).

Responses to the Comments and Suggestions of Reviewer #3

Qi et al. developed a tumor microenvironment-responsive nanoplatform that integrated hypoxia-activatable multimode bioimaging and immunotherapy for the diagnosis and treatment of cancer. In this system, the hypoxia-responsive phototheranostic features of the probe enabled sensitive diagnosis and on-demand treatment, and the vascular disrupting agent further amplified the properties. The turn-on NIR II FL/PA imaging combined with the ICD/STING co-activated immunotherapy were finally achieved.

Given the novelty and comprehensive investigation in this work, I would recommend the acceptance of this manuscript in Nat. Commun. Despite the manuscript is well organized, several concerns still need to be addressed to further improve the quality.

Response: We sincerely appreciate your positive comments, and these constructive and professional suggestions significantly improved the quality of our manuscript. Please find the detailed responses below.

1. The probe BN-O showed hypoxia-responsive and hydrophilic properties, why the authors did not directly inject the probe into mice for tumor-selective theranostic application? Instead, the acid-responsive nanocarrier was used to encapsulate the molecular probe. However, the release profiles of molecular probe from nanocarriers under different pH conditions were not given, which was required to predict the release behavior of BN-O in acidic tumor microenvironment.

Response: Thank you for your insightful question. Both the molecular probe BN-O and vascular disrupting agent CA4P are small molecules, and their direct systemic injection into mice would result in poor blood circulation and inferior tumor selectivity and accumulation. For instance, clinical trials involving vascular disrupting agents have failed to meet expectations, with off-targeting significantly increasing toxic side effects in vivo. To address these issues and enhance the accumulation of BN-O and CA4P at the tumor site, we employed a tumor-targeting nanocarrier to encapsulate these agents.

In the revised manuscript, we have characterized the release profiles of BN-O from the nanocarriers under different pH conditions. To achieve this, we placed the suspension of

BC@Z-M in PBS inside dialysis tubing (Float-A-Lyzer G2 dialysis device, MWCO 100 kDa) and dialyzed it against PBS (pH 7.4 or 6.5) at 37 °C under siring. At different time points, aliquots of the supernatant were withdrawn, and the absorption intensity of BN-O was measured, and its concentrations were then determined using standard curves. The data indicated a more rapid release of BN-O in mildly acidic tumor conditions (pH 6.5) compared to neutral pH conditions (pH 7.4) (**Figure 1** below). This acid-accelerated release profile of BN-O aligns with the on-demand degradation of the nanocarrier and the more rapid release of CA4P and Zn^{2+} observed in mildly acidic environment, indicating the more effective release of molecular probe and drugs in the acidic tumor microenvironment. The new result has been added in the revised manuscript (Supplementary Fig. 23).

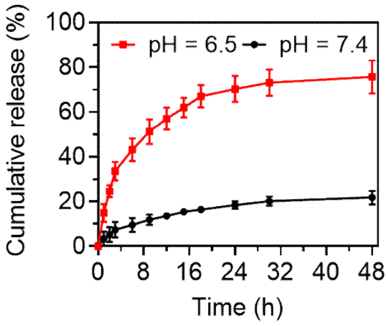

Figure 1. The release profiles of BN-O from BC@Z-M at different pH conditions. Data were presented as mean ± SD (n = 3 experimental replicates).

2. For in vitro hypoxia responsiveness, the probe was incubated with rat liver microsomes and NADPH system. Why rat liver microsomes + NADPH could mimic the hypoxia condition? For the measurement of hypoxia response to various biospecies, what's the experiment condition?

Response: Thank you for your questions. The use of the rat liver microsomes + NADPH system is a well-established method to mimic hypoxic conditions in vitro (Nat. Commun. 2017, 8, 1794; Nat. Commun. 2021, 12, 2772; J. Am. Chem. Soc. 2012, 134, 19588). Rat liver microsomes contain various enzymes, including cytochrome P450 reductase, which, in the presence of NADPH (as electron donor), generates a reductive environment. This environment simulates the low oxygen conditions found in hypoxic tissues, thereby providing a suitable model for studying hypoxia-responsive probes and prodrugs.

The detailed experimental conditions for measuring the probe selectivity to various biospecies, including the concentrations of various components and the reaction time, are clearly included in the figure legends of Fig. 3f in the revised manuscript.

3. Fig. 2h and Fig. S11, the authors studied the laser power- and concentration-dependent photothermal effect. However, the detailed laser power and concentration for each curve were not given, please clearly label them in the figures. Additionally, the photodynamic process was evaluated using DCFH-DA, ABDA, and DHR123 as the indicators, and the concentrations for each probe should be provided.

Response: Thank you for your careful review. We have revised Fig. 2h and Supplementary Fig. 11 to provide detailed labels for the laser power and concentration used in each curve. The updated figures now clearly indicate the specific values for these parameters.

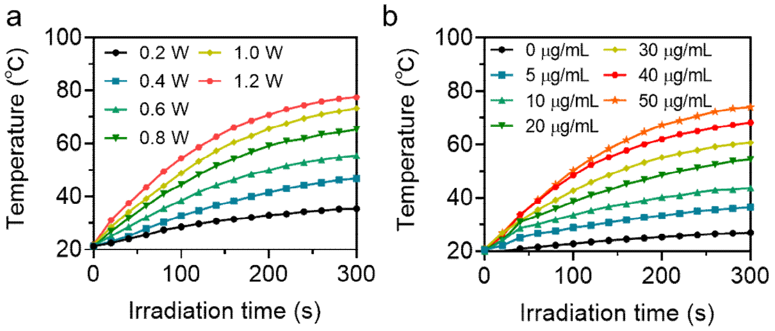

Figure 2. Temperature changes of BN under irradiation with 730 nm light (a) at different power intensities in the presence of BN (50 μg/mL) and (b) at various BN concentrations under 730 nm NIR light (1.0 W cm^{-2}) irradiation.

Regarding the PDT process, we have included the concentrations of each probe (DCFH-DA, ABDA, and DHR123) used in the experiments in the revised manuscript. The concentrations were as follows: DCFH-DA (10 μM), ABDA (10 μM), and DHR123 (10 μM). This information is now provided in the figure legends for clarity.

4. Fig. 4a-c, the experiment results suggested that the nanoparticles camouflaged with M1 macrophage membrane exhibited higher uptake by 4T1 tumor cells while lower uptake by RAW cells. What's the reason behind this?

Response: Thank you for your question. In this work, we found that the macrophage membrane-camouflaged NPs showed increased uptake in 4T1 cancer cells but reduced internalization in RAW 264.7 cells compared with the uncoated NPs. This finding aligned with previous literatures (Nat. Commun. 2020, 11, 2622; ACS Nano 2016, 10, 7738; Biomaterials 2020, 255, 120159; Adv. Healthc. Mater. 2022, 11, e2101788). This behavior is

likely due to the presence of specific proteins on the macrophage membrane, such as CD47, which reduces phagocytosis by binding to SIRPα on macrophages, thereby preventing their uptake of the NPs (Molecular Therapy: Nucleic Acids 2022, 27, 349; Theranostics 2021, 11, 164; Adv. Healthc. Mater. 2022, 11, e2101788; J. Nanobiotechnol. 2022, 20, 542). On the other hand, surface proteins like α4 and β1 integrins on macrophage membrane can bind to VCAM-1 on cancer cells, enhancing the targeting of NPs to tumor site (Clin. Cancer Res. 2012, 18, 5520; Cancer Cell 2011, 20, 538; ACS Nano 2021, 15, 20377). We further performed western blotting analysis, which confirmed the presence of these proteins, including CD47, α4, and β1 integrins, on both M1 macrophage membranes and the coated NPs (**Figure 3**). We have included the new results in the revised manuscript (Supplementary Fig. 26).

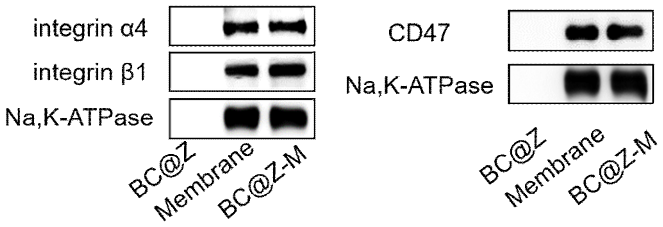

Figure 3. Western blot analysis of the expression of α4, β1 integrins, and CD47 on BC@Z, M1 macrophage membrane, and BC@Z-M.

5. In the article, the production of type-I ROS by BC@Z-M after hypoxia activation was demonstrated in solution. As type-I PDT is favorable for hypoxic tumor treatment, the authors are suggested to further assess the type-I ROS generation property of BC@Z-M in cellular level under normoxic/hypoxic conditions.

Response: Thank you for your suggestion. In response, we have conducted additional experiments to assess type-I ROS generation in 4T1 cells after the treatment with “BC@Z-M + L” under both normoxic and hypoxic conditions. Using HPF (HyPerfluor) as the probe for detecting hydroxyl radicals (•OH), we assessed the intracellular type-I ROS under confocal fluorescence microscopy (**Figure 4**). The results indicated that “BC@Z-M + L” generated significant levels of type-I ROS in hypoxic conditions due to the hypoxia-activated type-I PDT properties of BC@Z-M. These findings further support the potential of BC@Z-M for effective PDT in hypoxic tumors. We have included the new results in the revised manuscript (Supplementary Fig. 29).

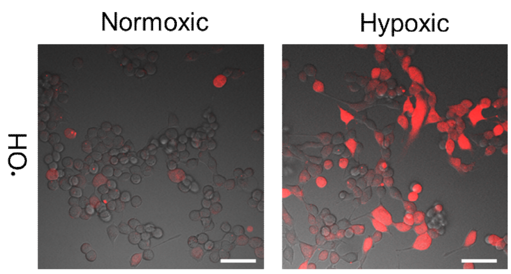

Figure 4. Representative CLSM images showing the type-I ROS generation in 4T1 cancer cells following the treatment with "BC@Z-M + L" under normoxic or hypoxic condition as indicated. Scale bars: 50 µm. HPF (50 µM), a specific probe for hydroxyl radicals (•OH), was used to detect intracellular type-I ROS.

6. After STING activation, the authors showed an increase in the release of IFN-β, TNF-α, and IL-6 from cells. CXCL10 is a crucial cytokine produced downstream of IFN following STING activation, which facilitates immune cell recruitment. Therefore, assessing the release of CXCL10 post-STING activation is necessary.

Response: Thank you for your valuable suggestion. We agree that CXCL10 is an important cytokine in the STING pathway and plays a significant role in immune cell recruitment. In response to the reviewer's suggestion, we have conducted additional experiments to measure CXCL10 levels in the culture medium of 4T1 cells using ELISA following various treatments. The results, shown in **Figure 5** below, indicated a significant increase in CXCL10 release from cells after "BC@Z-M + L" treatment. This finding further supports the activation of STING pathway and the release of crucial immunomodulators for enhancing antitumor responses. We have included the new results in the revised manuscript (Supplementary Fig. 38).

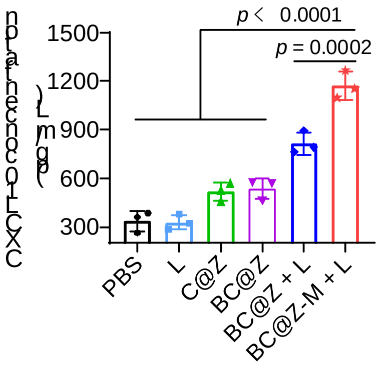

Figure 5. The expression levels of CXCL10 from 4T1 tumor cells after different treatments as indicated. Data were presented as mean ± SD (n = 3 experimental replicates). Statistical significance was determined using one-way ANOVA.

7. The in vivo pharmacokinetics and clearance process are important for nanomedicine. Thus, the authors are suggested to investigate the in vivo metabolism property of BC@Z-M after intravenous injection.

Response: Thank you for your valuable suggestions. In response, we investigated the in vivo pharmacokinetics and clearance process of BC@Z-M following intravenous injection. The DiR-loaded NPs were administered intravenously to mice, and the blood samples were collected at various time points to measure the fluorescence intensity of the NPs. As depicted in **Figure 6a** below, BC@Z-M demonstrated a circulation half-life of about 15 h. Almost all of BC@Z-M were cleared from the bloodstream by 48 h post-injection. For metabolism process evaluation, the feces and urine of mice injected with DiR-loaded BC@Z-M were collected at different time points post NP administration. As shown in **Figure 6b**, significant fluorescence signals were detected in the feces rather than in the urine on days 1 and 3. By day 7, the fluorescence in the feces had markedly decreased, indicating that BC@Z-M was predominantly excreted via the biliary pathway. These findings are in agreement with previous studies (Proc. Natl. Acad. Sci. USA 2008, 105, 1410; ACS Nano 2017, 11, 7177; Adv. Funct. Mater. 2023, 33, 2212380). The detailed results and their discussion have been included in the revised manuscript (new Supplementary Fig. 47,49).

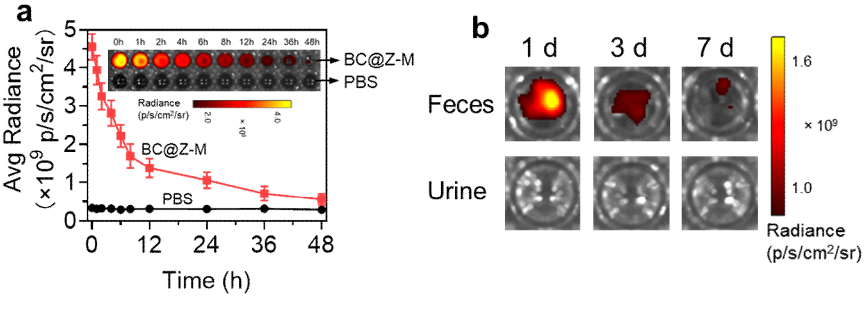

Figure 6. (a) Ex vivo images (inset) and quantified fluorescence intensity of whole blood collected at various time points after intravenous injection with 200 µL of BC@Z-M (1 mg/mL based on BN-O, red line). The black line indicated the fluorescence intensity of blood from the mice injected with only PBS. Data were presented as mean ± SD (n = 3 mice per group). (b) Fluorescence images of the collected feces and urine at various time points after intravenous injection of 200 µL of BC@Z-M (1 mg/mL based on BN-O) into mice.

8. For the flow cytometry data in the manuscript, the sequential gating/sorting strategies should be provided in the SI.

Response: Thank you for pointing this out. We have included the detailed sequential gating and sorting strategies used in our flow cytometry analysis in the Supplementary information of the revised manuscript (Supplementary Fig. 31, 39, 44, 45, 54, 55, and 58-61).

-- Fig. S31, HIF-a staining was conducted to study the hypoxia conditions after different treatments. But it was labelled as "tumor ROS levels" in the figure caption. Please double check.

Response: Thank you for your careful review and for bringing this to our attention. We apologize for the error in the figure caption. We have corrected the caption for Supplementary Fig. 31 (new Supplementary Fig. 52) and updated it to "the detection of tumor hypoxia conditions". The updated caption now properly describes the content of the figure.

Responses to the Comments and Suggestions of Reviewer #4

This manuscript reported a hypoxia-activated nanoplatform for imaging and therapy of tumors. Although the results presented in the manuscript prove the conclusion, the manuscript as a whole lacks innovation. First, nitrogen oxides are widely reported to be responded to the hypoxic tumor microenvironment, and there has been much similar work using this property for imaging and therapy (e.g., Advanced Functional Materials, 2019, 29(34): 1903278; Journal of the American Chemical Society, 2023, 145(3): 1707-1713.) In addition, there is no correlation between the two kinds of imaging (NIR-II fluorescence and PA imaging) achieved by the imaging system, which is just a simple multimodal imaging with an archaic concept. Moreover, photothermal therapy (PTT) and photodynamic therapy (PDT) used to enhance the immune response is also a very archaic concept. Therefore, the novelty is not high enough for Nature Communications.

Response: Thank you for your feedback. We appreciate your comments and it is very important for us to consider these points. We would like to address each of them:

-----Nitrogen Oxides and Hypoxic Response: While *N*-oxide motif has been studied in the context of hypoxia response, our manuscript presents a novel molecular probe with distinct properties and functionalities not reported in existing platforms.

For instance, the study reported in Advanced Functional Materials 2019, 29, 1903278 described a *N*-oxide-based fluorescent probe that showed increased fluorescence at 520 nm under hypoxic conditions, primarily used for in vitro cell imaging. The work published in Journal of the American Chemical Society (2023, 145, 1707) presents a *N*-oxide-based polymer for hypoxia-responsive drug delivery. In contrast, in this work, we developed a novel molecular probe that features turn-on NIR-II fluorescence and photoacoustic (PA) imaging signals, coupled with hypoxia-triggered photodynamic and photothermal therapeutic effects. This probe not only greatly promoted high-contrast in vivo tumor imaging but also activated both the innate and adaptive branches of the immune system to foster antitumoral immunity. To the best of our knowledge, this is the first instance of a single molecular probe integrating hypoxia-activated long-wavelength, deep-penetration multimode bioimaging with multimode photoimmunotherapeutic effects, representing a notable example in advancing the development of high-performing and activatable theranostic protocols.

----Correlation Between NIR-II Fluorescence and PA Imaging: NIR-II fluorescence and PA imaging are both advanced optical imaging techniques, each offering enhanced depth of imaging compared to traditional optical imaging and providing complementary advantages. Specifically, NIR-II fluorescence imaging is renowned for its sensitivity, high signal-to-noise ratios, and minimal background interference, though it faces challenges in deep tissue penetration and spatial resolution. On the other hand, PA imaging excels in spatial resolution and deep tissue penetration but has limitations in sensitivity.

By combining NIR-II fluorescence with PA imaging in this work, we leverage the strengths of each modality to achieve a comprehensive assessment of tumors. We developed a novel molecular probe that simultaneously activates bright NIR-II fluorescence and PA signals in response to hypoxic conditions, and further demonstrated how this combination offers complementary advantages for thorough evaluation of tumor. Specifically, NIR-II fluorescence imaging provides precise tumor localization with high signal-to-noise ratio, while PA imaging offers valuable details on tumor size, morphology, and distribution pattern due to its superior penetration depth and spatial resolution. Although the NIR-II fluorescence and PA imaging were conducted using two imaging systems, given the current lack of commercial systems that support concurrent capture of both signals, our molecular probe with activatable NIR-II fluorescence and PA imaging capabilities provides comprehensive insights into tumor characteristics. We believe that the development of optical imaging

equipment capable of simultaneously capturing both NIR-II fluorescence and PA signals in the future will further enhance the convenience and scope of such advanced imaging probes.

-----PTT and PDT for Immune Response Enhancement: Although PTT and PDT have been employed for tumor immunotherapy, our platform addresses several critical challenges faced by current systems. Firstly, unlike traditional "always-on" photosensitizers that may pose risks of photodamage to surrounding healthy tissues and skin, our molecular probe exhibits hypoxia-triggered PDT and PTT effects, enabling precise, on-demand photoimmunotherapy activation. Moreover, the PDT process activated by our probe primarily operates via a type-I mechanism, which is less dependent on oxygen compared to type-II PDT, making it particularly advantageous for the treatment of hypoxic solid tumors. Furthermore, we have clearly demonstrated the capability of concurrently activating robust PDT and PTT effects to foster antitumoral immunity by promoting the activation of the ICD and STING pathways and effectively engaging both the innate and adaptive immune system. What is more, we further augmented the hypoxia-activated molecular probe with a tumor-targeting and tumor microenvironment-remodeling nanocarrier to amplify the hypoxia-responsive phototheranostics.

In summary, we believe that our manuscript presents notable advancements by combining precise activation with multimodal imaging and synergistic potent immunotherapy, which collectively enhance the overall therapeutic and diagnostic potential for hypoxic tumors. According to the reviewer's comments, we have revised the manuscript to highlight these novel aspects more clearly and have included additional discussions to emphasize the unique contributions of our work (Page 27-28). Thank you again for your thoughtful review. We hope the revised manuscript addresses these concerns adequately.

We sincerely thank the reviewer for his/her precious time and recognition of our work. Below are our point-by-point responses to the reviewer’s comments.

Responses to the Comments and Suggestions of Reviewer #1

Reviewer #1 (Remarks to the Author):

In the manuscript "Hypoxia-activated and tumor microenvironment remodeling-amplified nanoplatforms for multifunctional imaging and potentiated immunotherapy of cancer” (#NCOMMS-24-24735A), Song and colleagues describe a novel theranostic platform that, upon hypoxia activation, is able to induce tumor-selective immunogenic cell death.

The authors embarked in a extraordinary amount of novel experiments to address the concerns I pose, which are globally cleared. Overall, the manuscript has been greatly improved after the revision and I consider it a very strong candidate for publication in Nature Communication.

I just fount two typos that I suggest to correct: etco-calreticulin should be ecto-calrecticulin.

Response: We sincerely thank the reviewer for your positive feedback and support for the publication of our study. In the revised manuscript, we have corrected the word etco-calreticulin. We have also carefully checked the manuscript to avoid typos.

Responses to the Comments and Suggestions of Reviewer #2

Reviewer #2 (Remarks to the Author):

The authors have addressed all my concerns, thus I recommend its acceptance in the current state.

Response: We sincerely thank the reviewer for your positive feedback and support for the publication of our study.

Responses to the Comments and Suggestions of Reviewer #3

Reviewer #3 (Remarks to the Author):

accept

Response: We sincerely thank the reviewer for your positive feedback and support for the publication of our study.
